# Hybrid encryption technique: Integrating the neural network with distortion techniques

**DOI:** 10.1371/journal.pone.0274947

**Published:** 2022-09-28

**Authors:** Raed Abu Zitar, Muhammed J. Al-Muhammed

**Affiliations:** 1 Sorbonne University Center of Artificial Intelligence, Sorbonne University-Abu Dhabi, Abu Dhabi, U.A.E.; 2 Faculty of Information Technology, American University of Madaba, Madaba, Jordan; Khon Kaen University, THAILAND

## Abstract

This paper proposes a hybrid technique for data security. The computational model of the technique is grounded on both the non-linearity of neural network manipulations and the effective distortion operations. To accomplish this, a two-layer feedforward neural network is trained for each plaintext block. The first layer encodes the symbols of the input block, making the resulting ciphertext highly uncorrelated with the input block. The second layer reverses the impact of the first layer by generating weights that are used to restore the original plaintext block from the ciphered one. The distortion stage imposes further confusion on the ciphertext by applying a set of distortion and substitution operations whose functionality is fully controlled by random numbers generated by a key-based random number generator. This hybridization between these two stages (neural network stage and distortion stage) yields a very elusive technique that produces ciphertext with the maximum confusion. Furthermore, the proposed technique goes a step further by embedding a recurrent neural network that works in parallel with the first layer of the neural network to generate a digital signature for each input block. This signature is used to maintain the integrity of the block. The proposed method, therefore, not only ensures the confidentiality of the information but also equally maintains its integrity. The effectiveness of the proposed technique is proven through a set of rigorous randomness testing.

## Introduction

Information security consists of three fundamental pillars: confidentiality, integrity, and availability [1]. Maintaining these three pillars is a must for full and true information protection. It is a bit surprising that most of the work is dedicated to maintaining confidentiality only (through encryption techniques) but ignoring the other security pillars. Reviewing the literature, one can find too many encryption techniques that use different computational models to encrypt plaintexts. In [2], authors proposed a dynamic approach for encryption in which a static knowledge of symbols and mapping tools are defined, but also in which the computations of the algorithm, and consequently the encryption, is dynamically controlled by adjusting the behavior of the operations. In [3] authors proposed an encryption technique, which used a random generation function whose computational behavior depends on both plaintext and key. In [4], authors proposed a chaotic-based approach in which the chaotic number system induces large confusion in the resulting ciphertext. In [5, 6], authors provided a deep analysis of the performance of many DNA-based techniques. In [7], authors proposed DNA based encryption technique. This technique depends on the complexity of DNA sequences to hide the ciphertext symbols. In [8], authors proposed an image encryption technique that depends on merging between DNA substitution and shuffling techniques for achieving high confusion. Using several one-dimensional chaotic systems was also proposed in [9]. In this approach, the output of these chaotic systems was combined to maximize the confusion. In [10] authors proposed a dynamic encryption technique in which the confusion is maintained by dynamically changing some of the algorithm parameters. In [11], authors proposed a highly nonlinear substitution technique augmented with effective key and plaintext based distortion methods. In [12], authors proposed a neural network-based image encryption technique augmented with chaotic noises for maximum resistance against attacks. The advanced encryption standard AES [13–15], Blowfish [16], Serpent [17], MARS [18], and Data Encryption Standard DES [19, 20] along with its variations [21] all use the substitution and shifting operations along with the key rounds to produce enough confusion that maintains the confidentiality of the information. Other encryption techniques depend mostly on complicated mathematical operations for protecting the privacy of the information (e.g. RSA [22, 23], RC_5_ [24, 25], HiSea [26]).

Very important techniques were proposed in the image encryption arena. Color image encryption based on one-time keys and robust chaotic maps was proposed in [27]. This encryption technique used a linear piecewise chaotic map to generate the keys with a real random generator. In [28], the authors proposed a similar method but augmented with a bit level permutations. An encryption technique based on chaotic maps along with DNA coding was also proposed in [29]. Perceptron model was incorporated for encryption in [30]. In [31], computational model for simultaneous picture encryption methods using a proposed permutation and parallel diffusion is presented. A dynamic coupling coefficient with a mapping lattice is used for private images [32]. In [33], a semi-tensor product matrix is used to create chaotic encryption with a secret key. A Boolean network with image encryption is used with a semi-tensor matrix [34], comparative results were achieved. Fractal Sorting matrix (FSM) with global chaotic pixel diffusion is used in [35]. The R, G, and B components of a pixel are encoded based on chaos theory in [36]. Nonadjacent coupled lattices are used in image encryption in [37]. DNA sequence is used in encoding the jumbled image, also chaotic pseudorandom sequences are generated in [38]. Hybrid chaotic mapping and dynamic random growth techniques are used in [39]. All the previously mentioned techniques proved their ability to resist many types of attacks.

Despite their importance, all these methods regardless of their computational models and input type (text or image) address only one pillar of security: confidentiality. Although information confidentiality is extremely important, researchers realize that maintaining information integrity is equally important and should not be overlooked [40]. Many techniques, therefore, have been proposed to effectively maintain information integrity. Examples of such techniques include MD5 [41], SHA-x [42, 43], Whirlpool [44], and BLAKE2 [45]. These techniques produce a unique security code (typically a 512-bit hashing value) for plaintext and this security code is used as a reference for checking if information integrity has been tampered with. Additionally, compared to the state-of-the-art techniques, the proposed technique presents a unique method that combines computational processing (FF neural learning by weights adjustment) along with symbolic substitution. Using this combination, the proposed technique achieves high diffusion and confusion by the sequence of keys generation (weights of the neural network) and the highly illusive table look up method (the symbolic approach). All are accompanied by another RNN to generate hashing values to guarantee and verify integrity of data.

This paper offers a hybrid encryption technique that addressed both information security and integrity. The proposed technique combines the non-linear complicated transformation of the neural network with the sophisticated distortion operations. The hybrid technique makes use of a two-layer feedforward neural network. The first layer is an encryption layer, which is trained on each input block of the plaintext. This training yields a set of weights (play the role of the encryption key) that are used to encrypt the input block. The second layer generates a new set of weights for canceling the impact of the first layer and recovering the plaintext block. Besides these two layers, the encryption technique includes also a recurrent neural network (RNN) that is executed in parallel with the feedforward neural network layers to generate a security code. The generated security code is used for maintaining the integrity of the input block. The distortion operations twist further the output of the encryption layer (the first layer). The distortion operations use random noises and transformation actions such as symbol substitution, swapping, and bit flipping to greatly maximize the confusion of the resulting ciphertext.

To the best of our knowledge, we are not aware of any other encryption technique that addresses both confidentiality and integrity in a single and coherent method. All of the techniques that we know of either address confidentiality or integrity, but not both. This paper addresses both and makes the following contributions.

1. An effective hybrid encryption technique that addresses the two key pillars of information security: confidentiality and integrity.
2. The technique uses the connectionist approach (neural network) whose output is a pre-ciphertext with no correlation to the input plaintext
3. The proposed technique combines the random-induced noise with the tricky manipulation operators into a coherent distortion layer that imposes high confusion in the ciphertext.
4. The technique ensures the integrity of each individual block by generating a hash value for it. The generation of the hash value incurs no additional processing time since it is pegybacked with the training time when encrypting the block.

## Neural encryption/decryption models

**Fig 1:**
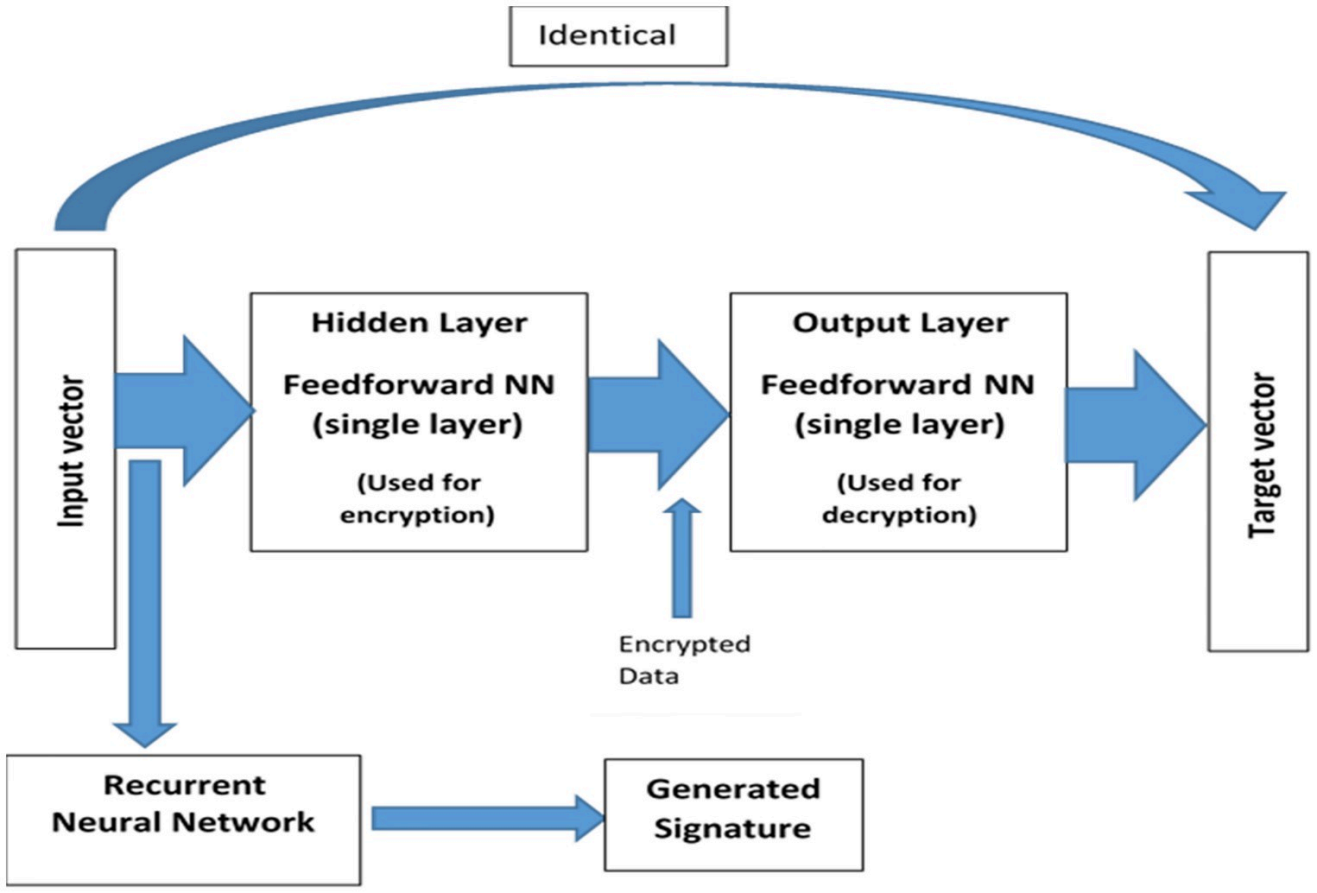
The mirror two-layered *NN* used for encryption/decryption and signature.

**Fig 2:**
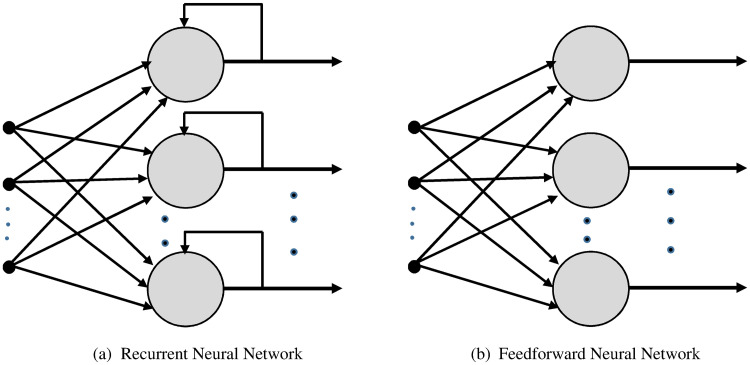
An architecture of recurrent and feedforward neural networks. (a) Recurrent Neural Network, (b) Feedforward Neural Network.

The proposed encryption technique uses artificial neural network techniques [46–50]. In particular, we exploit the long-established two-layer supervised feedforward neural network (please see Fig 1). This neural network (*NN*) is trained to function as a mirror, where the input vector equals the target vector. Fig 2 shows the architectures of the feedforward *NN* and the *RNN*.

**Fig 3:**
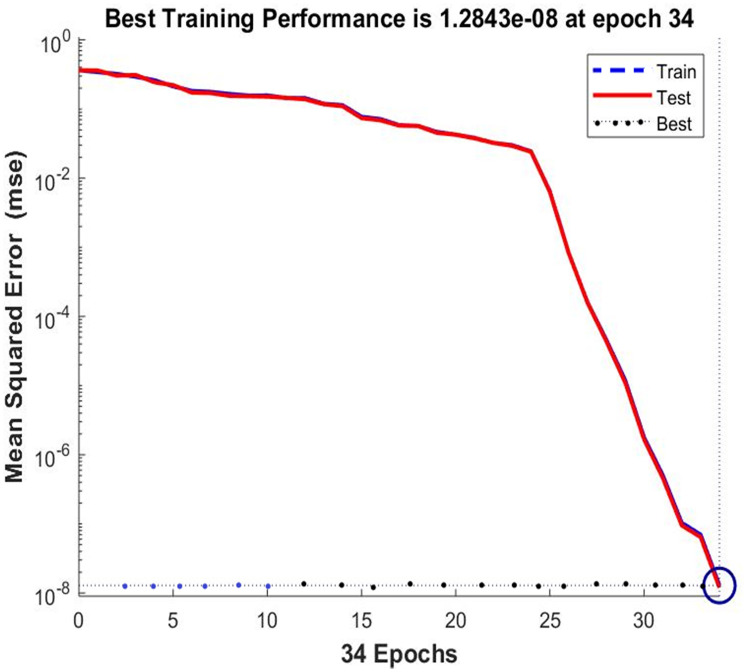
Training error versus epochs (an epoch is a whole batch of input vectors).

The training stage includes finding a set of weights for the hidden layer and a set of weights for the output layer such that the input vector equals the target vector. In our approach, we trained the neural network for each of the 256 ASCII characters. We used Genetic algorithms to minimize the mean squared error, which represents the difference between the input vector and the target vector, until this error reaches the pre-specified threshold. (Unlike other training algorithms such traditional gradient descent based algorithms, Genetic algorithms can converge faster to the desired global optimum and do not easily get caught in a local optimum.) When the error is less than the pre-specified threshold, the algorithm converges to the right two sets of weights for both layers and the neural network becomes a mirror. These weights (for both layers) are stored along with the input vector (or the ASCII character). The weights for all the 256 characters create the key file. The key file must be kept secret and communicated in a secure fashion. (Communicating the weights file (the key) is similar to communicating the encryption key in conventional ciphers. We, therefore, use the same methods such as [51] for secure key communication.) Note, the communicated key file can be used by all the communicating parties to encrypt plaintext using the weights of the hidden layer and also used for decryption ciphertexts using the weights of the output layer. Fig 3 provides an example of the training and testing processes for the feedforward neural network (mirroring).

The *NN* performs the encryption blockwise. The hidden layer of the neural network encrypts the symbols of the input block and produces a corresponding ciphertext. The encryption for each character is done using the weights of the hidden layer associated with this character. In particular, we combine these weights by multiplying each weight with the character and sum these multiplications. The decryption of the characters of the ciphertext block is performed using the sets of the output layer’s weights associated with the characters. Specifically, we multiply each weight with the ciphered character and sum the multiplications. Because our feedforward neural network functions as a mirror, the output layer can successfully recover the original plaintext block, provided of course that the right weights are given. Note, there is no need to train the neural network during the decryption: just use the weights of the output layer in the key file.

Before leaving this section, we would like to comment on the training time. According to our simulation, the training time is very negligible. Training our two-layer feedforward neural network on the 256 ASCII characters required less than one second when done on Intel Core *i*7 computer with Matlab [52]. The average training time for 100 different random initializations of weights was approximately 1.0313 seconds. This average is very reasonable given that we can reuse the produced weights to encrypt any number of plaintexts. In addition, we expect the average time to remarkably decrease if more powerful hardware and assembly language (instead of Matlab) are used.

## Recurrent neural network

The proposed encryption technique uses a recurrent neural network to create a signature (a hashing value), which is used for maintaining the integrity of the plaintext block [50]. The *RNN* exploits both the semantics of the input characters and their texture (order in the input) to create a unique signature for the input block. The feedbacks to the *RNN*, as shown in Fig 2, force the state of RNN to be a function of the current input and the previous inputs. This feature of our proposed RNN enables it to detect any changes to the input characters and react by drastically changing the signature for this block. Therefore, two different blocks will receive different signatures even if they differ in only one bit.

The *RNN* consists of 8 neurons each is associated with a different random weight obtained from the random generator (discussed next). Each neuron uses its associated random weight along with the input character to generate one real number for this input character. The neuron updates its output (the real number) when it receives a new input character. The update is done by applying a simple linear function whose output can be any value within the interval [–1, +1]. When all the characters of the input block are processed, the output of 8 neurons are combined to create the final signature for the input block. (Since *RNN* depends only on the input characters and the initial random weights to change its output, no training is required for the *RNN*).

For example, we applied *RNN* to the following text. *Sport includes all forms of competitive physical activity or games which, through casual or organized participation, aim to use, maintain or improve physical ability and skills while providing enjoyment to participants, and in some cases, entertainment for spectators. Hundreds of sports exist, from those between single contestants, through to those with hundreds of simultaneous participants, either in teams or competing as individuals*. The *RNN* generated the hashing values ‘04A8BE’, ‘0705F3’, ‘0BBCED’, and ‘040004’.

We conclude this section by presenting a simple but informative comparison between RNN hashing and two standard hashing algorithms: MD5 and SHA-x [41, 43] (see Table 1). The numbers in Table 1 shows that RNN has better performance especially with respect to the collision detection (very important feature for any hashing technique).

**Table 1:** The recurrent NN performance.

Parameters	RNN1	RNN2	MD5	SHA-1
Block size (characters)	1000	1000	512	512
Message Digest size (characters)	10	10	128	160
Word size (characters)	10	10	32	32
Collision found	None	None	Yes	Theoretical attack
Performance (Cycle per byte)	9.9	10.8	4.99	3.47

## Two–layer neural network cipher

**Fig 4:**
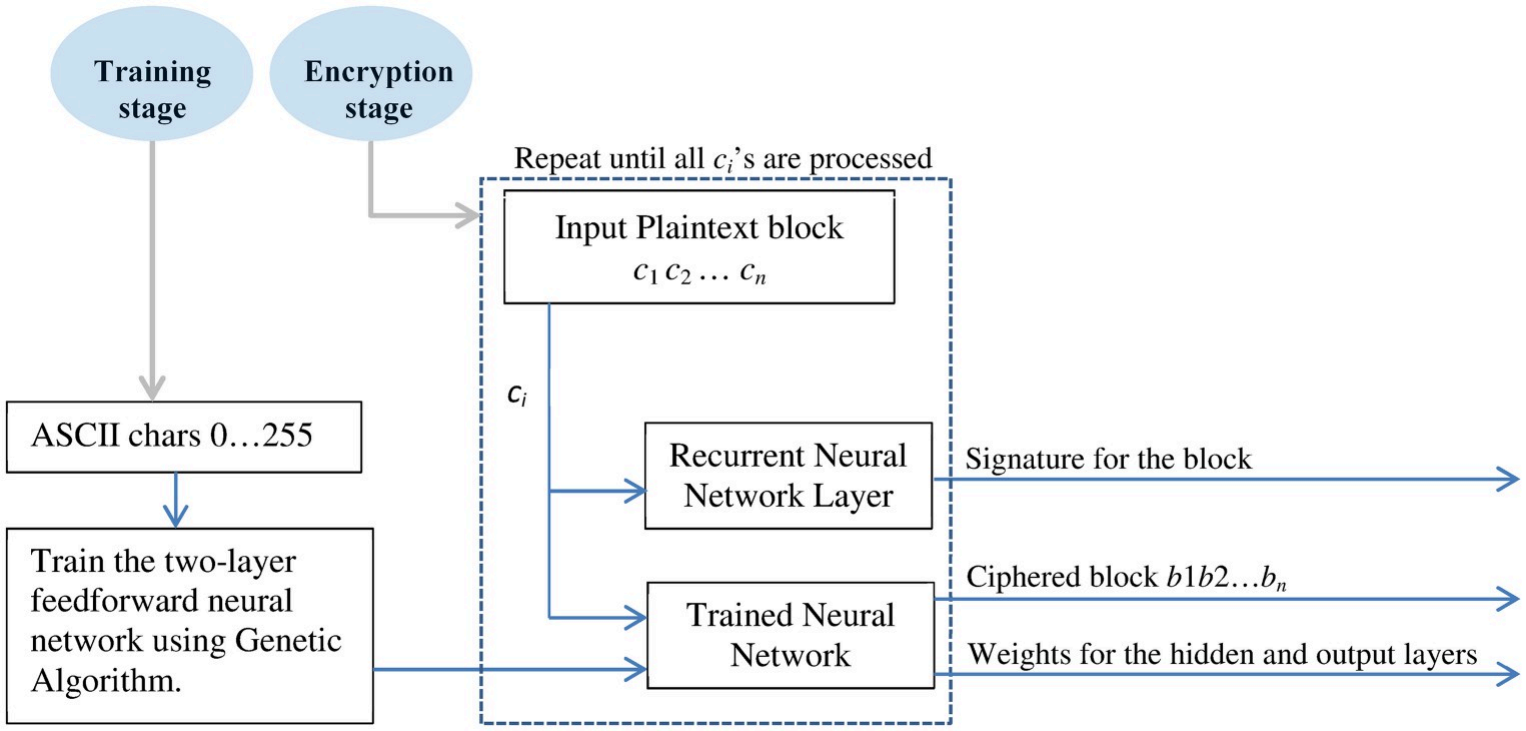
The encryption process using *NN*.

Fig 4 describes the two-layer feedforward neural network cipher (*NN*). The cipher must be trained before it can be used for encryption or decryption. As previously pointed out, the cipher is trained for each of the 256 ASCII characters to function as a mirror for each character. Once trained, the weights are stored and used as a key for encrypting or decrypting any number of plaintexts. Of course, the training can be redone with new random weights to produce a totally different key if there are security concerns about the key (for example learned by an unauthorized party).

The cipher encrypts the *n*–symbol blocks of plaintext using the key (the weights of the hidden layer). In particular, the input character *c*_*i*_, is encoded using the associated weights with this input character (*c*_*i*_) to produce the ciphered character *b*_*i*_. In parallel, the character *c*_*i*_ is also passed to the recurrent neural network (*RNN*) to produce a signature for the character *c*_*i*_. The encryption process is repeated until all the block symbols *c*_*i*_ are encoded and the signature for the symbols *c*_*i*_’s are accumulated to produce the final signature for the entire block symbol. The ciphered blocks *b*_1_*b*_2_…*b*_*n*_ are combined into one pack. The entire ciphertext and the signature produced by recurrent neural network are sent over the network. The recipient can straightforwardly decrypt the ciphertext and check the integrity provided that we have already communicated the key file with this recipient. The decryption is easy once the key available. The two sets of weights (hidden and output) are used to rebuild identical two–layered feedforward neural network. Since we use the same weights (used during the encryption), the reconstructed *NN* is necessarily identical to the original one. Therefore, it can successfully recover the corresponding plaintext block using the output layer. Furthermore, the weights associated with the *RNN*’s output, are used to reconstruct identical *RNN*, which will be used to recompute the signature of the restored block and compare the recomputed signature with the sent one.

## Distortion process

**Fig 5:**
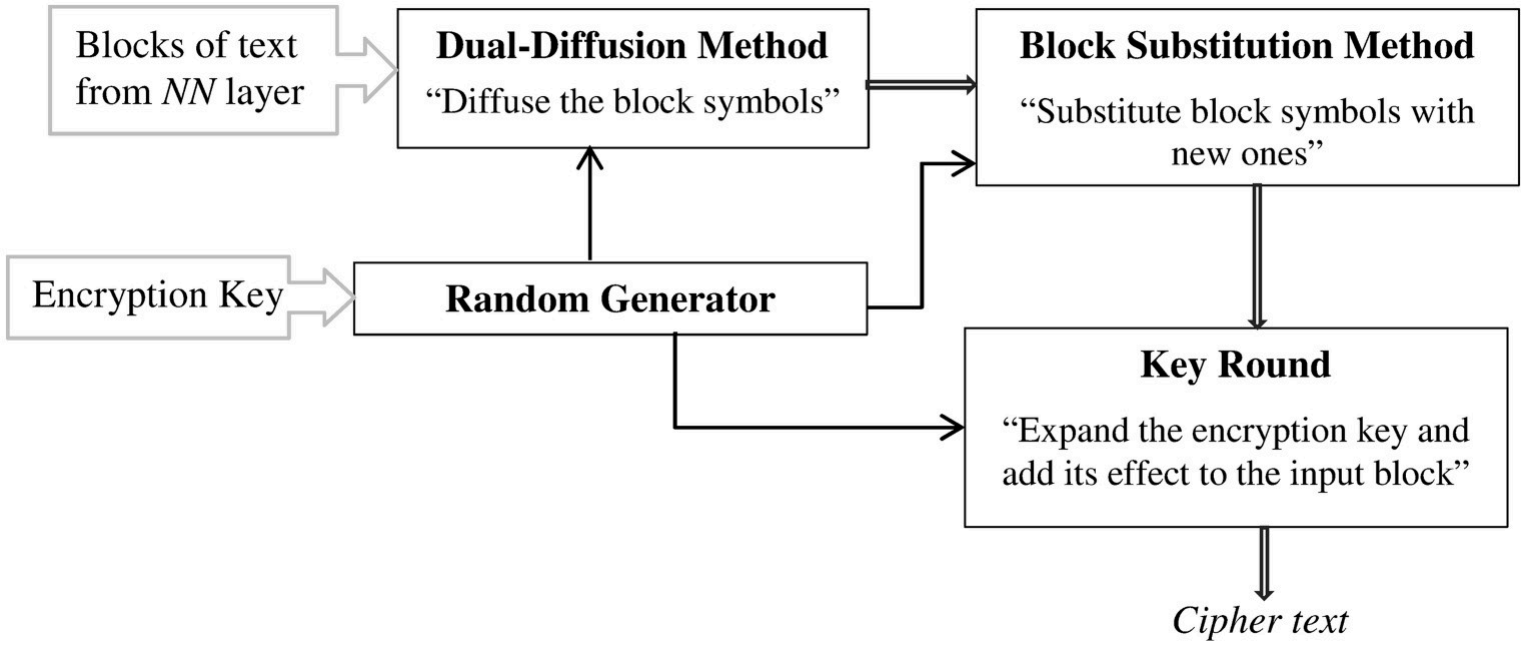
The control flow of the distortion layer.

The distortion process imposes additional confusion on the ciphertext produced by neural network. Each input symbol of the ciphertext is distorted using effective distortion operations. The distortion operations function synergistically to greatly boost the confusion by cutting the relations between the blocks of the input plaintext and the resulting ciphertext. Fig 5 outlines the flow of control between the distortion operations. As it could be seen, the input is first processed by the dual-diffusion method. This method sniffs changes in the input block symbols and propagates these changes to impact every bit in the block. The output of this operation is next processed by the block substitution method, which uses a substitution table. The distortion process further distorts the block by mixing its symbols with highly complicated codes generated by expanding the encryption key through an effective expansion method. The random generator provides unpredictable, though reproducible, random numbers to support the functionality of the three distortion methods and maximize their effectiveness. The following subsections discuss the technical details of these distortion operations.

### Random generator

**Fig 6:**
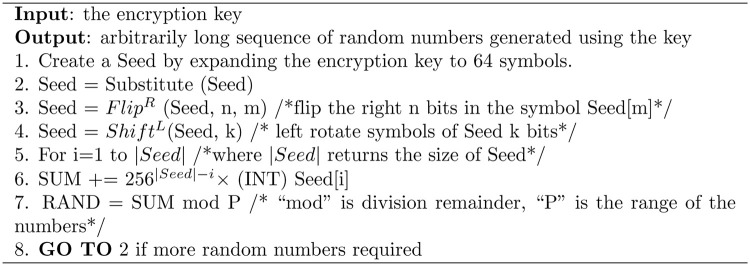
The algorithmic steps of the random generator.

This section describes a random number generator, which is adopted from [8] and whose algorithmic steps are reproduced in Fig 6. The random generator first expands the key to 64 bytes (symbols). The intuition is that large seeds lengthen the period of the generator so that it can produce long sequences without repeating the same sequence. The key extension method uses Substitute and Manipulate operations whose technical details are beyond the scope of this paper and can be found elsewhere [53]. The random number generation generates its output by repeating steps (2) through (7). Before generating any random number, all the symbols of the seed are substituted in step (2) using S–BOX (described next). This step is extremely important since it (*i*) refreshes the seed symbols and weakens the relationship to the input seed and (*ii*) ensures high changes to the new seed if any bit in the original seed changes. The operator *Flip*^*R*^ (step 3) flips the right *n* bits of the seed’s symbol at the index *m*. The values of *n* and *m* are calculated from respectively the seed symbols at indexes 0 and 1. That is, *n* = (INT) Seed [0] and *m* = (INT) Seed [1]. The *Shift*^*L*^ operator left rotates the bits of the Seed by *k* bits. The value of *k* is not prespecified and it is the integer value of the symbol Seed [2]. The main objectives of the steps (3) and (4) are to deeply change the Seed from one hand and to increase the effectiveness of the Substitute operation on other hand. Once the seed is prepared, the random numbers are generated using steps (5) and (6), where the integer values of the seed’s symbols are summed up by multiplying the integer value of the Seed symbol at index *i* with the power of 256.

There are many security advantages to adopting this random generator. First, as described in [8], the seed is entirely based on the encryption key. This means that the sequence of the generated random numbers depends on the key. Second, the generator is very sensitive to the encryption key. Minor changes to the key result in very different sequences of random numbers. Third, it produces extremely long sequences of random numbers without any repeated patterns. This is a very important property to ensure that the patterns that naturally appear in the input text (plaintext) are greatly removed. Fourth, the generator uses simple steps (see Fig 6). It is, therefore, highly efficient and does not require significant CPU power or memory. These advantages make the random generator an ideal choice for this paper. Fifth, the resulting random numbers have a very important feature: they are greatly independent due to the deep manipulation of the seed using flip, shift, and substitution operators.

### Symbol substitution method

**Fig 7:**
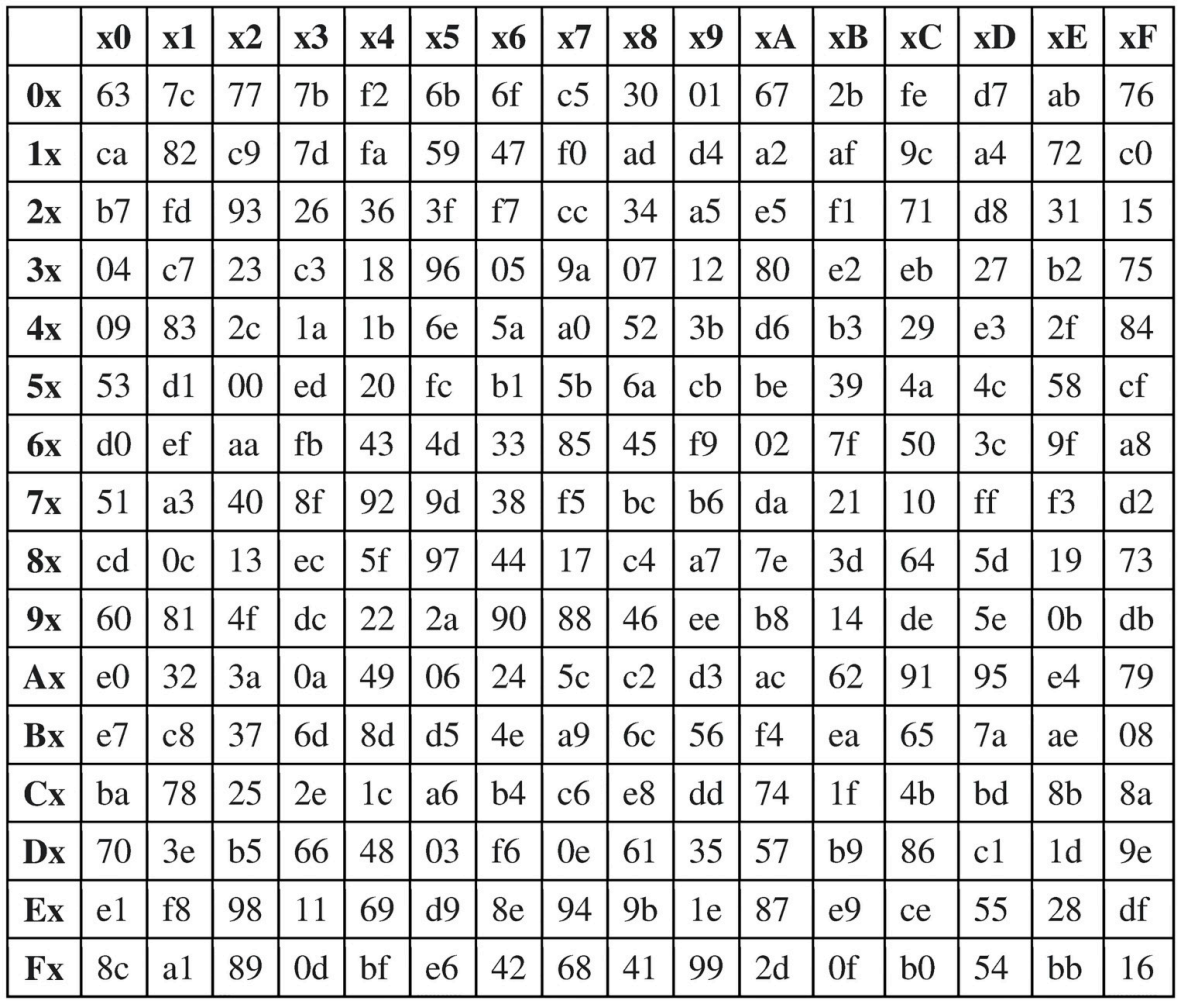
The S-BOX.

The substitution scheme is a very essential step to remove the traces of the original input block from the resulting block. It, therefore, weakens the correlation between the plaintext and its respective ciphertext. To perform its functionality, the substitution method adopts the same substitution space used by AES (Advanced Encryption Standard) technique. The substitution space, S-BOX, is a 16 × 16 table that includes all the possible permutations of the byte (See Fig 7). The entries of S-BOX are organized exactly as suggested by AES because such an organization makes the correlation between the input of the substitution method and its output substantially insignificant.

Given the substitution space S-BOX, substituting any symbol *a*_*i*_ is straightforward. The substitution method maps the input symbol *a*_*i*_ to the S-BOX by splitting the bits of *a*_*i*_ into two halves: the left half bits and the right half bits index respectively the rows and the columns of the substitution table. (Note in Fig 7, the left half and the right half bits are 4 bits each.) The value in the indexed entry is the mapping outcome, which is the substitution for *a*_*i*_. For example, to substitute the input symbol “y” (“01111001”) using the substitution table in Fig 7, the left four bits “0111” (7 in Hex) index the rows of the substitution table and the right four bits “1001” (9 in Hex) index its columns. The value “b6” is the substitution for the input symbol “y”.

**Fig 8:**
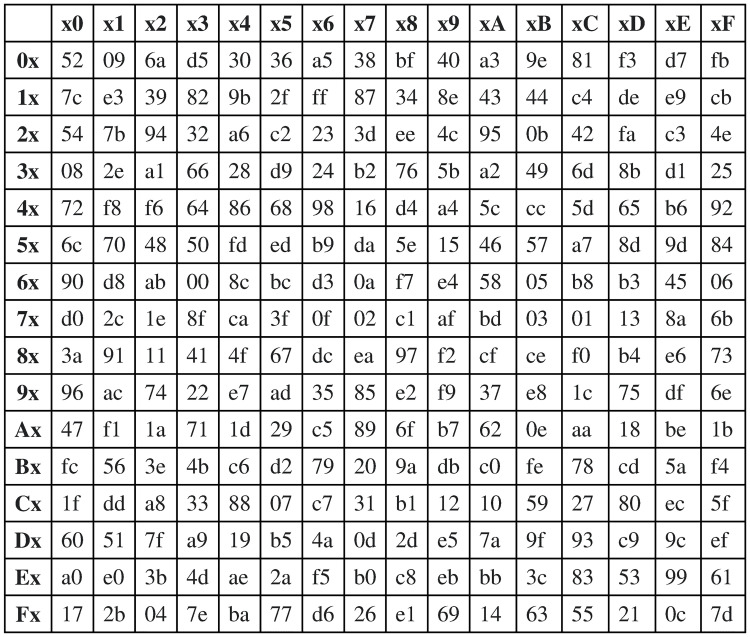
The S-BOX inverse.

We can reverse the impact of the symbol substitution method and recover the original block easily. The algorithmic steps for recovering the original block are identical to that of the symbol substitution method except that we use the inverse of the S-BOX (instead of S-BOX). Fig 8 shows the S-Box Inverse for S-Box in Fig 7. For instance, recall that we substituted the symbol “y” with the symbol “b6”. To restore the original symbol “y” from “b6”, we substitute “b6” using the S-Box Inverse. The Hex “b” (of “b6”) indexes the rows and the Hex “6” indexes the columns, resulting in the value “79”, which the Hex value of the original symbol “y”.

### Dual diffusion method

**Fig 9:**
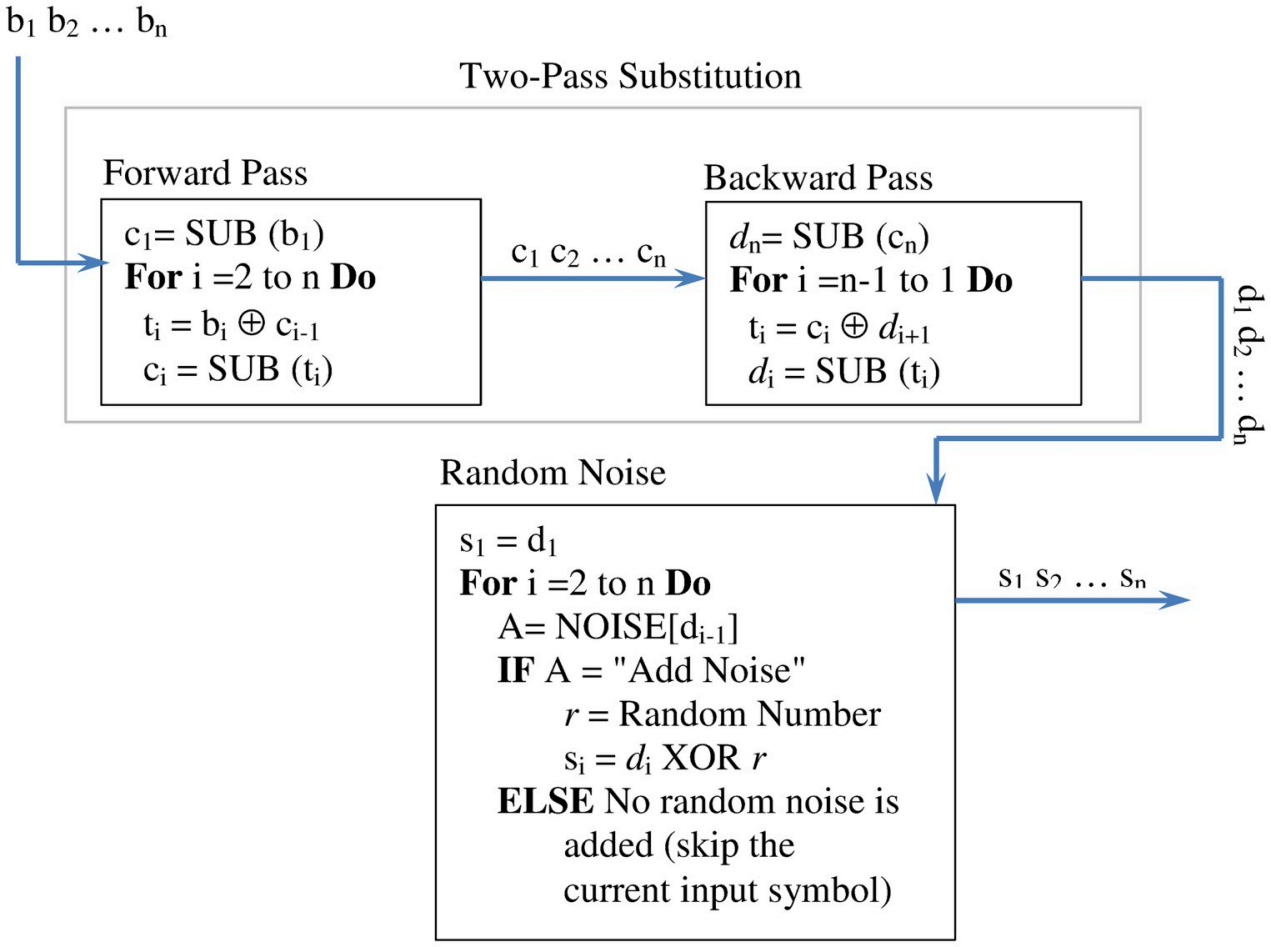
The dual diffusion method: The processing instructions and flow of control.

The dual diffusion method is the major source of confusion in the proposed encryption technique. It takes a block of symbols *b*_1_*b*_2_…*b*_*n*_ and processes them so that every bit in the input affects every bit in the output. The processing of the dual diffusion method detects any bit-change in the input, magnifies this change, and spreads its impact to each symbol in the output block. Out dual diffusion method achieves this by dual scanning of the block’s symbols along with a random nosing (see Fig 9). The dual symbol scanning is performed by dual-pass substitution operation: Forward pass and Backward pass.

The forward pass reads the symbols of its input left-to-right and processes each symbol through symbol substitution and XOR operations. The first symbol *b*_1_ is substituted using the operation SUB(*b*_1_), which splits *b*_1_ bits into two halves where the left half indexes the row of the S-Box and the right half indexes the columns (exactly as described in previous Subsections). For the remaining symbols *b*_*i*_(*i* > 1), the forward pass uses the outcome of substituting the previous symbol *b*_*i*−1_ to impact the substitution of the current symbol *b*_*i*_. Therefore, substituting the symbols *b*_*i*_(*i* > 1) is performed by XORing the input symbol *b*_*i*_ with the outcome of substituting the previous symbol *b*_*i*−1_ (i.e. with *c*_*i*−1_), and substituting the outcome of the XOR operation, say *t*_*i*_. The result of the substitution is the symbol *c*_*i*_, which is the substitute for the input symbol *b*_*i*_. Note that the forward pass spreads the impact of the symbols from left to right. If an input symbol *b*_*i*_ changes, the substitution for all the subsequent input symbols *b*_*j*_(*j* > *i*) will be impacted as well.

The backward pass reads the symbols of its input (the output of the forward pass) from right-to-left. It functions like the forward pass except that it processes the input backward. As such, the backward pass substitutes the symbol *c*_*n*_ (the rightmost symbol in the input) in the usual way to yield the output symbol *d*_*n*_. For the remaining symbols *c*_*i*_(*i* < *n*), the backward pass performs an XOR operation between the input symbol *c*_*i*_ and the outcome *d*_*i*+1_ of the lastly performed substitution to yield the output symbol *t*_*i*_. The symbol *t*_*i*_ is substituted and the outcome *d*_*i*_ is the substitute for *c*_*i*_. It is clear that the backward pass deepens the mutual impact of symbols by spreading this impact from right to left. The change to the symbol *c*_*i*_ will propagate back to affect all predecessor symbols *c*_*j*_(*j* < *i*).

Random Noise operation adds further confusion to the output block of the two-pass substitution. The functionality of the random noise operation is fully controlled by a probabilistic model. That is, nosing an input symbol depends on some computed probability. To determine which input symbol must be randomly noised, the random noising operation maintains a list NOISE of 2^*L*^ entries, where *L* is the number of bits that represent that used symbols. For instance, if we only deal with the symbols from 0 to 255, *L* = 8. This list is filled with *P* occurrences of the token “Add Noise”, where *P* < = 2^*L*^. The reset of the entries are filled with the token “NULL” (no random noising). The entries of the list NOISE are randomly scattered using a list of 2^*L*^ random numbers. (The random generator provides this list of random values.) The ratio *P*/2^*L*^ is the intensity of random noising. As *P* increases so does the number of symbols that will be noised. of course, small *P* causes fewer input symbols be noised.

The random noising operation uses the list NOISE and manipulates the symbol as follows. Suppose the input block is *d*_1_*d*_2_…*d*_*n*_. The first input symbol *d*_1_ will not be processed (is not noise). The remaining input symbols *d*_*i*_(*i* > 1) receive random noising based on their predecessors *d*_*i*−1_ and on the state of the NOISE (the order of the elements). As shown in Fig 9, the predecessor symbol *d*_*i*−1_ indexes the list of tokens NOISE. If the outcome of the indexing is the token “NULL”, the input symbol *d*_*i*_ is passed on to the output channel without processing. If the outcome of the indexing is the token “Add Noise”, the symbol *d*_*i*_ receives random noising as follows. The random noise operation will obtain a random number from the random generator and XORes the random number with the symbol *d*_*i*_ to yield the noised symbol *s*_*i*_. Note that whether an input symbol *d*_*i*_ will be noised or not cannot be determined ahead of time; the noising is fully controlled by the state of the list NOISE (Random ordering) and the lookback symbol and the intensity of the random noising.

Before concluding this section, we reemphasize that the proposed diffusion method is very sensitive to the change in the input regardless of the change’s position in the block and its magnitude (one bit or more). The two-pass substitution ensures spreading the change to all of the bits that follow the changed bit (forward pass) and that precede the changed bit (backward pass). The random noising changes the structure of the output by introducing noise to some of its symbols, thereby eliminating the traces that may help in predicting the original input block.

#### Dual diffusion inverse method

**Fig 10:**
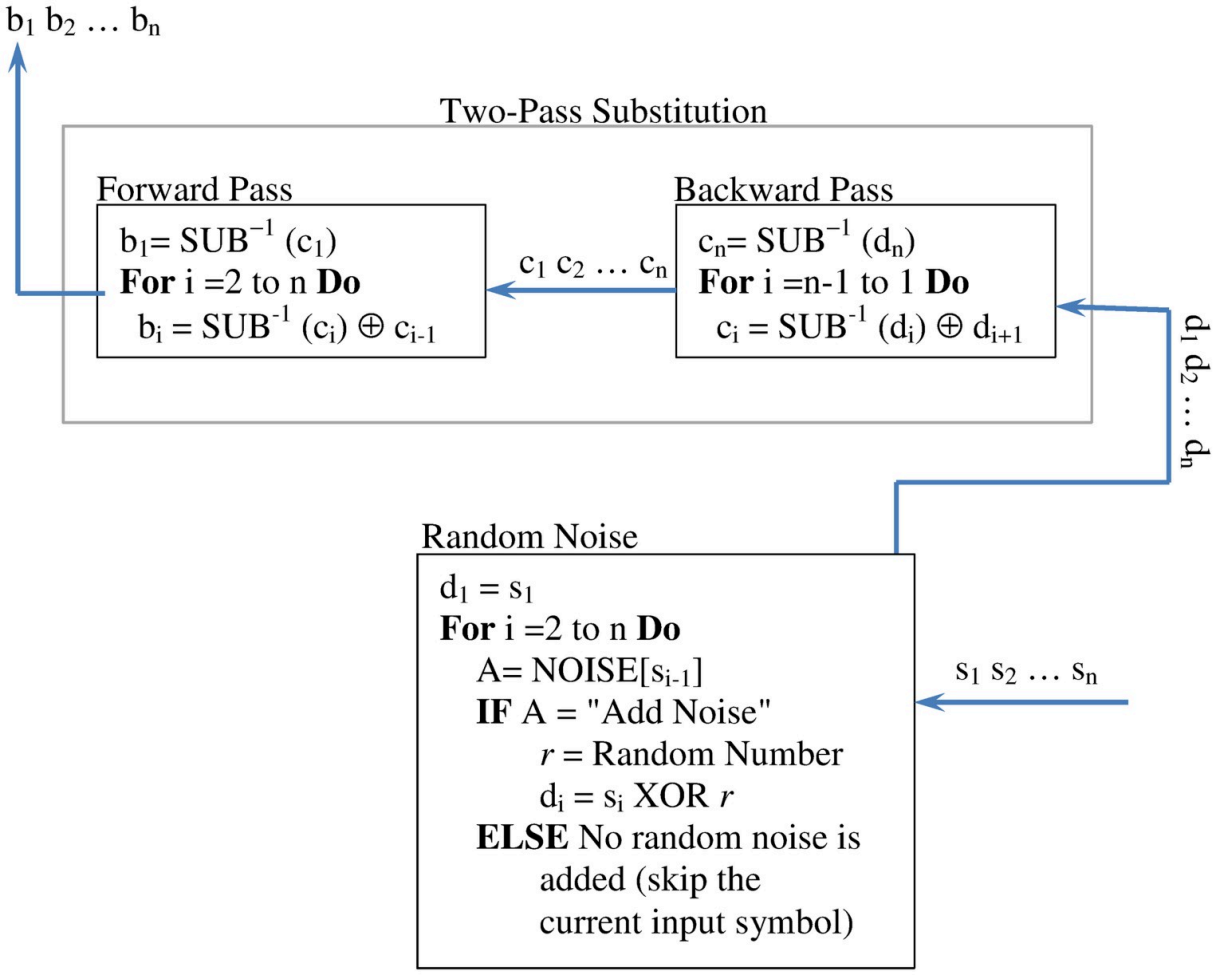
The dual diffusion inverse method: The processing instructions and the flow of control.

This method cancels the impact of the dual diffusion method and restores the original block. It is, therefore, used during the decryption to recover the plaintext. The logic of the diffusion inverse method is outlined in Fig 10. It executes roughly the same steps as the dual diffusion. The principal difference is that the inverse diffusion processes the input backward and with a slight modification to the substitution action. Therefore, to restore the original block *d*_1_*d*_2_…*d*_*n*_ from the diffused block *s*_1_*s*_2_…*s*_*n*_, the inverse diffusion executes first the Random Noise action to remove the embedded random noises. The backward pass processes its input block *d*_1_*d*_2_…*d*_*n*_ using substitution and XOR operations. It recovers the symbol *c*_*n*_ by substituting the input symbol *d*_*n*_ using the substitution inverse operation SUB^−1^(*c*_*n*_). (The substitution inverse operation SUB^−1^(*c*_*n*_) uses the S-Box Inverse not SBox). The remaining symbols *c*_*i*_(*i* = *n* − 1, *n* − 2…1) are restored as shown Fig 10. Any symbol *ci* is recovered by substituting the input symbol *d*_*i*_ using the operation SUB^−1^(*d*_*i*_) and then XORing the outcome of the substitution with the input symbol *d*_*i*+1_. Finally, the forward pass processes the output of the backward pass (*c*_1_*c*_2_…*c*_*n*_) and recover the original block *b*_1_*b*_2_…*b*_*n*_. The symbol *b*_1_ is restored by substituting *c*_1_ using the substitution inverse operation SUB^−1^(*c*_1_). The remaining symbols *b*_*i*_’s are recovered by substituting the input symbol *c*_*i*_ using the operation SUB^−1^(*c*_*i*_) and then XORing the outcome of the substitution with the input symbol *c*_*i*+1_.

### Block substitution method

The block substitution method replaces the symbols of its input block with new ones. This replacement aims at greatly reducing the intrinsic correlation between the symbols of the input block. The block substitution method uses the symbol substitution method (described before) along with supporting tables to accomplish its task. The first table DIR-TAB is 4 × 4 whose entries are directive flags that instruct the substitution method to move along a specific direction within the substitution table (S-BOX). The directive flags are four descriptors: U (move Up), D (move Down), L (move Left), and R (move Right). Since the table DIR-TAB has 16 entries but there is only four descriptors, these four descriptors are replicated in the four rows.

The second table MOV-TAB is also 4 × 4 with its cells (16 cells) include the integers [0…15]. Each cell has distinct integer. The integers *x*_*i*_ ∈ [0…15] represent the amounts of the possible moves within the substitution table. The entries of the tables DIR-TAB and MOV-TAB are randomly reordered to maximize the confusion of the substitution method. The reordering is performed by two different sequences of random numbers obtained from the random generator. Reordering the entries of DIR-TAB (or MOV-TAB) is done straightforwardly by swapping the entry at index (*i*) with the entry at index (*r*_*i*_), and *r*_*i*_ is a random integer.

The third table *F*-TAB is a 4 × 4, which contains 14 different bitwise-distortion actions. Table 2 lists these actions along with a brief description of their functionality. Without losing the generality, it is assumed that each symbol is 8 bits. (This assumption is only for simplifying the presentation and imposes no restriction on the generality of the method.) These 14 actions are organized in *F*-TAB. Because *F*-TAB consists of 16 entries and there are 14 bitwise-distortion actions, the remaining two entries are filled with a dummy action that performs no processing (null action). The entries of *F*-TAB are randomly scattered using 16 random numbers provided by the random generator.

**Table 2:** The *F*-TAB: 14 bitwise-distortion actions.

Flipping actions	Functionality
*F*^*u*^ (u = 1…8)	mutates the *u*^*th*^ bit of the input symbol (e.g. *F*^5^ mutates the fifth bit of the input symbol).
*F* ^*L*/2^	mutates the left half bits of the input symbol.
*F* ^*R*/2^	mutates the right half bits of the input symbol.
*F* ^*L*/4^	mutates the left quarter bits of the input symbol.
*F* ^*S*/4^	mutates the second quarter bits of the input symbol.
*F* ^*T*/4^	mutates the third quarter bits of the input symbol.
*F* ^*R*/4^	mutates the rightmost quarter bits of the input symbol.

The block substitution uses the input symbols along with random noises to perform highly illusive replacement to the symbols of the inputs. Suppose we have the input block *x*_1_*x*_2_…*x*_*n*_ with each symbol *x*_*i*_ is represented by 8 bits. (Although, we assume that each symbol is 8 bits, the substitution method is general and is independent of the number of bits that represents a symbol.) The bits of each symbol *x*_*i*_ are divided to two halves, where the left half *l* and right half *k* create an initial reference (*l*, *k*) within the substitution space. The reference (*l*, *k*) is randomly distorted by shooting it to a new random position within the substitution space. To randomly shoot the reference (*l*, *k*) within the substitution space, a random number *r*_*i*_ is requested from the random generator. The left four bits of *r*_*i*_ are used to retrieve a direction flag *M* from the move direction flags table (DIR-TAB). Retrieving *M* is done by using the left two bits to index one of the rows (of DIR-TAB) and the right two bits to index one of its columns. The right four bits of the random number *r*_*i*_ are used to retrieve a move amount *V* from the table MOV-TAB. Retrieving *V* from MOV-TAB is performed exactly as we did for retrieving a direction flag from DIR-TAB. Therefore, the block substitution method shoots the initial reference (*l*, *k*) a number of positions equals the amount of the move *V* along the direction specified by the direction flag *M*. The symbol *y*_*i*_ at the new reference (*u*, *t*) is retrieved as a replacement for the original input symbol *x*_*i*_.

The output of the substitution method *y*_1_*y*_2_…*y*_*n*_ is further distorted by applying the bitwise-distortion actions. Suppose that *w*_1_*w*_2_…*w*_*n*_ is a sequence of random numbers. The middle-half bits of the random number *w*_*i*_ is used to access one of the bitwise-distortion actions of *F*-TAB. For instance, if the random number *w*_*i*_ is “01110010”, the middle-half bits “1100” accesses one of the bitwise-distortion actions. The input symbol *y*_*i*_ is distorted using the retrieved the distortion action (if it is not dummy). The outcome of processing the symbol *y*_*i*_ is the new symbol *z*_*i*_.

Before concluding this section, we emphasize the substitution method is highly non-linear. This nonlinearity can be largely attributed to the random jumping within the S-BOX (i.e. adding random effects to the substitution). In particular, the substitution relies not only on the symbol to be substituted, but also on the random effects produced by the random number generator. Additionally, the bitwise-distortion actions guarantee a maximum level of confusion because their impact is random.

### Key round

The key round is the closing stage in which the symbols of the ciphertext (the output of the distortion layer) is additionally secured by embedding these symbols with codes generated using the secret key. The encryption key must, therefore, be expanded to match the length of the output (the ciphertext). One of the effective and efficient secret key expansion techniques is proposed in [53]. This expansion technique has three important properties that make it ideal for our approach. First, it is very sensitive to the key changes; it can detect minor changes to the key and react by producing very different key sequences. Second, it can expand the key to any arbitrary length without ever showing patterns in the generated sequence. Third, the generated key sequences are very random.

**Fig 11:**
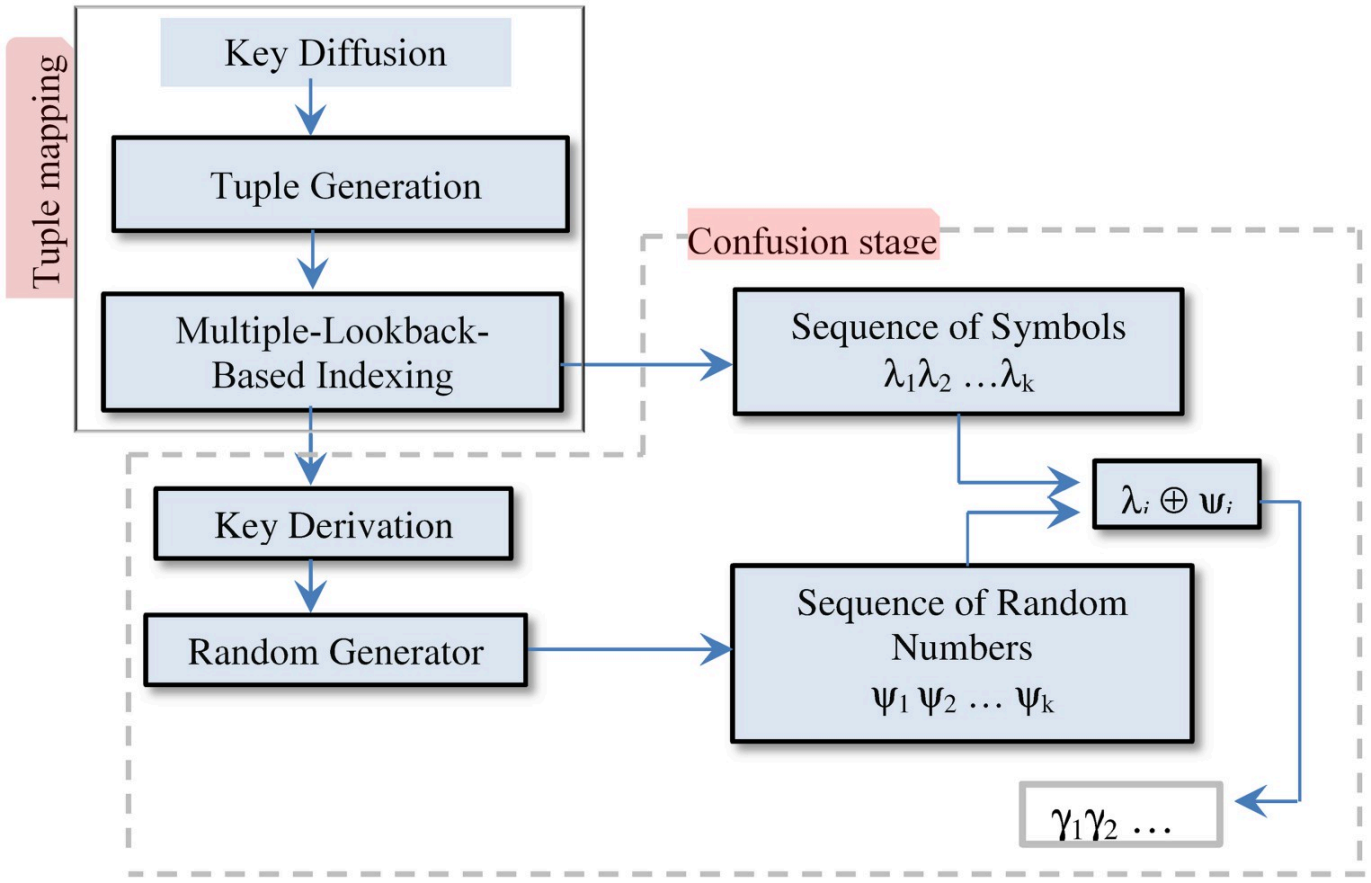
The key expansion process reproduced from [53].

For the sake of self-containment, we provide a succinct description of the key expansion technique and refer the interested readers to [53] for more technical details. Fig 11 outlines the control flow of the key expansion technique. The technique is founded on two primary processing stages: the tuple mapping stage (or expansion stage) and the confusion stage.

The tuple mapping stage extends the secret key. It uses three operations for the key extension. The **Key Diffusion** operation is extremely important. It detects any changes that occur to the secret key and spread these changes affect every symbol in the output (i.e. if one bit changed in the key, the resulting output will be drastically impacted). The **Tuple Generation** operation creates all possible *n*-place tuples from the diffused key symbols (processed by the diffusion operation), where symbol-duplicate is allowed and the order of the symbols in the tuple matters. For instance, if the key is “ABC”, the 2-place tuples include “AA”, “AB”, “BA”, and so on. The size of the tuples *n* depends on the number of layers in the indexing technique (Multiple-Lookback-Based Indexing). After generating all the possible tuples, the initial key sequence is built by mapping all the tuples using the **Multilayered-Lookback-Based Indexing** operation. This indexing technique maps each *n*-place tuple $T^{i}<a_{1}^{i},a_{2}^{i},...,a_{n}^{i}>$ into a single symbol *b*_*i*_. The outcome of tuple mapping is the initial key sequence λ_1_, λ_2_…λ_*k*_.

The Confusion stage (the second stage) imposes further distortion on the initial sequence. This added distortion is accomplished by mixing the initial key sequence with random numbers. To generate these random numbers, *L* symbols are extracted from the leftmost of the initial key sequence (λ_1_, λ_2_…λ_*k*_) and used as a new key (*L* equals the size of the original key). This derived key is then fed as a seed to the random generator, which creates a stream of *k* random numbers *ψ*_1_*ψ*_2_…*ψ*_*k*_. The final key sequence is then generated by XORing each initial key symbol λ_*i*_ with the corresponding random number *ψ*_*i*_ to yield the symbol *γ*_*i*_.

Once the key stream *γ*_1_*γ*_2_…*γ*_*k*_ is fully generated, we mix the symbols of the key stream with the ciphertext symbols using an XOR operation. That is, the final ciphertext symbols is calculated by *γ*_*i*_⊕*c*_*i*_, where *c*_*i*_ is a symbol of the ciphertext. Note, due to the fact that the key expansion process generates highly random numbers, the key stream *γ*_1_*γ*_2_…*γ*_*k*_ ensures maximum protection to the ciphertext against hacking techniques.

## Distortion inverse layer

The distortion inverse layer decrypts the ciphertext. Successful decryption requires the use of the same secrete key. Therefore, to recover the plaintext from the ciphertext, the secret key is used as a seed for the random generator to produce stream of random numbers that are identical to the stream used during the distortion. Once this stream is prepared, we can configure the tables DIR-TAB, MOV-TAB, and *F*-TAB to the same state that is used during the distortion. That is, the entries of these three tables are randomly reordered using the same random numbers that were used to reorder them during the distortion.

When these three tables are fully initialized, the decryption process starts by performing the Key Round. The Key Round uses the secret key to produce a stream of key symbols. This stream of symbols is necessarily identical to the stream that was produced during the encryption (they are produced using the same secret key). The generated stream is used to remove the impact of the key from the ciphertext by performing an XOR operation between the key stream symbols and the respective ciphertext symbols.

Once the key impact is removed from the ciphertext, the block substitution method processes the symbols of the resulting ciphertext as follows. Let *y*_*i*_ be a ciphertext symbol and *r*_*i*_ be a random number. The middle half bits of *r*_*i*_ creates an index to access *F*-TAB table and retrieve a bitwise-distortion action. Note, the random number *r*_*i*_ is identical to the one, which was used to access the *F*-TAB when encrypting the symbol *y*_*i*_, and therefore the retrieved bitwise-distortion action is necessarily the same as the one used to encrypt *y*_*i*_. The retrieved bitwise-distortion action processes the input symbol *y*_*i*_ and restores the distorted bits of *y*_*i*_ yielding the new symbol *z*_*i*_. The symbol *z*_*i*_ is substituted using S-BOX as follows. The left half bits of the random number *r*_*i*_ creates an index to access DIR-TAB table and retrieve a direction flag *M* (could be any of the direction flags: U, D, L, R). The right half bits of *r*_*i*_ creates an index to access MOV-TAB table and retrieve an amount of move *V*. The symbol *z*_*i*_ is then looked up from S-BOX and the index (*i*, *j*) at which *z*_*i*_ was located is used to retrieve the original symbol. To retrieve the original input symbol, the retrieved direction flag and the amount of move (*M*, *V*) are used. However, instead of using the direction flag itself, we use its inverse. Therefore, if the retrieved direction flag is U (Up), the reverse direction flag D (Down) is used. Using the proper direction flag and the amount of move, the substitution method moves down the current index (*i*, *j*) a number of positions equal to the amount of move to reach the new index (*l*, *k*). The original symbol is recovered by concatenating the bits of *l* followed by bits of *k* (i.e. *lk*) and then find the corresponding decimal.

The final step is to redo the impact of the dual-diffusion method. The inverse distortion layer applies the inverse dual-diffusion method to redo the impact of the dual-diffusion and therefore restore the plaintext block.

## Experimentation and performance analysis

The performance of the encryption technique is evaluated based on the guidelines established by the National Institute for Standards and Technology (NIST). According to these guidelines, the effectiveness of the encryption technique is measured using a set of randomness tests. The encryption technique is effective if its output (ciphertext) passes fundamental randomness tests. (Testing the random generator for randomness is beyond the scope of this paper and was done elsewhere [8]).

But before we present the evaluation results for the proposed encryption technique, we report our simulation results for the dual diffusion method. As discussed before, the dual diffusion method is the major source of confusion. We, therefore, ran simulations to assess it effectiveness. We measure the effectiveness by the response of the method to the changes of the input (or the avalanche effect). For this purpose, we started with a sequence of 1024 bits (128 bytes). We created 20 sets of sequences, each set contains 256 sequences. These sequences were created by flipping *r* bits in different/randomly selected positions of the original input (*r* = 1..16, 32, 64, 128, 256). For instance, the first set contains 256 sequences, where each sequence was created by flipping one bit of the original input, the second set is created by flipping 2 bits of the original sequence, and so on. We then processed the original input and each of the created sequences using the dual diffusion method and counted the number of bits difference between the result of processing the original input and the result of processing each created sequence. Table 3 shows the performance in terms of minimum and average number of bit difference between the result of processing the original input sequence and the created sequences. Generally, regardless of how many bits were changed (flipped), this change to the input causes on average more than half of the bits to change. This indicates that the dual diffusion method has a high avalanche effect.

**Table 3:** Avalanche effect of dual diffusion method.

Flipped bits	Min	Average	Flipped bits	Min	Average
1	489	524	11	501	577
2	499	548	12	492	539
3	501	536	13	519	544
4	530	561	14	525	552
5	513	547	15	517	541
6	498	528	16	492	539
7	510	543	32	522	561
8	518	548	64	532	567
9	522	561	128	524	563
10	524	558	256	523	564

### Randomness tests

We define the hypotheses the we want to test.

*H_0_ (Null): the ciphertext does not deviate from randomness*.

*H_1_ (Alternative): the ciphertext does deviate from randomness (i.e. the output is not random)*.

The decision whether to accept *H*_0_ or reject it (and therefore accept *H*_1_) depends on a value, called (**p-value**), which is computed by the randomness test. The **p-value** is compared to a prespecified significance level (*α*)(*α* can be any value within the interval (0, 1]. Common choices include: 0.001, 0.01, and 0.05). The hypothesis *H*_0_ is accepted only if **p-value** ≥ *α*. In this case we conclude that the tested ciphertext is random. If **p-value** < *α* we reject *H*_0_ (accept *H*_1_) and conclude that the tested ciphertext is not random.

The most effective randomness tests for encryption techniques are those created by the National Institute for Standards and Technology—NIST [54, 55]. We chose the following randomness tests due to their high sensitivity in detecting deviations from randomness. All the definitions are taken from [55].

- **Runs test**: matches the runs of ones and zeros of various lengths with the ones expected for a random sequence.
- **Frequency test (Monobit)**: examines if the number of ones and zeros that appear in the tested ciphertext are approximately the same as expected for a truly random sequence.
- **Discrete fourier transform test (Spectral)**: detects repetitive patterns that are near each other in the analyzed ciphertext that would present a divergence from the assumption of randomness.
- **Serial test**: checks if the number of occurrences of the 2*m* *m*–bit overlapping patterns is approximately the same as would be expected for a random sequence. Random sequences have uniformity in a sense that each m–bit pattern has an equal chance of appearing as every other *m*–bit pattern.
- **Cumulative sums test**: determines if the cumulative sum of the partial sequences occurring in the tested sequence is too large or too small relative to the expected behavior of that cumulative sum for random sequences. The cumulative sums may be considered as random walks. If the sequence is random, the excursions of the random walk should be near zero.
- **Linear complexity test**: determines whether or not the sequence is complex enough to be considered random.
- **Binary matrix rank test**: checks for linear dependence among fixed length substrings of the original sequence.
- **Approximate entropy test**: compares the frequency of overlapping blocks of two consecutive/adjacent lengths (m and m + 1) against the expected result for a random sequence

We further evaluated the encryption technique using ENT randomness test battery [56]. This battery includes the following important randomness tests.

- **Entropy**: determines information density of the contents of the file, expressed as a number of bits per character.
- **Chi-square Test**: determines whether a stream of bytes is random.
- **Arithmetic Mean**: computes the average of bytes. If average is close to the middle value of the range of the used values, the sequence is potentially random.
- **Monte Carlo Value for Pi**: if the tested sequence random, the Monte Carlo value would be close to the true value of Pi.
- **Serial Correlation Coefficient**: measures the extent to which each byte in the file depends upon the previous byte.

### Security analysis

We define the data sets that we used to test our technique, and then present the results of the randomness tests. We prepared the data sets as recommended elsewhere [54, 55]. To unify the testing data, we use–without the loss of generality–the Unicode symbols within the range from 0 to 255. This restriction allows us to represent each symbol in the ciphertext (the output of the encryption technique) using 8 bits. To thoroughly analyze the randomness properties of the encryption technique, we performed the testing using the following data sets.

1. **Key avalanche data set**. This data set measures how the proposed encryption method responds to changes of the encryption key. Effective encryption technique must produce random ciphertext regardless of the keys’s change (whether the change is major or minor).
2. **Plaintext avalanche data set**. This data set measures how the proposed encryption method responds to changes of the plaintext. It is well known that effective encryption techniques must produce random ciphertext independent of the magnitude of the change (major or minor).
3. **Plaintext/Ciphertext correlation data set**. This data set measures the correlation between plaintext/ciphertext pairs. The presence of the correlation indicates inherited patterns from the plaintext to its corresponding ciphertext; a very critical security problem. Thus, effective encryption techniques must greatly remove the correlation between the plaintext and its respective ciphertext.

Firstly, to measure how sensitive the proposed encryption method to the changes of the encryption key, we created and analyzed 300 sequences of size 65,536 bits each. We fixed the plaintext input to the technique by using a 512-bit (64 bytes) plaintext of all zeros. We used 300 keys each of size 128 bits. These keys are derived from the weights generated by the neural network layer. Each of the 300 sequences was built by concatenating 128 derived blocks created as follows. Each derived block is constructed by XORing the ciphertext created using the fixed plaintext and the 128-bit key with the ciphertext created using the fixed plaintext and the altered 128-bit key with the *i*^*th*^ bit is modified, for 1 ≤ *i* ≤ 128.

Secondly, to measure the sensitivity of the proposed technique to changes of the plaintext, we created and analyzed 300 sequences of size 65,536 bits each. We derived 300 plaintexts of size 512 bits (64 bytes) from the output of the neural network. We also fixed the key to 128 bit of all zeros to neutralize its impact on the output of the technique and hence study the pure effect of the plaintext change. Each sequence was created by concatenating 128 derived blocks constructed as follows. Each derived block is created by XORing the ciphertext created using the 128-bit key and the 512-bit plaintext with the ciphertext created using the 128-bit key and the altered 512-bit plaintext with the *i*^*th*^ bit changed, for 1 ≤ *i* ≤ 512.

Thirdly, to analyze the correlation of plaintext with its respective ciphertext, we constructed 300 sequences of size 358,400 bits per a sequence. Each sequence is created as follows. Given a 128-bit key and 700 random plaintext blocks (the size of each block is 512 bits), a binary sequence was constructed by concatenating 300 derived blocks. A derived block is created by XORing the plaintext block and its corresponding ciphertext block. Using the 300 (previously selected) plaintext blocks, the process is repeated 299 times; one time for every additional 128-bit key.

Tables 4–6 show the results of the randomness tests. The tables present the randomness tests, the number of sequences that passed the corresponding randomness test (Success), the number of sequences that failed the corresponding randomness test (Failure), and the Success rate. We fixed the significance level to the value 0.05 for all the randomness tests. When we fix the significance level to 0.05, we actually imply that, under ideal experimenting settings, no more than 5 out of 100 binary sequences may fail the corresponding test. However as stated in [54], in all likelihood, any given data set will deviate from this ideal case. Therefore, for a more realistic interpretation, we use a 95% confidence interval (CI) for the proportion of the binary sequences that may fail a randomness test at significant level of 0.05. (The maximum number of binary sequences that are expected to fail at the level of significance *α* is computed using the following formula [55]: $S(\alpha +3\sqrt{\frac{\alpha (1-\alpha }{S}})$, where *S* is the total number of sequences and *α* is the level of significance).

**Table 4:** Key avalanche test.

Randomness Test	Success	Failure	Success rate	CI
Runs test	292	8	97.33%	26.32
Monobit test	294	6	98.00%	26.32
Spectral test	259	41	86.33%	26.32
Serial test	276	24	92.00%	26.32
Cumulative sums test	281	19	93.70%	26.32
Linear complexity test	289	11	96.30%	26.32
Binary matrix rank test	291	9	97.00%	26.32
Approximate entropy test	288	12	96.00%	26.32

**Table 5:** Plaintext avalanche test.

Randomness Test	Success	Failure	Success rate	CI
Runs test	296	4	98.67%	26.32
Monobit test	293	7	97.67%	26.32
Spectral test	274	26	91.33%	26.32
Serial test	289	11	96.33%	26.32
Cumulative sums test	287	13	95.70%	26.32
Linear complexity test	292	8	97.33%	26.32
Binary matrix rank test	292	8	97.33%	26.32
Approximate entropy test	283	17	94.30%	26.32

**Table 6:** Plaintext/Ciphertext correlation test.

Randomness Test	Success	Failure	Success rate	CI
Runs test	295	5	98.33%	26.32
Monobit test	298	2	99.33%	26.32
Spectral test	281	19	93.67%	26.32
Serial test	295	5	98.33%	26.32
Cumulative sums test	288	12	96.00%	26.32
Linear complexity test	285	15	95.00%	26.32
Binary matrix rank test	287	13	95.67%	26.32
Approximate entropy test	292	8	97.30%	26.32

Referring to the tables, the success rate indicates that the proposed technique is effective. The rate of success for all the randomness tests is remarkably high. The number of sequences (ciphertexts) that failed any test is less than the maximum expected number of sequences that could fail under the fixed significance level (0.05). The number of sequences that failed the Spectral test deviates from this nice pattern. In fact, the number of sequences that failed the Spectral test is greater than the maximum expected in only Table 4. (Note in Table 4, the number of failed sequences is 41 while the maximum expected under the significant level of 0.05 is 26.32).

Table 7 shows the results of ENT randomness tests. The columns *Tp*, *Tk*, and *Tc* show respectively the Plaintext avalanche, Key avalanche, and Plaintext/Ciphertext correlation. Based on the ENT test interpretation [56], the test numbers show that the output of the encryption technique (ciphertext) does not deviate from randomness. For instance, the arithmetic mean is pretty close to 0.5 and chi-square is within the ranges that indicate randomness. Additionally, the entropy for the bit strings is close to 1 (1 is the perfect value).

**Table 7:** Result of ENT test: Plaintext avalanche (Tp), Key avalanche (Tk), and Plaintext/Ciphertext correlation (Tc).

Randomness Test	Tp	Tk	Tc
Entropy	0.9998311	0.9986109	0.9991818
Chi-square Test	56.79%	54.01%	0.57.12%
Arithmetic Mean	0.4951940	0.4896016	0.4990013
Monte Carlo Value for Pi	3.1321678	3.110752	3.1343081
Serial Correlation Coefficient	0.0081	−0.0023	0.0102

We attribute this remarkably high performance to the synergistic collaboration between the neural network layer and the distortion layer. The neural network provides good keys that are effectively exploited by the distortion layer. The distortion layer is the major source of confusion: it stimulates a great amount of randomness in the ciphertext. The high success rate indicates really high performance of the distortion layer and the hybrid encryption technique.

We conclude this section by pointing out why the proposed technique is worth considering. The technique has indeed many important features that distinguish it from other proposed techniques such as *AES* and *DES*. First, while other techniques focus on only confidentiality, the proposed technique addresses, in one coherent system, the two fundamental aspects of information security: confidentiality and integrity. As described in the manuscript, the encryption technique encrypts the data (ensures confidentiality) and also produces hash code to ensure integrity. The computation of the hash code is done in a piggyback manner in a sense it is performed in parallel with the encryption. (Therefore, the computation of the hash code does not incur additional processing time). Second, the proposed technique has effective distortion operations that produce deep confusion. The distortion operations are designed to be computationally simple: mostly operate at the bit level and can be done easily on the hardware. Besides, the number random generator works synergistically with the distortion operations to produce higher confusion necessary for immunizing the technique against hacking tools. Third, while other encryption techniques (most notably *AES* and *DES*) rely on simple methods to expand the key and add its impact through an XOR operation, our technique relies on a more robust mechanism to expand the key and hide its trace (hiding the trace is ensured by the operations that expand the key). As discussed in the “Key round” section, the proposed technique expands the key to any arbitrary length to match the ciphertext length. This means that our method adds the impact of a different sequence of the expanded key to each block. This is at odds with the *AES*, for instance, where the key is expanded to match the block size times the number of rounds. This means that all the blocks receive the same impact of the key. Fourth, the number of required rounds to achieve acceptable randomness is a function of the key length in most of the encryption techniques (e.g. *AES* and *DES* encryption technique). This means that longer keys require more rounds, which means more processing time. Our technique simply does not depend on the key length due to the way in which the key is expanded.

### Attack resistance

One of the uncompromisable properties of encryption techniques is their ability to resist attacks. Differential attacks are one the most effective threats to the encryption technique. These attacks exploit the patterns that appear in the ciphertext due to the weak confusion. The major source of confusion of the proposed techniques is the distortion process. The distortion process has two effective confusion-boosting subprocesses. The substitution subprocess, which provides a nonlinear transformation of the plaintext [15]. The dual diffusion subprocess imposes deep bit mixing, which in turn ensures that any bit change in the input causes significant bit change in the output (please see Table 3). These two subprocesses work synergistically to remove any patterns that may help predict the encryption key.

Another significant threat is called classic attacks [34]. Four classic types of attacks could challenge encryption techniques. These types are (1) ciphertext-only attacks, (2) known-plaintext attacks, (3) chosen-plaintext attacks, and (4) chosen-ciphertext attacks. Based on [33], the chosen-plaintext attack mode is known to be the most effective one. As such, and pointed out in [33], if the encryption technique can resist chosen-plaintext attacks, it can resist all of the other three attack modes. The proposed technique can resist chosen-plaintext attacks. First, the distortion process directly uses plaintext information for diffusing any change to the plaintext symbols and propagate this change to impact all other symbols. Second, the random number generator provides noises (random numbers) that are embedded in the output, causing this output to vary based on the key. Third, the neural network ensures deep and nonlinear transformation to the output. All these three make the relationship between the plaintext and ciphertext highly complicated, which ensure resistance against chosen-plaintext attacks.

### Technique efficiency

We compare the efficiency of the proposed technique with the efficiency of state-of-the-art techniques. Table 8 shows the encryption techniques and the execution time (in milliseconds) for different input sizes. The implementation was in JAVA and the execution hardware is Intel core i5 processor with 4GB memory and windows 10 operating system. As the time numbers show, the execution time of the AES technique tends to be better than the proposed technique for small inputs. However, for inputs of 250KB and larger, the proposed technique appears to have a shorter execution time. This lower execution time can be attributed to the efficiency of the operations especially the distortion operations (they are very light-weighted operations). Except for the AES, the other techniques required more time for encryption.

**Table 8:** The proposed technique efficiency compared to other novel techniques (time in milliseconds).

Plaintext size	Proposed technique	AES	DES	[57]	[58]	[59]	[60]
50KB	104	76	288	136	222	189	295
100KB	126	121	487	191	255	199	408
250KB	165	192	777	234	319	276	871
500KB	242	255	903	397	701	609	1321
1MB	347	381	1022	678	933	964	1789
3MB	719	896	1574	1022	1341	1863	2891
6MB	2031	2253	2705	2117	2765	4099	5101

## Discussion

The training algorithm transforms the initial random weights to the encryption/ decryption keys while training the two-layer neural network. Because our training algorithm is very sensitive to the initial assigned weights and these weights are random, there is no feasible way to predict the final weights (which are the encryption/decryption keys).

The proposed encryption technique utilizes a recurrent neural network *RNN* to generate hashing values. The *RNN* method is flexible and it is always possible to increase the number of neurons for longer hash values. This is exceptionally important for avoiding any potential hashing collision (producing the same hash value for different strings). Besides, the *RNN* incurs no additional processing time because it requires no training.

The flexility of the two–layer feedforward neural network and the effective distortion layer give our technique the advantage of being elusive. From one hand, we can use the *NN* any time during the encryption to generate new encryption/decryption keys by providing initial random weights (obtained from our random generator). These new keys allow the encryption method to change the way in which it encrypts the plaintext block and therefore it is extremely infeasible to learn the encryption keys using hacking tools. On the other hand, the distortion layer imposes substantial masking by applying sound and effective operations (e.g. substitution and noising) that deeply modify both the structure of the plaintext block and the individual symbols in this block. As shown in [1, 3, 8], the substitution and nosing operations effectively cancel the correlation between the plaintext and the ciphertext. The synergistic work between the *NN* and distortion operations resulted in a highly random ciphertext that is greatly immune against effective attacks such as Differential Cryptanalysis [61] and linear Cryptanalysis [62, 63].

Augmenting the encryption technique with *RNN* (for hashing) makes our method unique as it addresses both the data confidentiality and integrity (two extremely important pillars of information security). In fact, all the security technique either addresses the confidentiality or the integrity. Our technique considers both, making it innovative.

## Conclusions and future work

This work has shown that the flexible/parallel intrinsic properties of neural networks can be part of a comprehensive system used for data encryption/decryption and hashing. It has also shown that the integration of AI-based paradigms (neural network) with the effective distortion operation can create a security system that offers a maximum protection against the confidentiality and integrity breaches. The performance analysis of the proposed system showed that it is competitive with the existing and classical systems in that field.

It has been advisable always to use hybrid systems that can carry the merits of two different techniques. As shown in the performance analysis section, the proposed system was tested using standard techniques and it showed promising results. The combination of a computational approach with a symbolic approach in one system makes it much harder for hackers to break the secrets of the encryption method. This does not need proof, it needs only common sense. Given the maximum sensitivity of the neural network to the initial weights, how can the decryption keys be predicted from the ciphertext? The only way is to test all possible initialization for the set of weights of the output layer. Each one of those weights may have an initialization between [-10, +10] and to the nearest 1^−6^ binary precision. Training time is not significant since we can reuse the generated weights (or keys) any number of times. If, for security reasons, the training must be repeated (using different initial weights), the additional time can be compensated for using some sort of pipelining.

For future work, our research will focus more on efficient techniques for training the (feedforward Neural Network) NN such as levenberg-marquardt training algorithm [50], backpropagation through time [64], genetic assisted rule-based training [65], polynomial networks training [66], artificial immune system optimization [67], ant colony optimization [68], nonlinear complementarity optimization [69], and different neural network architecture [70]. More hybrid encryption/decryption will be used utilizing more layers of the *NN* to minimize any correlations between the inputs and the outputs. More rigorous testing for our system is needed using real life applications.

## Supporting information

S1 Appendix Supporting information files for randomness testing. (PDF)

S1 Table Neural network training specifications. (PDF)

S1 Fig Complete specification of the encryption processes using neural network without the distortion process. (PDF)
