# Supplementary material for: Hybrid encryption technique: Integrating the neural network with distortion techniques
Source: PLoS One. 2022 Sep 28;17(9):e0274947. doi: 10.1371/journal.pone.0274947 (PMC9518910; doi:10.1371/journal.pone.0274947)
Supplement: S1 Appendix — (PDF) [file pone.0274947.s001.pdf]

Supporting Information files for randomness testing

1. NIST testing results

\textbf{Randomness Test} &\textbf{Success} & \textbf{Failure} & \textbf{Success rate} & \textbf{CI}\\ \hline\hline
Runs test &292& 8 &97.33\%& 26.32
Monobit test& 294 & 6& 98.00\% &26.32
Spectral test & 259& 41& 86.33\% &26.32
Serial test &276 &24 &92.00\% &26.32
Cumulative sums test &281&19&93.70\%&26.32
Linear complexity test &289 &11 &96.30\% & 26.32
Binary matrix rank test &291&9&97.00\%&26.32

\textbf{Randomness Test} & \textbf{Success} & \textbf{Failure} & \textbf{Success rate} & \textbf{CI}\\ \hline\hline
Runs test &296& 4 &98.67\%& 26.32
Monobit test& 293 & 7& 97.67\% &26.32
Spectral test & 274& 26& 91.33\% &26.32
Serial test &289 &11 &96.33\% &26.32
Cumulative sums test &287&13&95.70\%&26.32
Linear complexity test &292 &8 &97.33\% & 26.32
Binary matrix rank test &292&8&97.33\%&26.32

\textbf{Randomness Test} &\textbf{Success} & \textbf{Failure} & \textbf{Success rate} & \textbf{CI}\\
Runs test &295& 5 &98.33\%& 26.32 \\
Monobit test& 298 & 2& 99.33\% &26.32 \\
Spectral test & 281& 19& 93.67\% &26.32\\
Serial test &295 &5 &98.33\% &26.32 \\
Cumulative sums test &288&12&96.00\%&26.32\\
Linear complexity test &285 &15 &95.00\% & 26.32\\
Binary matrix rank test &287&13&95.67\%&26.32\\

2. Testing Encryption keys (sample)

- *Handcrafted keys(sample)*

Key1:0000000000000000
Key2: 1000000000000000
Key3:0100000000000000
Key4:0010000000000000
Key5:0001000000000000
Key6:0000100000000000
Key7:0000010000000000
Key8:0000001000000000
Key9:0000011000000000
Key10:1000000001000000

Key11: 1111110000000000
Key12:1111111111111111
Key13:aaaaaaaaaaaaaaaa
Key14: abaaaaaaaaaaaaaa
Key15:aabaaaaaaaaaaaaa
…
Key122: aaaaaaaaaaaaaaab
…
ZXP098981234RTes
MyPassword198756
XYZ1287ytrefds_%
…
- *Randomly generated keys(sample)*
%^$tyrv435@@se98
HA@M-,6m$7=L)*8F
,@,e%P)bP\T$FH9h
zs}'2:J@2TJ@:z=*
TTt?3jn\7fZtp+7e
XFY/y[njb.a/7m"r
he{Z5+.V!zmA{VXr
ED@D?*"as>W2]CK/
W4LYfTUB43negW6E
rUQG7YE4A4ynGtEP
XuDEpXgLJbcTv7kY
Z3GsgNJCBUAyTaJa
jCJa3uft6ZJX3gQj
xD8vYUAyBSDtFgnw
hLN8vu7ysrFgWaxp
GuTgVKtTN4adCCuW
ucwujxJNwQg5x4B7
cL37zyqmuk347mw6
rfUcBNRcJxtbxTUP
ZLQ8GCBCj8Xuac6T
r4dBvqdGVWnWFrNW
6n4X7afQw6RvqHn7
YfrPsQj8dFjZ9Pej
NJ5CXQRZUWqGEQRx
8rcc4MFrdywg7fvN
J9CacD9kJBfcCJyj
PH5fQKPxe2QNMaGe
VhxCzjkZtVQr6evM

3. Testing plaintext (binary): Snapshots

Note that each sub-plaintext (64 bytes) results from the original by changing one bit. For instance the first sub-plaintext is only one bit of the original input: “In 2017, many good things happened. But we did not hear them*#*&” , the second results from changing the second bit, and so forth.
Also we should mention that all the testing files created in the same way.

File 1: Bytes “In 2017, many good things happened. But we did not hear them*#*&”

Bits:

01001001011011100010000000110010001100000011000100110111001011000010000001101
10101100001011011100111100100100000011001110110111101101111011001000010000001
11010001101000011010010110111001100111011100110010000001101000011000010111000
00111000001100101011011100110010101100100001011100010000001000010011101010111
01000010000001110111011001010010000001100100011010010110010000100000011011100
11011110111010000100000011010000110010101100001011100100010000001110100011010
00011001010110110100101010001000110010101000100110
00001001011011100010000000110010001100000011000100110111001011000010000001101
10101100001011011100111100100100000011001110110111101101111011001000010000001
11010001101000011010010110111001100111011100110010000001101000011000010111000
00111000001100101011011100110010101100100001011100010000001000010011101010111
01000010000001110111011001010010000001100100011010010110010000100000011011100
11011110111010000100000011010000110010101100001011100100010000001110100011010
00011001010110110100101010001000110010101000100110
01**1**01001011011100010000000110010001100000011000100110111001011000010000001101
10101100001011011100111100100100000011001110110111101101111011001000010000001
11010001101000011010010110111001100111011100110010000001101000011000010111000
00111000001100101011011100110010101100100001011100010000001000010011101010111
01000010000001110111011001010010000001100100011010010110010000100000011011100
11011110111010000100000011010000110010101100001011100100010000001110100011010
00011001010110110100101010001000110010101000100110
010**1**1001011011100010000000110010001100000011000100110111001011000010000001101
10101100001011011100111100100100000011001110110111101101111011001000010000001
11010001101000011010010110111001100111011100110010000001101000011000010111000
00111000001100101011011100110010101100100001011100010000001000010011101010111
01000010000001110111011001010010000001100100011010010110010000100000011011100
11011110111010000100000011010000110010101100001011100100010000001110100011010
00011001010110110100101010001000110010101000100110
01000001011011100010000000110010001100000011000100110111001011000010000001101
10101100001011011100111100100100000011001110110111101101111011001000010000001
11010001101000011010010110111001100111011100110010000001101000011000010111000
00111000001100101011011100110010101100100001011100010000001000010011101010111
01000010000001110111011001010010000001100100011010010110010000100000011011100
11011110111010000100000011010000110010101100001011100100010000001110100011010
00011001010110110100101010001000110010101000100110
01001101011011100010000000110010001100000011000100110111001011000010000001101
10101100001011011100111100100100000011001110110111101101111011001000010000001
11010001101000011010010110111001100111011100110010000001101000011000010111000
00111000001100101011011100110010101100100001011100010000001000010011101010111
01000010000001110111011001010010000001100100011010010110010000100000011011100
11011110111010000100000011010000110010101100001011100100010000001110100011010
00011001010110110100101010001000110010101000100110
01001011011011100010000000110010001100000011000100110111001011000010000001101
10101100001011011100111100100100000011001110110111101101111011001000010000001
11010001101000011010010110111001100111011100110010000001101000011000010111000
00111000001100101011011100110010101100100001011100010000001000010011101010111
01000010000001110111011001010010000001100100011010010110010000100000011011100
11011110111010000100000011010000110010101100001011100100010000001110100011010
00011001010110110100101010001000110010101000100110
01001000011011100010000000110010001100000011000100110111001011000010000001101
10101100001011011100111100100100000011001110110111101101111011001000010000001
11010001101000011010010110111001100111011100110010000001101000011000010111000
00111000001100101011011100110010101100100001011100010000001000010011101010111
01000010000001110111011001010010000001100100011010010110010000100000011011100

11011110111010000100000011010000110010101100001011100100010000001110100011010
00011001010110110100101010001000110010101000100110
01001001111011100010000000110010001100000011000100110111001011000010000001101
10101100001011011100111100100100000011001110110111101101111011001000010000001
11010001101000011010010110111001100111011100110010000001101000011000010111000
00111000001100101011011100110010101100100001011100010000001000010011101010111
01000010000001110111011001010010000001100100011010010110010000100000011011100
11011110111010000100000011010000110010101100001011100100010000001110100011010
00011001010110110100101010001000110010101000100110
01001001001011100010000000110010001100000011000100110111001011000010000001101
10101100001011011100111100100100000011001110110111101101111011001000010000001
11010001101000011010010110111001100111011100110010000001101000011000010111000
00111000001100101011011100110010101100100001011100010000001000010011101010111
01000010000001110111011001010010000001100100011010010110010000100000011011100
11011110111010000100000011010000110010101100001011100100010000001110100011010
00011001010110110100101010001000110010101000100110
01001001010011100010000000110010001100000011000100110111001011000010000001101
10101100001011011100111100100100000011001110110111101101111011001000010000001
11010001101000011010010110111001100111011100110010000001101000011000010111000
00111000001100101011011100110010101100100001011100010000001000010011101010111
01000010000001110111011001010010000001100100011010010110010000100000011011100
11011110111010000100000011010000110010101100001011100100010000001110100011010
00011001010110110100101010001000110010101000100110
01001001011111110001000000110010001100000011000100110111001011000010000001101
10101100001011011100111100100100000011001110110111101101111011001000010000001
11010001101000011010010110111001100111011100110010000001101000011000010111000
00111000001100101011011100110010101100100001011100010000001000010011101010111
01000010000001110111011001010010000001100100011010010110010000100000011011100
11011110111010000100000011010000110010101100001011100100010000001110100011010
00011001010110110100101010001000110010101000100110
01001001011001100010000000110010001100000011000100110111001011000010000001101
10101100001011011100111100100100000011001110110111101101111011001000010000001
11010001101000011010010110111001100111011100110010000001101000011000010111000
00111000001100101011011100110010101100100001011100010000001000010011101010111
01000010000001110111011001010010000001100100011010010110010000100000011011100
11011110111010000100000011010000110010101100001011100100010000001110100011010
00011001010110110100101010001000110010101000100110
01001001011010100010000000110010001100000011000100110111001011000010000001101
10101100001011011100111100100100000011001110110111101101111011001000010000001
11010001101000011010010110111001100111011100110010000001101000011000010111000
00111000001100101011011100110010101100100001011100010000001000010011101010111
01000010000001110111011001010010000001100100011010010110010000100000011011100
11011110111010000100000011010000110010101100001011100100010000001110100011010
00011001010110110100101010001000110010101000100110
01001001011011000010000000110010001100000011000100110111001011000010000001101
10101100001011011100111100100100000011001110110111101101111011001000010000001
11010001101000011010010110111001100111011100110010000001101000011000010111000
00111000001100101011011100110010101100100001011100010000001000010011101010111
01000010000001110111011001010010000001100100011010010110010000100000011011100
11011110111010000100000011010000110010101100001011100100010000001110100011010
00011001010110110100101010001000110010101000100110
01001001011011110010000000110010001100000011000100110111001011000010000001101
10101100001011011100111100100100000011001110110111101101111011001000010000001
11010001101000011010010110111001100111011100110010000001101000011000010111000
00111000001100101011011100110010101100100001011100010000001000010011101010111
01000010000001110111011001010010000001100100011010010110010000100000011011100

11011110111010000100000011010000110010101100001011100100010000001110100011010
00011001010110110100101010001000110010101000100110
01001001011011101010000000110010001100000011000100110111001011000010000001101
10101100001011011100111100100100000011001110110111101101111011001000010000001
11010001101000011010010110111001100111011100110010000001101000011000010111000
00111000001100101011011100110010101100100001011100010000001000010011101010111
01000010000001110111011001010010000001100100011010010110010000100000011011100
11011110111010000100000011010000110010101100001011100100010000001110100011010
00011001010110110100101010001000110010101000100110
01001001011011100110000000110010001100000011000100110111001011000010000001101
10101100001011011100111100100100000011001110110111101101111011001000010000001
11010001101000011010010110111001100111011100110010000001101000011000010111000
00111000001100101011011100110010101100100001011100010000001000010011101010111
01000010000001110111011001010010000001100100011010010110010000100000011011100
11011110111010000100000011010000110010101100001011100100010000001110100011010
00011001010110110100101010001000110010101000100110
01001001011011100000000000110010001100000011000100110111001011000010000001101
10101100001011011100111100100100000011001110110111101101111011001000010000001
11010001101000011010010110111001100111011100110010000001101000011000010111000
00111000001100101011011100110010101100100001011100010000001000010011101010111
01000010000001110111011001010010000001100100011010010110010000100000011011100
11011110111010000100000011010000110010101100001011100100010000001110100011010
00011001010110110100101010001000110010101000100110
01001001011011100011000000110010001100000011000100110111001011000010000001101
10101100001011011100111100100100000011001110110111101101111011001000010000001
11010001101000011010010110111001100111011100110010000001101000011000010111000
00111000001100101011011100110010101100100001011100010000001000010011101010111
01000010000001110111011001010010000001100100011010010110010000100000011011100
11011110111010000100000011010000110010101100001011100100010000001110100011010
00011001010110110100101010001000110010101000100110
01001001011011100010100000110010001100000011000100110111001011000010000001101
10101100001011011100111100100100000011001110110111101101111011001000010000001
11010001101000011010010110111001100111011100110010000001101000011000010111000
00111000001100101011011100110010101100100001011100010000001000010011101010111
01000010000001110111011001010010000001100100011010010110010000100000011011100
11011110111010000100000011010000110010101100001011100100010000001110100011010
00011001010110110100101010001000110010101000100110
01001001011011100010010000110010001100000011000100110111001011000010000001101
10101100001011011100111100100100000011001110110111101101111011001000010000001
11010001101000011010010110111001100111011100110010000001101000011000010111000
00111000001100101011011100110010101100100001011100010000001000010011101010111
01000010000001110111011001010010000001100100011010010110010000100000011011100
11011110111010000100000011010000110010101100001011100100010000001110100011010
00011001010110110100101010001000110010101000100110
01001001011011100010001000110010001100000011000100110111001011000010000001101
10101100001011011100111100100100000011001110110111101101111011001000010000001
11010001101000011010010110111001100111011100110010000001101000011000010111000
00111000001100101011011100110010101100100001011100010000001000010011101010111
01000010000001110111011001010010000001100100011010010110010000100000011011100
11011110111010000100000011010000110010101100001011100100010000001110100011010
00011001010110110100101010001000110010101000100110
01001001011011100010000100110010001100000011000100110111001011000010000001101
10101100001011011100111100100100000011001110110111101101111011001000010000001
11010001101000011010010110111001100111011100110010000001101000011000010111000
00111000001100101011011100110010101100100001011100010000001000010011101010111
01000010000001110111011001010010000001100100011010010110010000100000011011100

1101111011101000010000000110100001100101011000010111001000100000001110100011010
00011001010110110100101010001000110010101000100110
0100100101101110001000000101100100011000000110001001101110010110000100000001101
1010110000101101110011110010010000001100111011011110110111101100100001000000 1
1101000110100001101001011011100110011101110011001000000011010000110000101110 00
0011100000110010101101110011001010110010000101110001000000010000100111010101 11
0100001000000111011101100101001000000110010001101001011001000010000000110111 00
1101111011101000010000000110100001100101011000010111001000100000001110100011010
00011001010110110100101010001000110010101000100110
0100100101101110001000000111001000110000001100010011011100101100001000000 01101
1010110000101101110011110010010000001100111011011110110111101100100001000000 1
1101000110100001101001011011100110011101110011001000000011010000110000101110 00
0011100000110010101101110011001010110010000101110001000000010000100111010101 11
0100001000000111011101100101001000000110010001101001011001000010000000110111 00
1101111011101000010000000110100001100101011000010111001000100000001110100011010
00011001010110110100101010001000110010101000100110
01001001011011100010000000001001000110000001100010011011100101100001000000 01101
1010110000101101110011110010010000001100111011011110110111101100100001000000 1
1101000110100001101001011011100110011101110011001000000011010000110000101110 00
0011100000110010101101110011001010110010000101110001000000010000100111010101 11
0100001000000111011101100101001000000110010001101001011001000010000000110111 00
1101111011101000010000000110100001100101011000010111001000100000001110100011010
00011001010110110100101010001000110010101000100110
01001001011011100010000000100010001100000011000100110111001011000010000000 1101
1010110000101101110011110010010000001100111011011110110111101100100001000000 1
1101000110100001101001011011100110011101110011001000000011010000110000101110 00
0011100000110010101101110011001010110010000101110001000000010000100111010101 11
0100001000000111011101100101001000000110010001101001011001000010000000110111 00
1101111011101000010000000110100001100101011000010111001000100000001110100011010
00011001010110110100101010001000110010101000100110
0100100101101110001000000111010001100000011000100110111001011000010000000 1101
1010110000101101110011110010010000001100111011011110110111101100100001000000 1
1101000110100001101001011011100110011101110011001000000011010000110000101110 00
0011100000110010101101110011001010110010000101110001000000010000100111010101 11
0100001000000111011101100101001000000110010001101001011001000010000000110111 00
1101111011101000010000000110100001100101011000010111001000100000001110100011010
00011001010110110100101010001000110010101000100110
0100100101101110001000000110110001100000011000100110111001011000010000000 1101
1010110000101101110011110010010000001100111011011110110111101100100001000000 1
1101000110100001101001011011100110011101110011001000000011010000110000101110 00
0011100000110010101101110011001010110010000101110001000000010000100111010101 11
0100001000000111011101100101001000000110010001101001011001000010000000110111 00
1101111011101000010000000110100001100101011000010111001000100000001110100011010
00011001010110110100101010001000110010101000100110
0100100101101110001000000011000000110000001100010011011100101100001000000 01101
1010110000101101110011110010010000001100111011011110110111101100100001000000 1
1101000110100001101001011011100110011101110011001000000011010000110000101110 00
0011100000110010101101110011001010110010000101110001000000010000100111010101 11
0100001000000111011101100101001000000110010001101001011001000010000000110111 00
1101111011101000010000000110100001100101011000010111001000100000001110100011010
00011001010110110100101010001000110010101000100110
0100100101101110001000000011001100110000001100010011011100101100001000000 01101
1010110000101101110011110010010000001100111011011110110111101100100001000000 1
1101000110100001101001011011100110011101110011001000000011010000110000101110 00
0011100000110010101101110011001010110010000101110001000000010000100111010101 11
0100001000000111011101100101001000000110010001101001011001000010000000110111 00

11011110111010000100000011010000110010101100001011100100010000001110100011010
00011001010110110100101010001000110010101000100110
01001001011011100010000000110010101100000011000100110111001011000010000001101
10101100001011011100111100100100000011001110110111101101111011001000010000001
11010001101000011010010110111001100111011100110010000001101000011000010111000
00111000001100101011011100110010101100100001011100010000001000010011101010111
01000010000001110111011001010010000001100100011010010110010000100000011011100
11011110111010000100000011010000110010101100001011100100010000001110100011010
00011001010110110100101010001000110010101000100110
01001001011011100010000000110010011100000011000100110111001011000010000001101
10101100001011011100111100100100000011001110110111101101111011001000010000001
11010001101000011010010110111001100111011100110010000001101000011000010111000
00111000001100101011011100110010101100100001011100010000001000010011101010111
01000010000001110111011001010010000001100100011010010110010000100000011011100
11011110111010000100000011010000110010101100001011100100010000001110100011010
00011001010110110100101010001000110010101000100110
01001001011011100010000000110010000100000011000100110111001011000010000001101
10101100001011011100111100100100000011001110110111101101111011001000010000001
11010001101000011010010110111001100111011100110010000001101000011000010111000
00111000001100101011011100110010101100100001011100010000001000010011101010111
01000010000001110111011001010010000001100100011010010110010000100000011011100
11011110111010000100000011010000110010101100001011100100010000001110100011010
00011001010110110100101010001000110010101000100110
01001001011011100010000000110010001000000011000100110111001011000010000001101
10101100001011011100111100100100000011001110110111101101111011001000010000001
11010001101000011010010110111001100111011100110010000001101000011000010111000
00111000001100101011011100110010101100100001011100010000001000010011101010111
01000010000001110111011001010010000001100100011010010110010000100000011011100
11011110111010000100000011010000110010101100001011100100010000001110100011010
00011001010110110100101010001000110010101000100110
01001001011011100010000000110010001110000011000100110111001011000010000001101
10101100001011011100111100100100000011001110110111101101111011001000010000001
11010001101000011010010110111001100111011100110010000001101000011000010111000
00111000001100101011011100110010101100100001011100010000001000010011101010111
01000010000001110111011001010010000001100100011010010110010000100000011011100
11011110111010000100000011010000110010101100001011100100010000001110100011010
00011001010110110100101010001000110010101000100110
01001001011011100010000000110010001101000011000100110111001011000010000001101
10101100001011011100111100100100000011001110110111101101111011001000010000001
11010001101000011010010110111001100111011100110010000001101000011000010111000
00111000001100101011011100110010101100100001011100010000001000010011101010111
01000010000001110111011001010010000001100100011010010110010000100000011011100
11011110111010000100000011010000110010101100001011100100010000001110100011010
00011001010110110100101010001000110010101000100110
01001001011011100010000000110010001100100011000100110111001011000010000001101
10101100001011011100111100100100000011001110110111101101111011001000010000001
11010001101000011010010110111001100111011100110010000001101000011000010111000
00111000001100101011011100110010101100100001011100010000001000010011101010111
01000010000001110111011001010010000001100100011010010110010000100000011011100
11011110111010000100000011010000110010101100001011100100010000001110100011010
00011001010110110100101010001000110010101000100110
01001001011011100010000000110010001100010011000100110111001011000010000001101
10101100001011011100111100100100000011001110110111101101111011001000010000001
11010001101000011010010110111001100111011100110010000001101000011000010111000
00111000001100101011011100110010101100100001011100010000001000010011101010111
01000010000001110111011001010010000001100100011010010110010000100000011011100

11011110111010000100000011010000110010101100001011100100010000001110100011010
00011001010110110100101010001000110010101000100110
01001001011011100010000000110010001100001011000100110111001011000010000001101
10101100001011011100111100100100000011001110110111101101111011001000010000001
11010001101000011010010110111001100111011100110010000001101000011000010111000
00111000001100101011011100110010101100100001011100010000001000010011101010111
01000010000001110111011001010010000001100100011010010110010000100000011011100
11011110111010000100000011010000110010101100001011100100010000001110100011010
00011001010110110100101010001000110010101000100110
01001001011011100010000000110010001100000111000100110111001011000010000001101
10101100001011011100111100100100000011001110110111101101111011001000010000001
11010001101000011010010110111001100111011100110010000001101000011000010111000
00111000001100101011011100110010101100100001011100010000001000010011101010111
01000010000001110111011001010010000001100100011010010110010000100000011011100
11011110111010000100000011010000110010101100001011100100010000001110100011010
00011001010110110100101010001000110010101000100110
01001001011011100010000000110010001100000001000100110111001011000010000001101
10101100001011011100111100100100000011001110110111101101111011001000010000001
11010001101000011010010110111001100111011100110010000001101000011000010111000
00111000001100101011011100110010101100100001011100010000001000010011101010111
01000010000001110111011001010010000001100100011010010110010000100000011011100
11011110111010000100000011010000110010101100001011100100010000001110100011010
00011001010110110100101010001000110010101000100110
01001001011011100010000000110010001100000010000100110111001011000010000001101
10101100001011011100111100100100000011001110110111101101111011001000010000001
11010001101000011010010110111001100111011100110010000001101000011000010111000
00111000001100101011011100110010101100100001011100010000001000010011101010111
01000010000001110111011001010010000001100100011010010110010000100000011011100
11011110111010000100000011010000110010101100001011100100010000001110100011010
00011001010110110100101010001000110010101000100110
01001001011011100010000000110010001100000011100100110111001011000010000001101
10101100001011011100111100100100000011001110110111101101111011001000010000001
11010001101000011010010110111001100111011100110010000001101000011000010111000
00111000001100101011011100110010101100100001011100010000001000010011101010111
01000010000001110111011001010010000001100100011010010110010000100000011011100
11011110111010000100000011010000110010101100001011100100010000001110100011010
00011001010110110100101010001000110010101000100110
01001001011011100010000000110010001100000011010100110111001011000010000001101
10101100001011011100111100100100000011001110110111101101111011001000010000001
11010001101000011010010110111001100111011100110010000001101000011000010111000
00111000001100101011011100110010101100100001011100010000001000010011101010111
01000010000001110111011001010010000001100100011010010110010000100000011011100
11011110111010000100000011010000110010101100001011100100010000001110100011010
00011001010110110100101010001000110010101000100110
01001001011011100010000000110010001100000011001100110111001011000010000001101
10101100001011011100111100100100000011001110110111101101111011001000010000001
11010001101000011010010110111001100111011100110010000001101000011000010111000
00111000001100101011011100110010101100100001011100010000001000010011101010111
01000010000001110111011001010010000001100100011010010110010000100000011011100
11011110111010000100000011010000110010101100001011100100010000001110100011010
00011001010110110100101010001000110010101000100110
01001001011011100010000000110010001100000011000000110111001011000010000001101
10101100001011011100111100100100000011001110110111101101111011001000010000001
11010001101000011010010110111001100111011100110010000001101000011000010111000
00111000001100101011011100110010101100100001011100010000001000010011101010111
01000010000001110111011001010010000001100100011010010110010000100000011011100

11011110111010000100000011010000110010101100001011100100010000001110100011010
00011001010110110100101010001000110010101000100110
01001001011011100010000000110010001100000011000110110111001011000010000001101
10101100001011011100111100100100000011001110110111101101111011001000010000001
11010001101000011010010110111001100111011100110010000001101000011000010111000
00111000000110010101101110011001010110010000101110001000000100001001110101 0111
01000010000001110111011001010010000001100100011010010110010000100000011011100
11011110111010000100000011010000110010101100001011100100010000001110100011010
00011001010110110100101010001000110010101000100110
01001001011011100010000000110010001100000011000101110111001011000010000001101
10101100001011011100111100100100000011001110110111101101111011001000010000001
11010001101000011010010110111001100111011100110010000001101000011000010111000
00111000000110010101101110011001010110010000101110001000000100001001110101 0111
01000010000001110111011001010010000001100100011010010110010000100000011011100
11011110111010000100000011010000110010101100001011100100010000001110100011010
00011001010110110100101010001000110010101000100110
01001001011011100010000000110010001100000011000100010111001011000010000001101
10101100001011011100111100100100000011001110110111101101111011001000010000001
11010001101000011010010110111001100111011100110010000001101000011000010111000
00111000000110010101101110011001010110010000101110001000000100001001110101 0111
01000010000001110111011001010010000001100100011010010110010000100000011011100
11011110111010000100000011010000110010101100001011100100010000001110100011010
00011001010110110100101010001000110010101000100110
01001001011011100010000000110010001100000011000100100111001011000010000001101
10101100001011011100111100100100000011001110110111101101111011001000010000001
11010001101000011010010110111001100111011100110010000001101000011000010111000
00111000000110010101101110011001010110010000101110001000000100001001110101 0111
01000010000001110111011001010010000001100100011010010110010000100000011011100
11011110111010000100000011010000110010101100001011100100010000001110100011010
00011001010110110100101010001000110010101000100110
01001001011011100010000000110010001100000011000100111111001011000010000001101
10101100001011011100111100100100000011001110110111101101111011001000010000001
11010001101000011010010110111001100111011100110010000001101000011000010111000
00111000000110010101101110011001010110010000101110001000000100001001110101 0111
01000010000001110111011001010010000001100100011010010110010000100000011011100
11011110111010000100000011010000110010101100001011100100010000001110100011010
00011001010110110100101010001000110010101000100110
01001001011011100010000000110010001100000011000100110011001011000010000001101
10101100001011011100111100100100000011001110110111101101111011001000010000001
11010001101000011010010110111001100111011100110010000001101000011000010111000
00111000000110010101101110011001010110010000101110001000000100001001110101 0111
01000010000001110111011001010010000001100100011010010110010000100000011011100
11011110111010000100000011010000110010101100001011100100010000001110100011010
00011001010110110100101010001000110010101000100110
01001001011011100010000000110010001100000011000100110101001011000010000001101
10101100001011011100111100100100000011001110110111101101111011001000010000001
11010001101000011010010110111001100111011100110010000001101000011000010111000
00111000000110010101101110011001010110010000101110001000000100001001110101 0111
01000010000001110111011001010010000001100100011010010110010000100000011011100
11011110111010000100000011010000110010101100001011100100010000001110100011010
00011001010110110100101010001000110010101000100110
01001001011011100010000000110010001100000011000100110110001011000010000001101
10101100001011011100111100100100000011001110110111101101111011001000010000001
11010001101000011010010110111001100111011100110010000001101000011000010111000
00111000000110010101101110011001010110010000101110001000000100001001110101 0111
01000010000001110111011001010010000001100100011010010110010000100000011011100

11011110111010000100000011010000110010101100001011100100010000001110100011010
00011001010110110100101010001000110010101000100110
01001001011011100010000000110010001100000011000100110111101011000010000001101
10101100001011011100111100100100000011001110110111101101111011001000010000001
11010001101000011010010110111001100111011100110010000001101000011000010111000
00111000001100101011011100110010101100100001011100010000001000010011101010111
01000010000001110111011001010010000001100100011010010110010000100000011011100
11011110111010000100000011010000110010101100001011100100010000001110100011010
00011001010110110100101010001000110010101000100110
01001001011011100010000000110010001100000011000100110111011011000010000001101
10101100001011011100111100100100000011001110110111101101111011001000010000001
11010001101000011010010110111001100111011100110010000001101000011000010111000
00111000001100101011011100110010101100100001011100010000001000010011101010111
01000010000001110111011001010010000001100100011010010110010000100000011011100
11011110111010000100000011010000110010101100001011100100010000001110100011010
00011001010110110100101010001000110010101000100110
01001001011011100010000000110010001100000011000100110111000011000010000001101
10101100001011011100111100100100000011001110110111101101111011001000010000001
11010001101000011010010110111001100111011100110010000001101000011000010111000
00111000001100101011011100110010101100100001011100010000001000010011101010111
01000010000001110111011001010010000001100100011010010110010000100000011011100
11011110111010000100000011010000110010101100001011100100010000001110100011010
00011001010110110100101010001000110010101000100110
01001001011011100010000000110010001100000011000100110111001111000010000001101
10101100001011011100111100100100000011001110110111101101111011001000010000001
11010001101000011010010110111001100111011100110010000001101000011000010111000
00111000001100101011011100110010101100100001011100010000001000010011101010111
01000010000001110111011001010010000001100100011010010110010000100000011011100
11011110111010000100000011010000110010101100001011100100010000001110100011010
00011001010110110100101010001000110010101000100110
01001001011011100010000000110010001100000011000100110111001001000010000001101
10101100001011011100111100100100000011001110110111101101111011001000010000001
11010001101000011010010110111001100111011100110010000001101000011000010111000
00111000001100101011011100110010101100100001011100010000001000010011101010111
01000010000001110111011001010010000001100100011010010110010000100000011011100
11011110111010000100000011010000110010101100001011100100010000001110100011010
00011001010110110100101010001000110010101000100110
01001001011011100010000000110010001100000011000100110111001010000010000001101
10101100001011011100111100100100000011001110110111101101111011001000010000001
11010001101000011010010110111001100111011100110010000001101000011000010111000
00111000001100101011011100110010101100100001011100010000001000010011101010111
01000010000001110111011001010010000001100100011010010110010000100000011011100
11011110111010000100000011010000110010101100001011100100010000001110100011010
00011001010110110100101010001000110010101000100110
01001001011011100010000000110010001100000011000100110111001011100010000001101
10101100001011011100111100100100000011001110110111101101111011001000010000001
11010001101000011010010110111001100111011100110010000001101000011000010111000
00111000001100101011011100110010101100100001011100010000001000010011101010111
01000010000001110111011001010010000001100100011010010110010000100000011011100
11011110111010000100000011010000110010101100001011100100010000001110100011010
00011001010110110100101010001000110010101000100110
01001001011011100010000000110010001100000011000100110111001011010010000001101
10101100001011011100111100100100000011001110110111101101111011001000010000001
11010001101000011010010110111001100111011100110010000001101000011000010111000
00111000001100101011011100110010101100100001011100010000001000010011101010111
01000010000001110111011001010010000001100100011010010110010000100000011011100

11011110111010000100000011010000110010101100001011100100010000001110100011010
0001100101011011010010101000100011001010100010011 0
01001001011011100010000000110010001100000011000100110111001011001010000001101
10101100001011011100111100100100000011001110110111101101111011001000010000001
11010001101000011010010110111001100111011100110010000001101000011000010111000
00111000001100101011011100110010101100100001011100010000000100001001110101011 1
01000010000001110111011001010010000001100100011010010110010000100000011011100
11011110111010000100000011010000110010101100001011100100010000001110100011010
0001100101011011010010101000100011001010100010011 0
01001001011011100010000000110010001100000011000100110111001011000110000001101
10101100001011011100111100100100000011001110110111101101111011001000010000001
11010001101000011010010110111001100111011100110010000001101000011000010111000
00111000001100101011011100110010101100100001011100010000000100001001110101011 1
01000010000001110111011001010010000001100100011010010110010000100000011011100
11011110111010000100000011010000110010101100001011100100010000001110100011010
0001100101011011010010101000100011001010100010011 0
01001001011011100010000000110010001100000011000100110111001011000000000001101
10101100001011011100111100100100000011001110110111101101111011001000010000001
11010001101000011010010110111001100111011100110010000001101000011000010111000
00111000001100101011011100110010101100100001011100010000000100001001110101011 1
01000010000001110111011001010010000001100100011010010110010000100000011011100
11011110111010000100000011010000110010101100001011100100010000001110100011010
0001100101011011010010101000100011001010100010011 0
01001001011011100010000000110010001100000011000100110111001011000011000001101
10101100001011011100111100100100000011001110110111101101111011001000010000001
11010001101000011010010110111001100111011100110010000001101000011000010111000
00111000001100101011011100110010101100100001011100010000000100001001110101011 1
01000010000001110111011001010010000001100100011010010110010000100000011011100
11011110111010000100000011010000110010101100001011100100010000001110100011010
0001100101011011010010101000100011001010100010011 0
01001001011011100010000000110010001100000011000100110111001011000010100001101
10101100001011011100111100100100000011001110110111101101111011001000010000001
11010001101000011010010110111001100111011100110010000001101000011000010111000
00111000001100101011011100110010101100100001011100010000000100001001110101011 1
01000010000001110111011001010010000001100100011010010110010000100000011011100
11011110111010000100000011010000110010101100001011100100010000001110100011010
0001100101011011010010101000100011001010100010011 0
01001001011011100010000000110010001100000011000100110111001011000010010001101
10101100001011011100111100100100000011001110110111101101111011001000010000001
11010001101000011010010110111001100111011100110010000001101000011000010111000
00111000001100101011011100110010101100100001011100010000000100001001110101011 1
01000010000001110111011001010010000001100100011010010110010000100000011011100
11011110111010000100000011010000110010101100001011100100010000001110100011010
0001100101011011010010101000100011001010100010011 0
01001001011011100010000000110010001100000011000100110111001011000010001001101
10101100001011011100111100100100000011001110110111101101111011001000010000001
11010001101000011010010110111001100111011100110010000001101000011000010111000
00111000001100101011011100110010101100100001011100010000000100001001110101011 1
01000010000001110111011001010010000001100100011010010110010000100000011011100
11011110111010000100000011010000110010101100001011100100010000001110100011010
0001100101011011010010101000100011001010100010011 0
01001001011011100010000000110010001100000011000100110111001011000010000101101
10101100001011011100111100100100000011001110110111101101111011001000010000001
11010001101000011010010110111001100111011100110010000001101000011000010111000
00111000001100101011011100110010101100100001011100010000000100001001110101011 1
01000010000001110111011001010010000001100100011010010110010000100000011011100

11011110111010000100000011010000110010101100001011100100010000001110100011010
00011001010110110100101010001000110010101000100110
01001001011011100010000000110010001100000011000100110111001011000010000011101
10101100001011011100111100100100000011001110110111101101111011001000010000001
11010001101000011010010110111001100111011100110010000001101000011000010111000
00111000001100101011011100110010101100100001011100010000000100001001110101011 1
01000010000000111011101100101001000000110010001101001011001000010000000110111 00
11011110111010000100000011010000110010101100001011100100010000001110100011010
00011001010110110100101010001000110010101000100110
01001001011011100010000000110010001100000011000100110111001011000010000000101
10101100001011011100111100100100000011001110110111101101111011001000010000001
11010001101000011010010110111001100111011100110010000001101000011000010111000
00111000001100101011011100110010101100100001011100010000000100001001110101011 1
01000010000000111011101100101001000000110010001101001011001000010000000110111 00
11011110111010000100000011010000110010101100001011100100010000001110100011010
00011001010110110100101010001000110010101000100110
01001001011011100010000000110010001100000011000100110111001011000010000001001
10101100001011011100111100100100000011001110110111101101111011001000010000001
11010001101000011010010110111001100111011100110010000001101000011000010111000
00111000001100101011011100110010101100100001011100010000000100001001110101011 1
01000010000000111011101100101001000000110010001101001011001000010000000110111 00
11011110111010000100000011010000110010101100001011100100010000001110100011010
00011001010110110100101010001000110010101000100110
01001001011011100010000000110010001100000011000100110111001011000010000001111
10101100001011011100111100100100000011001110110111101101111011001000010000001
11010001101000011010010110111001100111011100110010000001101000011000010111000
00111000001100101011011100110010101100100001011100010000000100001001110101011 1
01000010000000111011101100101001000000110010001101001011001000010000000110111 00
11011110111010000100000011010000110010101100001011100100010000001110100011010
00011001010110110100101010001000110010101000100110
01001001011011100010000000110010001100000011000100110111001011000010000001100
10101100001011011100111100100100000011001110110111101101111011001000010000001
11010001101000011010010110111001100111011100110010000001101000011000010111000
00111000001100101011011100110010101100100001011100010000000100001001110101011 1
01000010000000111011101100101001000000110010001101001011001000010000000110111 00
11011110111010000100000011010000110010101100001011100100010000001110100011010
00011001010110110100101010001000110010101000100110
01001001011011100010000000110010001100000011000100110111001011000010000001101
00101100001011011100111100100100000011001110110111101101111011001000010000001
11010001101000011010010110111001100111011100110010000001101000011000010111000
00111000001100101011011100110010101100100001011100010000000100001001110101011 1
01000010000000111011101100101001000000110010001101001011001000010000000110111 00
11011110111010000100000011010000110010101100001011100100010000001110100011010
00011001010110110100101010001000110010101000100110
01001001011011100010000000110010001100000011000100110111001011000010000001101
11101100001011011100111100100100000011001110110111101101111011001000010000001
11010001101000011010010110111001100111011100110010000001101000011000010111000
00111000001100101011011100110010101100100001011100010000000100001001110101011 1
01000010000000111011101100101001000000110010001101001011001000010000000110111 00
11011110111010000100000011010000110010101100001011100100010000001110100011010
00011001010110110100101010001000110010101000100110
01001001011011100010000000110010001100000011000100110111001011000010000001101
10001100001011011100111100100100000011001110110111101101111011001000010000001
11010001101000011010010110111001100111011100110010000001101000011000010111000
00111000001100101011011100110010101100100001011100010000000100001001110101011 1
01000010000000111011101100101001000000110010001101001011001000010000000110111 00

1101111011101000010000000110100001100101011000010111001000100000001110100011010
00011001010110110100101010001000110010101000100110
0100100101101110001000000001100100011000000110001001101110010110000100000001101
101111100001011011100111100100100000011001110110111101101111011001000010000001
11010001101000011010010110111001100111011100110010000001101000011000010111000
001110000011001010110111001100101011001000010111000100000001000010011101010111
010000100000001110111011001010010000001100100011010010110010000100000011011100
1101111011101000010000000110100001100101011000010111001000100000001110100011010
00011001010110110100101010001000110010101000100110
0100100101101110001000000001100100011000000110001001101110010110000100000001101
10100100001011011100111100100100000011001110110111101101111011001000010000001
11010001101000011010010110111001100111011100110010000001101000011000010111000
001110000011001010110111001100101011001000010111000100000001000010011101010111
010000100000001110111011001010010000001100100011010010110010000100000011011100
1101111011101000010000000110100001100101011000010111001000100000001110100011010
00011001010110110100101010001000110010101000100110
0100100101101110001000000001100100011000000110001001101110010110000100000001101
101010000001011011100111100100100000011001110110111101101111011001000010000001
11010001101000011010010110111001100111011100110010000001101000011000010111000
001110000011001010110111001100101011001000010111000100000001000010011101010111
010000100000001110111011001010010000001100100011010010110010000100000011011100
1101111011101000010000000110100001100101011000010111001000100000001110100011010
00011001010110110100101010001000110010101000100110
0100100101101110001000000001100100011000000110001001101110010110000100000001101
101011100001011011100111100100100000011001110110111101101111011001000010000001
11010001101000011010010110111001100111011100110010000001101000011000010111000
001110000011001010110111001100101011001000010111000100000001000010011101010111
010000100000001110111011001010010000001100100011010010110010000100000011011100
1101111011101000010000000110100001100101011000010111001000100000001110100011010
00011001010110110100101010001000110010101000100110
0100100101101110001000000001100100011000000110001001101110010110000100000001101
10101101001011011100111100100100000011001110110111101101111011001000010000001
11010001101000011010010110111001100111011100110010000001101000011000010111000
001110000011001010110111001100101011001000010111000100000001000010011101010111
010000100000001110111011001010010000001100100011010010110010000100000011011100
1101111011101000010000000110100001100101011000010111001000100000001110100011010
00011001010110110100101010001000110010101000100110
0100100101101110001000000001100100011000000110001001101110010110000100000001101
10101100101011011100111100100100000011001110110111101101111011001000010000001
11010001101000011010010110111001100111011100110010000001101000011000010111000
001110000011001010110111001100101011001000010111000100000001000010011101010111
010000100000001110111011001010010000001100100011010010110010000100000011011100
1101111011101000010000000110100001100101011000010111001000100000001110100011010
00011001010110110100101010001000110010101000100110
0100100101101110001000000001100100011000000110001001101110010110000100000001101
10101100011011011100111100100100000011001110110111101101111011001000010000001
11010001101000011010010110111001100111011100110010000001101000011000010111000
001110000011001010110111001100101011001000010111000100000001000010011101010111
010000100000001110111011001010010000001100100011010010110010000100000011011100
1101111011101000010000000110100001100101011000010111001000100000001110100011010
00011001010110110100101010001000110010101000100110
0100100101101110001000000001100100011000000110001001101110010110000100000001101
10101100000011011100111100100100000011001110110111101101111011001000010000001
11010001101000011010010110111001100111011100110010000001101000011000010111000
001110000011001010110111001100101011001000010111000100000001000010011101010111
010000100000001110111011001010010000001100100011010010110010000100000011011100

11011110111010000100000011010000110010101100001011100100010000001110100011010
00011001010110110100101010001000110010101000100110
01001001011011100010000000110010001100000011000100110111001011000010000001101
10101100001111011100111100100100000011001110110111101101111011001000010000001
11010001101000011010010110111001100111011100110010000001101000011000010111000
001110000011001010110111001100101011001000010111000100000001000010011101010111
01000010000001110111011001010010000001100100011010010110010000100000011011100
11011110111010000100000011010000110010101100001011100100010000001110100011010
00011001010110110100101010001000110010101000100110
01001001011011100010000000110010001100000011000100110111001011000010000001101
10101100001001011100111100100100000011001110110111101101111011001000010000001
11010001101000011010010110111001100111011100110010000001101000011000010111000
001110000011001010110111001100101011001000010111000100000001000010011101010111
01000010000001110111011001010010000001100100011010010110010000100000011011100
11011110111010000100000011010000110010101100001011100100010000001110100011010
00011001010110110100101010001000110010101000100110
01001001011011100010000000110010001100000011000100110111001011000010000001101
10101100001010011100111100100100000011001110110111101101111011001000010000001
11010001101000011010010110111001100111011100110010000001101000011000010111000
001110000011001010110111001100101011001000010111000100000001000010011101010111
01000010000001110111011001010010000001100100011010010110010000100000011011100
11011110111010000100000011010000110010101100001011100100010000001110100011010
00011001010110110100101010001000110010101000100110
01001001011011100010000000110010001100000011000100110111001011000010000001101
10101100001011111100111100100100000011001110110111101101111011001000010000001
11010001101000011010010110111001100111011100110010000001101000011000010111000
001110000011001010110111001100101011001000010111000100000001000010011101010111
01000010000001110111011001010010000001100100011010010110010000100000011011100
11011110111010000100000011010000110010101100001011100100010000001110100011010
00011001010110110100101010001000110010101000100110
01001001011011100010000000110010001100000011000100110111001011000010000001101
10101100001011001100111100100100000011001110110111101101111011001000010000001
11010001101000011010010110111001100111011100110010000001101000011000010111000
001110000011001010110111001100101011001000010111000100000001000010011101010111
01000010000001110111011001010010000001100100011010010110010000100000011011100
11011110111010000100000011010000110010101100001011100100010000001110100011010
00011001010110110100101010001000110010101000100110
01001001011011100010000000110010001100000011000100110111001011000010000001101
10101100001011010100111100100100000011001110110111101101111011001000010000001
11010001101000011010010110111001100111011100110010000001101000011000010111000
001110000011001010110111001100101011001000010111000100000001000010011101010111
01000010000001110111011001010010000001100100011010010110010000100000011011100
11011110111010000100000011010000110010101100001011100100010000001110100011010
00011001010110110100101010001000110010101000100110
01001001011011100010000000110010001100000011000100110111001011000010000001101
10101100001011011000111100100100000011001110110111101101111011001000010000001
11010001101000011010010110111001100111011100110010000001101000011000010111000
001110000011001010110111001100101011001000010111000100000001000010011101010111
01000010000001110111011001010010000001100100011010010110010000100000011011100
11011110111010000100000011010000110010101100001011100100010000001110100011010
00011001010110110100101010001000110010101000100110
01001001011011100010000000110010001100000011000100110111001011000010000001101
10101100001011011110111100100100000011001110110111101101111011001000010000001
11010001101000011010010110111001100111011100110010000001101000011000010111000
001110000011001010110111001100101011001000010111000100000001000010011101010111
01000010000001110111011001010010000001100100011010010110010000100000011011100

11011110111010000100000011010000110010101100001011100100010000001110100011010
00011001010110110100101010001000110010101000100110
01001001011011100010000000110010001100000011000100110111001011000010000001101
10101100001011011101111100100100000011001110110111101101111011001000010000001
11010001101000011010010110111001100111011100110010000001101000011000010111000
00111000001100101011011100110010101100100001011100010000001000010011101010111
01000010000001110111011001010010000001100100011010010110010000100000011011100
11011110111010000100000011010000110010101100001011100100010000001110100011010
00011001010110110100101010001000110010101000100110
01001001011011100010000000110010001100000011000100110111001011000010000001101
10101100001011011100011100100100000011001110110111101101111011001000010000001
11010001101000011010010110111001100111011100110010000001101000011000010111000
00111000001100101011011100110010101100100001011100010000001000010011101010111
01000010000001110111011001010010000001100100011010010110010000100000011011100
11011110111010000100000011010000110010101100001011100100010000001110100011010
00011001010110110100101010001000110010101000100110
01001001011011100010000000110010001100000011000100110111001011000010000001101
10101100001011011100101100100100000011001110110111101101111011001000010000001
11010001101000011010010110111001100111011100110010000001101000011000010111000
00111000001100101011011100110010101100100001011100010000001000010011101010111
01000010000001110111011001010010000001100100011010010110010000100000011011100
11011110111010000100000011010000110010101100001011100100010000001110100011010
00011001010110110100101010001000110010101000100110
01001001011011100010000000110010001100000011000100110111001011000010000001101
10101100001011011100110100100100000011001110110111101101111011001000010000001
11010001101000011010010110111001100111011100110010000001101000011000010111000
00111000001100101011011100110010101100100001011100010000001000010011101010111
01000010000001110111011001010010000001100100011010010110010000100000011011100
11011110111010000100000011010000110010101100001011100100010000001110100011010
00011001010110110100101010001000110010101000100110
01001001011011100010000000110010001100000011000100110111001011000010000001101
10101100001011011100111000100100000011001110110111101101111011001000010000001
11010001101000011010010110111001100111011100110010000001101000011000010111000
00111000001100101011011100110010101100100001011100010000001000010011101010111
01000010000001110111011001010010000001100100011010010110010000100000011011100
11011110111010000100000011010000110010101100001011100100010000001110100011010
00011001010110110100101010001000110010101000100110
01001001011011100010000000110010001100000011000100110111001011000010000001101
10101100001011011100111110100100000011001110110111101101111011001000010000001
11010001101000011010010110111001100111011100110010000001101000011000010111000
00111000001100101011011100110010101100100001011100010000001000010011101010111
01000010000001110111011001010010000001100100011010010110010000100000011011100
11011110111010000100000011010000110010101100001011100100010000001110100011010
00011001010110110100101010001000110010101000100110
01001001011011100010000000110010001100000011000100110111001011000010000001101
10101100001011011100111101100100000011001110110111101101111011001000010000001
11010001101000011010010110111001100111011100110010000001101000011000010111000
00111000001100101011011100110010101100100001011100010000001000010011101010111
01000010000001110111011001010010000001100100011010010110010000100000011011100
11011110111010000100000011010000110010101100001011100100010000001110100011010
00011001010110110100101010001000110010101000100110
01001001011011100010000000110010001100000011000100110111001011000010000001101
10101100001011011100111100000100000011001110110111101101111011001000010000001
11010001101000011010010110111001100111011100110010000001101000011000010111000
00111000001100101011011100110010101100100001011100010000001000010011101010111
01000010000001110111011001010010000001100100011010010110010000100000011011100

11011110111010000100000011010000110010101100001011100100010000001110100011010
00011001010110110100101010001000110010101000100110
01001001011011100010000000110010001100000011000100110111001011000010000001101
10101100001011011100111100110100000011001110110111101101111011001000010000001
11010001101000011010010110111001100111011100110010000001101000011000010111000
00111000001100101011011100110010101100100001011100010000001000010011101010111
01000010000001110111011001010010000001100100011010010110010000100000011011100
11011110111010000100000011010000110010101100001011100100010000001110100011010
00011001010110110100101010001000110010101000100110
01001001011011100010000000110010001100000011000100110111001011000010000001101
10101100001011011100111100101100000011001110110111101101111011001000010000001
11010001101000011010010110111001100111011100110010000001101000011000010111000
00111000001100101011011100110010101100100001011100010000001000010011101010111
01000010000001110111011001010010000001100100011010010110010000100000011011100
11011110111010000100000011010000110010101100001011100100010000001110100011010
00011001010110110100101010001000110010101000100110
01001001011011100010000000110010001100000011000100110111001011000010000001101
10101100001011011100111100100000000011001110110111101101111011001000010000001
11010001101000011010010110111001100111011100110010000001101000011000010111000
00111000001100101011011100110010101100100001011100010000001000010011101010111
01000010000001110111011001010010000001100100011010010110010000100000011011100
11011110111010000100000011010000110010101100001011100100010000001110100011010
00011001010110110100101010001000110010101000100110
01001001011011100010000000110010001100000011000100110111001011000010000001101
10101100001011011100111100100110000011001110110111101101111011001000010000001
11010001101000011010010110111001100111011100110010000001101000011000010111000
00111000001100101011011100110010101100100001011100010000001000010011101010111
01000010000001110111011001010010000001100100011010010110010000100000011011100
11011110111010000100000011010000110010101100001011100100010000001110100011010
00011001010110110100101010001000110010101000100110
01001001011011100010000000110010001100000011000100110111001011000010000001101
10101100001011011100111100100101000011001110110111101101111011001000010000001
11010001101000011010010110111001100111011100110010000001101000011000010111000
00111000001100101011011100110010101100100001011100010000001000010011101010111
01000010000001110111011001010010000001100100011010010110010000100000011011100
11011110111010000100000011010000110010101100001011100100010000001110100011010
00011001010110110100101010001000110010101000100110
01001001011011100010000000110010001100000011000100110111001011000010000001101
10101100001011011100111100100100100011001110110111101101111011001000010000001
11010001101000011010010110111001100111011100110010000001101000011000010111000
00111000001100101011011100110010101100100001011100010000001000010011101010111
01000010000001110111011001010010000001100100011010010110010000100000011011100
11011110111010000100000011010000110010101100001011100100010000001110100011010
00011001010110110100101010001000110010101000100110
01001001011011100010000000110010001100000011000100110111001011000010000001101
10101100001011011100111100100100010011001110110111101101111011001000010000001
11010001101000011010010110111001100111011100110010000001101000011000010111000
00111000001100101011011100110010101100100001011100010000001000010011101010111
01000010000001110111011001010010000001100100011010010110010000100000011011100
11011110111010000100000011010000110010101100001011100100010000001110100011010
00011001010110110100101010001000110010101000100110
01001001011011100010000000110010001100000011000100110111001011000010000001101
10101100001011011100111100100100001011001110110111101101111011001000010000001
11010001101000011010010110111001100111011100110010000001101000011000010111000
00111000001100101011011100110010101100100001011100010000001000010011101010111
01000010000001110111011001010010000001100100011010010110010000100000011011100

11011110111010000100000011010000110010101100001011100100010000001110100011010
00011001010110110100101010001000110010101000100110
01001001011011100010000000110010001100000011000100110111001011000010000001101
10101100001011011100111100100100000011100110110111101101111011001000010000001
11010001101000011010010110111001100111011100110010000001101000011000010111000
00111000001100101011011100110010101100100001011100010000001000010011101010111
01000010000001110111011001010010000001100100011010010110010000100000011011100
11011110111010000100000011010000110010101100001011100100010000001110100011010
00011001010110110100101010001000110010101000100110
01001001011011100010000000110010001100000011000100110111001011000010000001101
10101100001011011100111100100100000001001110110111101101111011001000010000001
11010001101000011010010110111001100111011100110010000001101000011000010111000
00111000001100101011011100110010101100100001011100010000001000010011101010111
01000010000001110111011001010010000001100100011010010110010000100000011011100
11011110111010000100000011010000110010101100001011100100010000001110100011010
00011001010110110100101010001000110010101000100110
01001001011011100010000000110010001100000011000100110111001011000010000001101
10101100001011011100111100100100000010001110110111101101111011001000010000001
11010001101000011010010110111001100111011100110010000001101000011000010111000
00111000001100101011011100110010101100100001011100010000001000010011101010111
01000010000001110111011001010010000001100100011010010110010000100000011011100
11011110111010000100000011010000110010101100001011100100010000001110100011010
00011001010110110100101010001000110010101000100110
01001001011011100010000000110010001100000011000100110111001011000010000001101
10101100001011011100111100100100000011101110110111101101111011001000010000001
11010001101000011010010110111001100111011100110010000001101000011000010111000
00111000001100101011011100110010101100100001011100010000001000010011101010111
01000010000001110111011001010010000001100100011010010110010000100000011011100
11011110111010000100000011010000110010101100001011100100010000001110100011010
00011001010110110100101010001000110010101000100110
01001001011011100010000000110010001100000011000100110111001011000010000001101
10101100001011011100111100100100000011011110110111101101111011001000010000001
11010001101000011010010110111001100111011100110010000001101000011000010111000
00111000001100101011011100110010101100100001011100010000001000010011101010111
01000010000001110111011001010010000001100100011010010110010000100000011011100
11011110111010000100000011010000110010101100001011100100010000001110100011010
00011001010110110100101010001000110010101000100110
01001001011011100010000000110010001100000011000100110111001011000010000001101
10101100001011011100111100100100000011000110110111101101111011001000010000001
11010001101000011010010110111001100111011100110010000001101000011000010111000
00111000001100101011011100110010101100100001011100010000001000010011101010111
01000010000001110111011001010010000001100100011010010110010000100000011011100
11011110111010000100000011010000110010101100001011100100010000001110100011010
00011001010110110100101010001000110010101000100110
01001001011011100010000000110010001100000011000100110111001011000010000001101
10101100001011011100111100100100000011001010110111101101111011001000010000001
11010001101000011010010110111001100111011100110010000001101000011000010111000
00111000001100101011011100110010101100100001011100010000001000010011101010111
01000010000001110111011001010010000001100100011010010110010000100000011011100
11011110111010000100000011010000110010101100001011100100010000001110100011010
00011001010110110100101010001000110010101000100110
01001001011011100010000000110010001100000011000100110111001011000010000001101
10101100001011011100111100100100000011001100110111101101111011001000010000001
11010001101000011010010110111001100111011100110010000001101000011000010111000
00111000001100101011011100110010101100100001011100010000001000010011101010111
01000010000001110111011001010010000001100100011010010110010000100000011011100

11011110111010000100000011010000110010101100001011100100010000001110100011010
00011001010110110100101010001000110010101000100110
01001001011011100010000000110010001100000011000100110111001011000010000001101
10101100001011011100111100100100000011001111110111101101111011001000010000001
11010001101000011010010110111001100111011100110010000001101000011000010111000
00111000001100101011011100110010101100100001011100010000000100001001110101011 1
01000010000000111011101100101001000000110010001101001011001000010000001101110 0
11011110111010000100000011010000110010101100001011100100010000001110100011010
00011001010110110100101010001000110010101000100110
01001001011011100010000000110010001100000011000100110111001011000010000001101
10101100001011011100111100100100000011001110010111101101111011001000010000001
11010001101000011010010110111001100111011100110010000001101000011000010111000
00111000001100101011011100110010101100100001011100010000000100001001110101011 1
01000010000000111011101100101001000000110010001101001011001000010000001101110 0
11011110111010000100000011010000110010101100001011100100010000001110100011010
00011001010110110100101010001000110010101000100110
01001001011011100010000000110010001100000011000100110111001011000010000001101
10101100001011011100111100100100000011001110100111101101111011001000010000001
11010001101000011010010110111001100111011100110010000001101000011000010111000
00111000001100101011011100110010101100100001011100010000000100001001110101011 1
01000010000000111011101100101001000000110010001101001011001000010000001101110 0
11011110111010000100000011010000110010101100001011100100010000001110100011010
00011001010110110100101010001000110010101000100110
01001001011011100010000000110010001100000011000100110111001011000010000001101
10101100001011011100111100100100000011001110111111101101111011001000010000001
11010001101000011010010110111001100111011100110010000001101000011000010111000
00111000001100101011011100110010101100100001011100010000000100001001110101011 1
01000010000000111011101100101001000000110010001101001011001000010000001101110 0
11011110111010000100000011010000110010101100001011100100010000001110100011010
00011001010110110100101010001000110010101000100110
01001001011011100010000000110010001100000011000100110111001011000010000001101
10101100001011011100111100100100000011001110110011101101111011001000010000001
11010001101000011010010110111001100111011100110010000001101000011000010111000
00111000001100101011011100110010101100100001011100010000000100001001110101011 1
01000010000000111011101100101001000000110010001101001011001000010000001101110 0
11011110111010000100000011010000110010101100001011100100010000001110100011010
00011001010110110100101010001000110010101000100110
01001001011011100010000000110010001100000011000100110111001011000010000001101
10101100001011011100111100100100000011001110110101101101111011001000010000001
11010001101000011010010110111001100111011100110010000001101000011000010111000
00111000001100101011011100110010101100100001011100010000000100001001110101011 1
01000010000000111011101100101001000000110010001101001011001000010000001101110 0
11011110111010000100000011010000110010101100001011100100010000001110100011010
00011001010110110100101010001000110010101000100110
01001001011011100010000000110010001100000011000100110111001011000010000001101
10101100001011011100111100100100000011001110110110101101111011001000010000001
11010001101000011010010110111001100111011100110010000001101000011000010111000
00111000001100101011011100110010101100100001011100010000000100001001110101011 1
01000010000000111011101100101001000000110010001101001011001000010000001101110 0
11011110111010000100000011010000110010101100001011100100010000001110100011010
00011001010110110100101010001000110010101000100110
01001001011011100010000000110010001100000011000100110111001011000010000001101
10101100001011011100111100100100000011001110110111001101111011001000010000001
11010001101000011010010110111001100111011100110010000001101000011000010111000
00111000001100101011011100110010101100100001011100010000000100001001110101011 1
01000010000000111011101100101001000000110010001101001011001000010000001101110 0

110111101110100001000000110100001100101011000010111001000100000011101000110101 0
00011001010110110100101010001000110010101000100110

File 2: “Track your record it is impossible to maintain your integrity12.”

10110010000001111001011011110111010101110010001000000111001001100101011000110
11011110111001001100100001000000110100101110100001000000110100101110011001000
00011010010110110101110000011011110111001101110011011010010110001001101100011
00101001000000111010001101111001000000110110101100001011010010110111001110100
01100001011010010110111000100000011110010110111101110101011100100010000001101
00101101110011101000110010101100111011100100110100101110100011110010011000100
11001000101110
01010110011100100110000101100011011010110010000001111001011011110111010101110
01000100000011100100110010101100011011011110111001001100100001000000110100101
11010000100000011010010111001100100000011010010110110101110000011011110111001
10111001101101001011000100110110001100101001000000111010001101111001000000110
11010110000101101001011011100111010001100001011010010110111000100000011110010
11011110111010101110010001000000110100101101110011101000110010101100111011100
10011010010111010001111001001100010011001000101110
01010101011100100110000101100011011010110010000001111001011011110111010101110
01000100000011100100110010101100011011011110111001001100100001000000110100101
11010000100000011010010111001100100000011010010110110101110000011011110111001
10111001101101001011000100110110001100101001000000111010001101111001000000110
11010110000101101001011011100111010001100001011010010110111000100000011110010
11011110111010101110010001000000110100101101110011101000110010101100111011100
10011010010111010001111001001100010011001000101110
01010100111100100110000101100011011010110010000001111001011011110111010101110
01000100000011100100110010101100011011011110111001001100100001000000110100101
11010000100000011010010111001100100000011010010110110101110000011011110111001
10111001101101001011000100110110001100101001000000111010001101111001000000110
11010110000101101001011011100111010001100001011010010110111000100000011110010
11011110111010101110010001000000110100101101110011101000110010101100111011100
10011010010111010001111001001100010011001000101110
01010100001100100110000101100011011010110010000001111001011011110111010101110
01000100000011100100110010101100011011011110111001001100100001000000110100101
11010000100000011010010111001100100000011010010110110101110000011011110111001
10111001101101001011000100110110001100101001000000111010001101111001000000110
11010110000101101001011011100111010001100001011010010110111000100000011110010
11011110111010101110010001000000110100101101110011101000110010101100111011100
10011010010111010001111001001100010011001000101110
01010100010100100110000101100011011010110010000001111001011011110111010101110
01000100000011100100110010101100011011011110111001001100100001000000110100101
11010000100000011010010111001100100000011010010110110101110000011011110111001
10111001101101001011000100110110001100101001000000111010001101111001000000110
11010110000101101001011011100111010001100001011010010110111000100000011110010
11011110111010101110010001000000110100101101110011101000110010101100111011100
10011010010111010001111001001100010011001000101110
01010100011000100110000101100011011010110010000001111001011011110111010101110
01000100000011100100110010101100011011011110111001001100100001000000110100101
11010000100000011010010111001100100000011010010110110101110000011011110111001
10111001101101001011000100110110001100101001000000111010001101111001000000110
11010110000101101001011011100111010001100001011010010110111000100000011110010
11011110111010101110010001000000110100101101110011101000110010101100111011100
10011010010111010001111001001100010011001000101110
01010100011110100110000101100011011010110010000001111001011011110111010101110
01000100000011100100110010101100011011011110111001001100100001000000110100101

110100001000000011010010111001100100000011010010110110101110000011011110111001
10111001101101001011000100110110001100101001000000111010001101111001000000110
11010110000101101001011011100111010001100001011010010110111000100000011110010
11011110111010101110010001000000110100101101110011101000110010101100111011100
10011010010111010001111001001100010011001000101110
01010100011101100110000101100011011010110010000001111001011011110111010101110
0100010000000111001001100101011000110110111101110010011001000010000000110100101
110100001000000011010010111001100100000011010010110110101110000011011110111001
10111001101101001011000100110110001100101001000000111010001101111001000000110
11010110000101101001011011100111010001100001011010010110111000100000011110010
11011110111010101110010001000000110100101101110011101000110010101100111011100
10011010010111010001111001001100010011001000101110
01010100011100000110000101100011011010110010000001111001011011110111010101110
0100010000000111001001100101011000110110111101110010011001000010000000110100101
110100001000000011010010111001100100000011010010110110101110000011011110111001
10111001101101001011000100110110001100101001000000111010001101111001000000110
11010110000101101001011011100111010001100001011010010110111000100000011110010
11011110111010101110010001000000110100101101110011101000110010101100111011100
10011010010111010001111001001100010011001000101110
01010100011100110110000101100011011010110010000001111001011011110111010101110
0100010000000111001001100101011000110110111101110010011001000010000000110100101
110100001000000011010010111001100100000011010010110110101110000011011110111001
10111001101101001011000100110110001100101001000000111010001101111001000000110
11010110000101101001011011100111010001100001011010010110111000100000011110010
11011110111010101110010001000000110100101101110011101000110010101100111011100
10011010010111010001111001001100010011001000101110
01010100011100101110000101100011011010110010000001111001011011110111010101110
0100010000000111001001100101011000110110111101110010011001000010000000110100101
110100001000000011010010111001100100000011010010110110101110000011011110111001
10111001101101001011000100110110001100101001000000111010001101111001000000110
11010110000101101001011011100111010001100001011010010110111000100000011110010
11011110111010101110010001000000110100101101110011101000110010101100111011100
10011010010111010001111001001100010011001000101110
01010100011100100010000101100011011010110010000001111001011011110111010101110
0100010000000111001001100101011000110110111101110010011001000010000000110100101
110100001000000011010010111001100100000011010010110110101110000011011110111001
10111001101101001011000100110110001100101001000000111010001101111001000000110
11010110000101101001011011100111010001100001011010010110111000100000011110010
11011110111010101110010001000000110100101101110011101000110010101100111011100
10011010010111010001111001001100010011001000101110
01010100011100100100000101100011011010110010000001111001011011110111010101110
0100010000000111001001100101011000110110111101110010011001000010000000110100101
110100001000000011010010111001100100000011010010110110101110000011011110111001
10111001101101001011000100110110001100101001000000111010001101111001000000110
11010110000101101001011011100111010001100001011010010110111000100000011110010
11011110111010101110010001000000110100101101110011101000110010101100111011100
10011010010111010001111001001100010011001000101110
01010100011100100111000101100011011010110010000001111001011011110111010101110
0100010000000111001001100101011000110110111101110010011001000010000000110100101
110100001000000011010010111001100100000011010010110110101110000011011110111001
10111001101101001011000100110110001100101001000000111010001101111001000000110
11010110000101101001011011100111010001100001011010010110111000100000011110010
11011110111010101110010001000000110100101101110011101000110010101100111011100
10011010010111010001111001001100010011001000101110
01010100011100100110100101100011011010110010000001111001011011110111010101110
0100010000000111001001100101011000110110111101110010011001000010000000110100101
110100001000000011010010111001100100000011010010110110101110000011011110111001

1011100110110100101100010011011000110010100100000011101000110111100100000110
1101011000010110100101101110011101000110000101101001011011100010000000111110010
1101111011101010111001000100000001101001011011100111010001100101011001110111100
100110100101110100011110010011000100110010000101110
0101010001110010011001010110001101101011001000000111100101101111011101010101110
0100010000000111001001100101011000110110111101110010011001000010000000110100101
1101000010000000110100101110011001000000011010010110110101110000011011110111001
1011100110110100101100010011011000110010100100000011101000110111100100000110
1101011000010110100101101110011101000110000101101001011011100010000000111110010
1101111011101010111001000100000001101001011011100111010001100101011001110111100
100110100101110100011110010011000100110010000101110
0101010001110010011000110110001101101011001000000111100101101111011101010101110
0100010000000111001001100101011000110110111101110010011001000010000000110100101
1101000010000000110100101110011001000000011010010110110101110000011011110111001
1011100110110100101100010011011000110010100100000011101000110111100100000110
1101011000010110100101101110011101000110000101101001011011100010000000111110010
1101111011101010111001000100000001101001011011100111010001100101011001110111100
100110100101110100011110010011000100110010000101110
0101010001110010011000000110001101101011001000000111100101101111011101010101110
0100010000000111001001100101011000110110111101110010011001000010000000110100101
1101000010000000110100101110011001000000011010010110110101110000011011110111001
1011100110110100101100010011011000110010100100000011101000110111100100000110
1101011000010110100101101110011101000110000101101001011011100010000000111110010
1101111011101010111001000100000001101001011011100111010001100101011001110111100
100110100101110100011110010011000100110010000101110
0101010001110010011000011110001101101011001000000111100101101111011101010101110
0100010000000111001001100101011000110110111101110010011001000010000000110100101
1101000010000000110100101110011001000000011010010110110101110000011011110111001
1011100110110100101100010011011000110010100100000011101000110111100100000110
1101011000010110100101101110011101000110000101101001011011100010000000111110010
1101111011101010111001000100000001101001011011100111010001100101011001110111100
100110100101110100011110010011000100110010000101110
0101010001110010011000010010001101101011001000000111100101101111011101010101110
0100010000000111001001100101011000110110111101110010011001000010000000110100101
1101000010000000110100101110011001000000011010010110110101110000011011110111001
1011100110110100101100010011011000110010100100000011101000110111100100000110
1101011000010110100101101110011101000110000101101001011011100010000000111110010
1101111011101010111001000100000001101001011011100111010001100101011001110111100
100110100101110100011110010011000100110010000101110
0101010001110010011000010100001101101011001000000111100101101111011101010101110
0100010000000111001001100101011000110110111101110010011001000010000000110100101
1101000010000000110100101110011001000000011010010110110101110000011011110111001
1011100110110100101100010011011000110010100100000011101000110111100100000110
1101011000010110100101101110011101000110000101101001011011100010000000111110010
1101111011101010111001000100000001101001011011100111010001100101011001110111100
100110100101110100011110010011000100110010000101110
0101010001110010011000010111001101101011001000000111100101101111011101010101110
0100010000000111001001100101011000110110111101110010011001000010000000110100101
1101000010000000110100101110011001000000011010010110110101110000011011110111001
1011100110110100101100010011011000110010100100000011101000110111100100000110
1101011000010110100101101110011101000110000101101001011011100010000000111110010
1101111011101010111001000100000001101001011011100111010001100101011001110111100
100110100101110100011110010011000100110010000101110
0101010001110010011000010110101101101011001000000111100101101111011101010101110
0100010000000111001001100101011000110110111101110010011001000010000000110100101
1101000010000000110100101110011001000000011010010110110101110000011011110111001
1011100110110100101100010011011000110010100100000011101000110111100100000110

11010110000101101001011011100111010001100001011010010110111000100000011110010
11011110111010101110010001000000110100101101110011101000110010101100111011100
10011010010111010001111001001100010011001000101110
01010100011100100110000101100111011010110010000001111001011011110111010101110
01000100000011100100110010101100011011011110111001001100100001000000110100101
11010000100000011010010111001100100000011010010110110101110000011011110111001
10111001101101001011000100110110001100101001000000111010001101111001000000110
11010110000101101001011011100111010001100001011010010110111000100000011110010
11011110111010101110010001000000110100101101110011101000110010101100111011100
10011010010111010001111001001100010011001000101110
01010100011100100110000101100001011010110010000001111001011011110111010101110
01000100000011100100110010101100011011011110111001001100100001000000110100101
11010000100000011010010111001100100000011010010110110101110000011011110111001
10111001101101001011000100110110001100101001000000111010001101111001000000110
11010110000101101001011011100111010001100001011010010110111000100000011110010
11011110111010101110010001000000110100101101110011101000110010101100111011100
10011010010111010001111001001100010011001000101110
01010100011100100110000101100010011010110010000001111001011011110111010101110
01000100000011100100110010101100011011011110111001001100100001000000110100101
11010000100000011010010111001100100000011010010110110101110000011011110111001
10111001101101001011000100110110001100101001000000111010001101111001000000110
11010110000101101001011011100111010001100001011010010110111000100000011110010
11011110111010101110010001000000110100101101110011101000110010101100111011100
10011010010111010001111001001100010011001000101110
01010100011100100110000101100011111010110010000001111001011011110111010101110
01000100000011100100110010101100011011011110111001001100100001000000110100101
11010000100000011010010111001100100000011010010110110101110000011011110111001
10111001101101001011000100110110001100101001000000111010001101111001000000110
11010110000101101001011011100111010001100001011010010110111000100000011110010
11011110111010101110010001000000110100101101110011101000110010101100111011100
10011010010111010001111001001100010011001000101110
01010100011100100110000101100011001010110010000001111001011011110111010101110
01000100000011100100110010101100011011011110111001001100100001000000110100101
11010000100000011010010111001100100000011010010110110101110000011011110111001
10111001101101001011000100110110001100101001000000111010001101111001000000110
11010110000101101001011011100111010001100001011010010110111000100000011110010
11011110111010101110010001000000110100101101110011101000110010101100111011100
10011010010111010001111001001100010011001000101110
01010100011100100110000101100011010010110010000001111001011011110111010101110
01000100000011100100110010101100011011011110111001001100100001000000110100101
11010000100000011010010111001100100000011010010110110101110000011011110111001
10111001101101001011000100110110001100101001000000111010001101111001000000110
11010110000101101001011011100111010001100001011010010110111000100000011110010
11011110111010101110010001000000110100101101110011101000110010101100111011100
10011010010111010001111001001100010011001000101110
01010100011100100110000101100011011110110010000001111001011011110111010101110
01000100000011100100110010101100011011011110111001001100100001000000110100101
11010000100000011010010111001100100000011010010110110101110000011011110111001
10111001101101001011000100110110001100101001000000111010001101111001000000110
11010110000101101001011011100111010001100001011010010110111000100000011110010
11011110111010101110010001000000110100101101110011101000110010101100111011100
10011010010111010001111001001100010011001000101110
01010100011100100110000101100011011000110010000001111001011011110111010101110
01000100000011100100110010101100011011011110111001001100100001000000110100101
11010000100000011010010111001100100000011010010110110101110000011011110111001
10111001101101001011000100110110001100101001000000111010001101111001000000110
11010110000101101001011011100111010001100001011010010110111000100000011110010

11011110111010101110010001000000110100101101110011101000110010101100111011100
10011010010111010001111001001100010011001000101110
01010100011100100110000101100011011011110010000001111001011011110111010101110
01000100000011100100110010101100011011011110111001001100100001000000110100101
11010000100000011010010111001100100000011010010110110101110000011011110111001
10111001101101001011000100110110001100101001000000111010001101111001000000110
11010110000101101001011011100111010001100001011010010110111000100000011110010
11011110111010101110010001000000110100101101110011101000110010101100111011100
10011010010111010001111001001100010011001000101110
01010100011100100110000101100011011010010010000001111001011011110111010101110
01000100000011100100110010101100011011011110111001001100100001000000110100101
11010000100000011010010111001100100000011010010110110101110000011011110111001
10111001101101001011000100110110001100101001000000111010001101111001000000110
11010110000101101001011011100111010001100001011010010110111000100000011110010
11011110111010101110010001000000110100101101110011101000110010101100111011100
10011010010111010001111001001100010011001000101110
01010100011100100110000101100011011010100010000001111001011011110111010101110
01000100000011100100110010101100011011011110111001001100100001000000110100101
11010000100000011010010111001100100000011010010110110101110000011011110111001
10111001101101001011000100110110001100101001000000111010001101111001000000110
11010110000101101001011011100111010001100001011010010110111000100000011110010
11011110111010101110010001000000110100101101110011101000110010101100111011100
10011010010111010001111001001100010011001000101110
01010100011100100110000101100011011010111010000001111001011011110111010101110
01000100000011100100110010101100011011011110111001001100100001000000110100101
11010000100000011010010111001100100000011010010110110101110000011011110111001
10111001101101001011000100110110001100101001000000111010001101111001000000110
11010110000101101001011011100111010001100001011010010110111000100000011110010
11011110111010101110010001000000110100101101110011101000110010101100111011100
10011010010111010001111001001100010011001000101110
01010100011100100110000101100011011010110110000001111001011011110111010101110
01000100000011100100110010101100011011011110111001001100100001000000110100101
11010000100000011010010111001100100000011010010110110101110000011011110111001
10111001101101001011000100110110001100101001000000111010001101111001000000110
11010110000101101001011011100111010001100001011010010110111000100000011110010
11011110111010101110010001000000110100101101110011101000110010101100111011100
10011010010111010001111001001100010011001000101110
01010100011100100110000101100011011010110000000001111001011011110111010101110
01000100000011100100110010101100011011011110111001001100100001000000110100101
11010000100000011010010111001100100000011010010110110101110000011011110111001
10111001101101001011000100110110001100101001000000111010001101111001000000110
11010110000101101001011011100111010001100001011010010110111000100000011110010
11011110111010101110010001000000110100101101110011101000110010101100111011100
10011010010111010001111001001100010011001000101110
01010100011100100110000101100011011010110011000001111001011011110111010101110
01000100000011100100110010101100011011011110111001001100100001000000110100101
11010000100000011010010111001100100000011010010110110101110000011011110111001
10111001101101001011000100110110001100101001000000111010001101111001000000110
11010110000101101001011011100111010001100001011010010110111000100000011110010
11011110111010101110010001000000110100101101110011101000110010101100111011100
10011010010111010001111001001100010011001000101110
01010100011100100110000101100011011010110010100001111001011011110111010101110
01000100000011100100110010101100011011011110111001001100100001000000110100101
11010000100000011010010111001100100000011010010110110101110000011011110111001
10111001101101001011000100110110001100101001000000111010001101111001000000110
11010110000101101001011011100111010001100001011010010110111000100000011110010

11011110111010101110010001000000110100101101110011101000110010101100111011100
10011010010111010001111001001100010011001000101110
01010100011100100110000101100011011010110010010001111001011011110111010101110
01000100000011100100110010101100011011011110111001001100100001000000110100101
11010000100000011010010111001100100000011010010110110101110000011011110111001
10111001101101001011000100110110001100101001000000111010001101111001000000110
11010110000101101001011011100111010001100001011010010110111000100000011110010
11011110111010101110010001000000110100101101110011101000110010101100111011100
10011010010111010001111001001100010011001000101110
01010100011100100110000101100011011010110010001001111001011011110111010101110
01000100000011100100110010101100011011011110111001001100100001000000110100101
11010000100000011010010111001100100000011010010110110101110000011011110111001
10111001101101001011000100110110001100101001000000111010001101111001000000110
11010110000101101001011011100111010001100001011010010110111000100000011110010
11011110111010101110010001000000110100101101110011101000110010101100111011100
10011010010111010001111001001100010011001000101110
01010100011100100110000101100011011010110010000101111001011011110111010101110
01000100000011100100110010101100011011011110111001001100100001000000110100101
11010000100000011010010111001100100000011010010110110101110000011011110111001
10111001101101001011000100110110001100101001000000111010001101111001000000110
11010110000101101001011011100111010001100001011010010110111000100000011110010
11011110111010101110010001000000110100101101110011101000110010101100111011100
10011010010111010001111001001100010011001000101110
01010100011100100110000101100011011010110010000011111001011011110111010101110
01000100000011100100110010101100011011011110111001001100100001000000110100101
11010000100000011010010111001100100000011010010110110101110000011011110111001
10111001101101001011000100110110001100101001000000111010001101111001000000110
11010110000101101001011011100111010001100001011010010110111000100000011110010
11011110111010101110010001000000110100101101110011101000110010101100111011100
10011010010111010001111001001100010011001000101110
01010100011100100110000101100011011010110010000000111001011011110111010101110
01000100000011100100110010101100011011011110111001001100100001000000110100101
11010000100000011010010111001100100000011010010110110101110000011011110111001
10111001101101001011000100110110001100101001000000111010001101111001000000110
11010110000101101001011011100111010001100001011010010110111000100000011110010
11011110111010101110010001000000110100101101110011101000110010101100111011100
10011010010111010001111001001100010011001000101110
01010100011100100110000101100011011010110010000001011001011011110111010101110
01000100000011100100110010101100011011011110111001001100100001000000110100101
11010000100000011010010111001100100000011010010110110101110000011011110111001
10111001101101001011000100110110001100101001000000111010001101111001000000110
11010110000101101001011011100111010001100001011010010110111000100000011110010
11011110111010101110010001000000110100101101110011101000110010101100111011100
10011010010111010001111001001100010011001000101110
01010100011100100110000101100011011010110010000001101001011011110111010101110
01000100000011100100110010101100011011011110111001001100100001000000110100101
11010000100000011010010111001100100000011010010110110101110000011011110111001
10111001101101001011000100110110001100101001000000111010001101111001000000110
11010110000101101001011011100111010001100001011010010110111000100000011110010
11011110111010101110010001000000110100101101110011101000110010101100111011100
10011010010111010001111001001100010011001000101110
01010100011100100110000101100011011010110010000001110001011011110111010101110
01000100000011100100110010101100011011011110111001001100100001000000110100101
11010000100000011010010111001100100000011010010110110101110000011011110111001
10111001101101001011000100110110001100101001000000111010001101111001000000110
11010110000101101001011011100111010001100001011010010110111000100000011110010

11011110111010101110010001000000110100101101110011101000110010101100111011100
10011010010111010001111001001100010011001000101110
01010100011100100110000101100011011010110010000001111101011011110111010101110
01000100000011100100110010101100011011011110111001001100100001000000110100101
11010000100000011010010111001100100000011010010110110101110000011011110111001
10111001101101001011000100110110001100101001000000111010001101111001000000110
11010110000101101001011011100111010001100001011010010110111000100000011110010
11011110111010101110010001000000110100101101110011101000110010101100111011100
10011010010111010001111001001100010011001000101110
01010100011100100110000101100011011010110010000001111011011011110111010101110
01000100000011100100110010101100011011011110111001001100100001000000110100101
11010000100000011010010111001100100000011010010110110101110000011011110111001
10111001101101001011000100110110001100101001000000111010001101111001000000110
11010110000101101001011011100111010001100001011010010110111000100000011110010
11011110111010101110010001000000110100101101110011101000110010101100111011100
10011010010111010001111001001100010011001000101110
01010100011100100110000101100011011010110010000001111000011011110111010101110
01000100000011100100110010101100011011011110111001001100100001000000110100101
11010000100000011010010111001100100000011010010110110101110000011011110111001
10111001101101001011000100110110001100101001000000111010001101111001000000110
11010110000101101001011011100111010001100001011010010110111000100000011110010
11011110111010101110010001000000110100101101110011101000110010101100111011100
10011010010111010001111001001100010011001000101110
01010100011100100110000101100011011010110010000001111001111011110111010101110
01000100000011100100110010101100011011011110111001001100100001000000110100101
11010000100000011010010111001100100000011010010110110101110000011011110111001
10111001101101001011000100110110001100101001000000111010001101111001000000110
11010110000101101001011011100111010001100001011010010110111000100000011110010
11011110111010101110010001000000110100101101110011101000110010101100111011100
10011010010111010001111001001100010011001000101110
01010100011100100110000101100011011010110010000001111001001011110111010101110
01000100000011100100110010101100011011011110111001001100100001000000110100101
11010000100000011010010111001100100000011010010110110101110000011011110111001
10111001101101001011000100110110001100101001000000111010001101111001000000110
11010110000101101001011011100111010001100001011010010110111000100000011110010
11011110111010101110010001000000110100101101110011101000110010101100111011100
10011010010111010001111001001100010011001000101110
01010100011100100110000101100011011010110010000001111001010011110111010101110
01000100000011100100110010101100011011011110111001001100100001000000110100101
11010000100000011010010111001100100000011010010110110101110000011011110111001
10111001101101001011000100110110001100101001000000111010001101111001000000110
11010110000101101001011011100111010001100001011010010110111000100000011110010
11011110111010101110010001000000110100101101110011101000110010101100111011100
10011010010111010001111001001100010011001000101110
01010100011100100110000101100011011010110010000001111001011111110111010101110
01000100000011100100110010101100011011011110111001001100100001000000110100101
11010000100000011010010111001100100000011010010110110101110000011011110111001
10111001101101001011000100110110001100101001000000111010001101111001000000110
11010110000101101001011011100111010001100001011010010110111000100000011110010
11011110111010101110010001000000110100101101110011101000110010101100111011100
10011010010111010001111001001100010011001000101110
01010100011100100110000101100011011010110010000001111001011001110111010101110
01000100000011100100110010101100011011011110111001001100100001000000110100101
11010000100000011010010111001100100000011010010110110101110000011011110111001
10111001101101001011000100110110001100101001000000111010001101111001000000110
11010110000101101001011011100111010001100001011010010110111000100000011110010

11011110111010101110010001000000110100101101110011101000110010101100111011100
10011010010111010001111001001100010011001000101110
01010100011100100110000101100011011010110010000001111001011010110111010101110
01000100000011100100110010101100011011011110111001001100100001000000110100101
11010000100000011010010111001100100000011010010110110101110000011011110111001
10111001101101001011000100110110001100101001000000111010001101111001000000110
11010110000101101001011011100111010001100001011010010110111000100000011110010
11011110111010101110010001000000110100101101110011101000110010101100111011100
10011010010111010001111001001100010011001000101110
01010100011100100110000101100011011010110010000001111001011011010111010101110
01000100000011100100110010101100011011011110111001001100100001000000110100101
11010000100000011010010111001100100000011010010110110101110000011011110111001
10111001101101001011000100110110001100101001000000111010001101111001000000110
11010110000101101001011011100111010001100001011010010110111000100000011110010
11011110111010101110010001000000110100101101110011101000110010101100111011100
10011010010111010001111001001100010011001000101110
01010100011100100110000101100011011010110010000001111001011011100111010101110
01000100000011100100110010101100011011011110111001001100100001000000110100101
11010000100000011010010111001100100000011010010110110101110000011011110111001
10111001101101001011000100110110001100101001000000111010001101111001000000110
11010110000101101001011011100111010001100001011010010110111000100000011110010
11011110111010101110010001000000110100101101110011101000110010101100111011100
10011010010111010001111001001100010011001000101110
01010100011100100110000101100011011010110010000001111001011011111111010101110
01000100000011100100110010101100011011011110111001001100100001000000110100101
11010000100000011010010111001100100000011010010110110101110000011011110111001
10111001101101001011000100110110001100101001000000111010001101111001000000110
11010110000101101001011011100111010001100001011010010110111000100000011110010
11011110111010101110010001000000110100101101110011101000110010101100111011100
10011010010111010001111001001100010011001000101110
01010100011100100110000101100011011010110010000001111001011011110011010101110
01000100000011100100110010101100011011011110111001001100100001000000110100101
11010000100000011010010111001100100000011010010110110101110000011011110111001
10111001101101001011000100110110001100101001000000111010001101111001000000110
11010110000101101001011011100111010001100001011010010110111000100000011110010
11011110111010101110010001000000110100101101110011101000110010101100111011100
10011010010111010001111001001100010011001000101110
01010100011100100110000101100011011010110010000001111001011011110101010101110
01000100000011100100110010101100011011011110111001001100100001000000110100101
11010000100000011010010111001100100000011010010110110101110000011011110111001
10111001101101001011000100110110001100101001000000111010001101111001000000110
11010110000101101001011011100111010001100001011010010110111000100000011110010
11011110111010101110010001000000110100101101110011101000110010101100111011100
10011010010111010001111001001100010011001000101110
01010100011100100110000101100011011010110010000001111001011011110110010101110
01000100000011100100110010101100011011011110111001001100100001000000110100101
11010000100000011010010111001100100000011010010110110101110000011011110111001
10111001101101001011000100110110001100101001000000111010001101111001000000110
11010110000101101001011011100111010001100001011010010110111000100000011110010
11011110111010101110010001000000110100101101110011101000110010101100111011100
10011010010111010001111001001100010011001000101110
01010100011100100110000101100011011010110010000001111001011011110111110101110
01000100000011100100110010101100011011011110111001001100100001000000110100101
11010000100000011010010111001100100000011010010110110101110000011011110111001
10111001101101001011000100110110001100101001000000111010001101111001000000110
11010110000101101001011011100111010001100001011010010110111000100000011110010

11011110111010101110010001000000110100101101110011101000110010101100111011100
10011010010111010001111001001100010011001000101110
01010100011100100110000101100011011010110010000001111001011011110111000101110
01000100000001110010011001010110001101101111011100100110010000100000001101001 01
11010000100000011010010111001100100000011010010110110101110000001101111011 1001
10111001101101001011000100110110001100101001000000111010001101111001000000110
11010110000101101001011011100111010001100001011010010110111000100000011110010
11011110111010101110010001000000110100101101110011101000110010101100111011100
10011010010111010001111001001100010011001000101110
01010100011100100110000101100011011010110010000001111001011011110111011101110
01000100000001110010011001010110001101101111011100100110010000100000001101001 01
11010000100000011010010111001100100000011010010110110101110000001101111011 1001
10111001101101001011000100110110001100101001000000111010001101111001000000110
11010110000101101001011011100111010001100001011010010110111000100000011110010
11011110111010101110010001000000110100101101110011101000110010101100111011100
10011010010111010001111001001100010011001000101110
01010100011100100110000101100011011010110010000001111001011011110111010001110
01000100000001110010011001010110001101101111011100100110010000100000001101001 01
11010000100000011010010111001100100000011010010110110101110000001101111011 1001
10111001101101001011000100110110001100101001000000111010001101111001000000110
11010110000101101001011011100111010001100001011010010110111000100000011110010
11011110111010101110010001000000110100101101110011101000110010101100111011100
10011010010111010001111001001100010011001000101110
01010100011100100110000101100011011010110010000001111001011011110111010111110
01000100000001110010011001010110001101101111011100100110010000100000001101001 01
11010000100000011010010111001100100000011010010110110101110000001101111011 1001
10111001101101001011000100110110001100101001000000111010001101111001000000110
11010110000101101001011011100111010001100001011010010110111000100000011110010
11011110111010101110010001000000110100101101110011101000110010101100111011100
10011010010111010001111001001100010011001000101110
01010100011100100110000101100011011010110010000001111001011011110111010100110
01000100000001110010011001010110001101101111011100100110010000100000001101001 01
11010000100000011010010111001100100000011010010110110101110000001101111011 1001
10111001101101001011000100110110001100101001000000111010001101111001000000110
11010110000101101001011011100111010001100001011010010110111000100000011110010
11011110111010101110010001000000110100101101110011101000110010101100111011100
10011010010111010001111001001100010011001000101110
01010100011100100110000101100011011010110010000001111001011011110111010101010
01000100000001110010011001010110001101101111011100100110010000100000001101001 01
11010000100000011010010111001100100000011010010110110101110000001101111011 1001
10111001101101001011000100110110001100101001000000111010001101111001000000110
11010110000101101001011011100111010001100001011010010110111000100000011110010
11011110111010101110010001000000110100101101110011101000110010101100111011100
10011010010111010001111001001100010011001000101110
01010100011100100110000101100011011010110010000001111001011011110111010101100
01000100000001110010011001010110001101101111011100100110010000100000001101001 01
11010000100000011010010111001100100000011010010110110101110000001101111011 1001
10111001101101001011000100110110001100101001000000111010001101111001000000110
11010110000101101001011011100111010001100001011010010110111000100000011110010
11011110111010101110010001000000110100101101110011101000110010101100111011100
10011010010111010001111001001100010011001000101110
01010100011100100110000101100011011010110010000001111001011011110111010101111
01000100000001110010011001010110001101101111011100100110010000100000001101001 01
11010000100000011010010111001100100000011010010110110101110000001101111011 1001
10111001101101001011000100110110001100101001000000111010001101111001000000110
11010110000101101001011011100111010001100001011010010110111000100000011110010

11011110111010101110010001000000110100101101110011101000110010101100111011100
10011010010111010001111001001100010011001000101110
01010100011100100110000101100011011010110010000001111001011011110111010101110
11000100000011100100110010101100011011011110111001001100100001000000110100101
11010000100000011010010111001100100000011010010110110101110000011011110111001
10111001101101001011000100110110001100101001000000111010001101111001000000110
11010110000101101001011011100111010001100001011010010110111000100000011110010
11011110111010101110010001000000110100101101110011101000110010101100111011100
10011010010111010001111001001100010011001000101110
01010100011100100110000101100011011010110010000001111001011011110111010101110
00000100000011100100110010101100011011011110111001001100100001000000110100101
11010000100000011010010111001100100000011010010110110101110000011011110111001
10111001101101001011000100110110001100101001000000111010001101111001000000110
11010110000101101001011011100111010001100001011010010110111000100000011110010
11011110111010101110010001000000110100101101110011101000110010101100111011100
10011010010111010001111001001100010011001000101110
01010100011100100110000101100011011010110010000001111001011011110111010101110
01100100000011100100110010101100011011011110111001001100100001000000110100101
11010000100000011010010111001100100000011010010110110101110000011011110111001
10111001101101001011000100110110001100101001000000111010001101111001000000110
11010110000101101001011011100111010001100001011010010110111000100000011110010
11011110111010101110010001000000110100101101110011101000110010101100111011100
10011010010111010001111001001100010011001000101110
01010100011100100110000101100011011010110010000001111001011011110111010101110
01010100000011100100110010101100011011011110111001001100100001000000110100101
11010000100000011010010111001100100000011010010110110101110000011011110111001
10111001101101001011000100110110001100101001000000111010001101111001000000110
11010110000101101001011011100111010001100001011010010110111000100000011110010
11011110111010101110010001000000110100101101110011101000110010101100111011100
10011010010111010001111001001100010011001000101110
01010100011100100110000101100011011010110010000001111001011011110111010101110
01001100000011100100110010101100011011011110111001001100100001000000110100101
11010000100000011010010111001100100000011010010110110101110000011011110111001
10111001101101001011000100110110001100101001000000111010001101111001000000110
11010110000101101001011011100111010001100001011010010110111000100000011110010
11011110111010101110010001000000110100101101110011101000110010101100111011100
10011010010111010001111001001100010011001000101110
01010100011100100110000101100011011010110010000001111001011011110111010101110
01000000000011100100110010101100011011011110111001001100100001000000110100101
11010000100000011010010111001100100000011010010110110101110000011011110111001
10111001101101001011000100110110001100101001000000111010001101111001000000110
11010110000101101001011011100111010001100001011010010110111000100000011110010
11011110111010101110010001000000110100101101110011101000110010101100111011100
10011010010111010001111001001100010011001000101110
01010100011100100110000101100011011010110010000001111001011011110111010101110
01000110000011100100110010101100011011011110111001001100100001000000110100101
11010000100000011010010111001100100000011010010110110101110000011011110111001
10111001101101001011000100110110001100101001000000111010001101111001000000110
11010110000101101001011011100111010001100001011010010110111000100000011110010
11011110111010101110010001000000110100101101110011101000110010101100111011100
10011010010111010001111001001100010011001000101110
01010100011100100110000101100011011010110010000001111001011011110111010101110
01000101000011100100110010101100011011011110111001001100100001000000110100101
11010000100000011010010111001100100000011010010110110101110000011011110111001
10111001101101001011000100110110001100101001000000111010001101111001000000110
11010110000101101001011011100111010001100001011010010110111000100000011110010

11011110111010101110010001000000110100101101110011101000110010101100111011100
10011010010111010001111001001100010011001000101110
01010100011100100110000101100011011010110010000001111001011011110111010101110
01000100100011100100110010101100011011011110111001001100100001000000110100101
11010000100000011010010111001100100000011010010110110101110000011011110111001
10111001101101001011000100110110001100101001000000111010001101111001000000110
11010110000101101001011011100111010001100001011010010110111000100000011110010
11011110111010101110010001000000110100101101110011101000110010101100111011100
10011010010111010001111001001100010011001000101110
01010100011100100110000101100011011010110010000001111001011011110111010101110
01000100010011100100110010101100011011011110111001001100100001000000110100101
11010000100000011010010111001100100000011010010110110101110000011011110111001
10111001101101001011000100110110001100101001000000111010001101111001000000110
11010110000101101001011011100111010001100001011010010110111000100000011110010
11011110111010101110010001000000110100101101110011101000110010101100111011100
10011010010111010001111001001100010011001000101110
01010100011100100110000101100011011010110010000001111001011011110111010101110
01000100001011100100110010101100011011011110111001001100100001000000110100101
11010000100000011010010111001100100000011010010110110101110000011011110111001
10111001101101001011000100110110001100101001000000111010001101111001000000110
11010110000101101001011011100111010001100001011010010110111000100000011110010
11011110111010101110010001000000110100101101110011101000110010101100111011100
10011010010111010001111001001100010011001000101110
01010100011100100110000101100011011010110010000001111001011011110111010101110
01000100000111100100110010101100011011011110111001001100100001000000110100101
11010000100000011010010111001100100000011010010110110101110000011011110111001
10111001101101001011000100110110001100101001000000111010001101111001000000110
11010110000101101001011011100111010001100001011010010110111000100000011110010
11011110111010101110010001000000110100101101110011101000110010101100111011100
10011010010111010001111001001100010011001000101110
01010100011100100110000101100011011010110010000001111001011011110111010101110
01000100000001100100110010101100011011011110111001001100100001000000110100101
11010000100000011010010111001100100000011010010110110101110000011011110111001
10111001101101001011000100110110001100101001000000111010001101111001000000110
11010110000101101001011011100111010001100001011010010110111000100000011110010
11011110111010101110010001000000110100101101110011101000110010101100111011100
10011010010111010001111001001100010011001000101110
01010100011100100110000101100011011010110010000001111001011011110111010101110
01000100000010100100110010101100011011011110111001001100100001000000110100101
11010000100000011010010111001100100000011010010110110101110000011011110111001
10111001101101001011000100110110001100101001000000111010001101111001000000110
11010110000101101001011011100111010001100001011010010110111000100000011110010
11011110111010101110010001000000110100101101110011101000110010101100111011100
10011010010111010001111001001100010011001000101110
01010100011100100110000101100011011010110010000001111001011011110111010101110
01000100000011000100110010101100011011011110111001001100100001000000110100101
11010000100000011010010111001100100000011010010110110101110000011011110111001
10111001101101001011000100110110001100101001000000111010001101111001000000110
11010110000101101001011011100111010001100001011010010110111000100000011110010
11011110111010101110010001000000110100101101110011101000110010101100111011100
10011010010111010001111001001100010011001000101110
01010100011100100110000101100011011010110010000001111001011011110111010101110
01000100000011110100110010101100011011011110111001001100100001000000110100101
11010000100000011010010111001100100000011010010110110101110000011011110111001
10111001101101001011000100110110001100101001000000111010001101111001000000110
11010110000101101001011011100111010001100001011010010110111000100000011110010

11011110111010101110010001000000110100101101110011101000110010101100111011100
10011010010111010001111001001100010011001000101110
01010100011100100110000101100011011010110010000001111001011011110111010101110
01000100000011101100110010101100011011011110111001001100100001000000110100101
11010000100000011010010111001100100000011010010110110101110000011011110111001
10111001101101001011000100110110001100101001000000111010001101111001000000110
11010110000101101001011011100111010001100001011010010110111000100000011110010
11011110111010101110010001000000110100101101110011101000110010101100111011100
10011010010111010001111001001100010011001000101110
01010100011100100110000101100011011010110010000001111001011011110111010101110
01000100000011100000110010101100011011011110111001001100100001000000110100101
11010000100000011010010111001100100000011010010110110101110000011011110111001
10111001101101001011000100110110001100101001000000111010001101111001000000110
11010110000101101001011011100111010001100001011010010110111000100000011110010
11011110111010101110010001000000110100101101110011101000110010101100111011100
10011010010111010001111001001100010011001000101110
01010100011100100110000101100011011010110010000001111001011011110111010101110
01000100000011100110110010101100011011011110111001001100100001000000110100101
11010000100000011010010111001100100000011010010110110101110000011011110111001
10111001101101001011000100110110001100101001000000111010001101111001000000110
11010110000101101001011011100111010001100001011010010110111000100000011110010
11011110111010101110010001000000110100101101110011101000110010101100111011100
10011010010111010001111001001100010011001000101110
01010100011100100110000101100011011010110010000001111001011011110111010101110
01000100000011100101110010101100011011011110111001001100100001000000110100101
11010000100000011010010111001100100000011010010110110101110000011011110111001
10111001101101001011000100110110001100101001000000111010001101111001000000110
11010110000101101001011011100111010001100001011010010110111000100000011110010
11011110111010101110010001000000110100101101110011101000110010101100111011100
10011010010111010001111001001100010011001000101110
01010100011100100110000101100011011010110010000001111001011011110111010101110
01000100000011100100010010101100011011011110111001001100100001000000110100101
11010000100000011010010111001100100000011010010110110101110000011011110111001
10111001101101001011000100110110001100101001000000111010001101111001000000110
11010110000101101001011011100111010001100001011010010110111000100000011110010
11011110111010101110010001000000110100101101110011101000110010101100111011100
10011010010111010001111001001100010011001000101110
01010100011100100110000101100011011010110010000001111001011011110111010101110
01000100000011100100100010101100011011011110111001001100100001000000110100101
11010000100000011010010111001100100000011010010110110101110000011011110111001
10111001101101001011000100110110001100101001000000111010001101111001000000110
11010110000101101001011011100111010001100001011010010110111000100000011110010
11011110111010101110010001000000110100101101110011101000110010101100111011100
10011010010111010001111001001100010011001000101110
01010100011100100110000101100011011010110010000001111001011011110111010101110
01000100000011100100111010101100011011011110111001001100100001000000110100101
11010000100000011010010111001100100000011010010110110101110000011011110111001
10111001101101001011000100110110001100101001000000111010001101111001000000110
11010110000101101001011011100111010001100001011010010110111000100000011110010
11011110111010101110010001000000110100101101110011101000110010101100111011100
10011010010111010001111001001100010011001000101110
01010100011100100110000101100011011010110010000001111001011011110111010101110
01000100000011100100110110101100011011011110111001001100100001000000110100101
11010000100000011010010111001100100000011010010110110101110000011011110111001
10111001101101001011000100110110001100101001000000111010001101111001000000110
11010110000101101001011011100111010001100001011010010110111000100000011110010

11011110111010101110010001000000110100101101110011101000110010101100111011100
10011010010111010001111001001100010011001000101110
01010100011100100110000101100011011010110010000001111001011011110111010101110
01000100000011100100110000101100011011011110111001001100100001000000110100101
11010000100000011010010111001100100000011010010110110101110000011011110111001
10111001101101001011000100110110001100101001000000111010001101111001000000110
11010110000101101001011011100111010001100001011010010110111000100000011110010
11011110111010101110010001000000110100101101110011101000110010101100111011100
10011010010111010001111001001100010011001000101110
01010100011100100110000101100011011010110010000001111001011011110111010101110
01000100000011100100110011101100011011011110111001001100100001000000110100101
11010000100000011010010111001100100000011010010110110101110000011011110111001
10111001101101001011000100110110001100101001000000111010001101111001000000110
11010110000101101001011011100111010001100001011010010110111000100000011110010
11011110111010101110010001000000110100101101110011101000110010101100111011100
10011010010111010001111001001100010011001000101110
01010100011100100110000101100011011010110010000001111001011011110111010101110
01000100000011100100110010001100011011011110111001001100100001000000110100101
11010000100000011010010111001100100000011010010110110101110000011011110111001
10111001101101001011000100110110001100101001000000111010001101111001000000110
11010110000101101001011011100111010001100001011010010110111000100000011110010
11011110111010101110010001000000110100101101110011101000110010101100111011100
10011010010111010001111001001100010011001000101110
01010100011100100110000101100011011010110010000001111001011011110111010101110
01000100000011100100110010111100011011011110111001001100100001000000110100101
11010000100000011010010111001100100000011010010110110101110000011011110111001
10111001101101001011000100110110001100101001000000111010001101111001000000110
11010110000101101001011011100111010001100001011010010110111000100000011110010
11011110111010101110010001000000110100101101110011101000110010101100111011100
10011010010111010001111001001100010011001000101110
01010100011100100110000101100011011010110010000001111001011011110111010101110
01000100000011100100110010100100011011011110111001001100100001000000110100101
11010000100000011010010111001100100000011010010110110101110000011011110111001
10111001101101001011000100110110001100101001000000111010001101111001000000110
11010110000101101001011011100111010001100001011010010110111000100000011110010
11011110111010101110010001000000110100101101110011101000110010101100111011100
10011010010111010001111001001100010011001000101110
01010100011100100110000101100011011010110010000001111001011011110111010101110
01000100000011100100110010101000011011011110111001001100100001000000110100101
11010000100000011010010111001100100000011010010110110101110000011011110111001
10111001101101001011000100110110001100101001000000111010001101111001000000110
11010110000101101001011011100111010001100001011010010110111000100000011110010
11011110111010101110010001000000110100101101110011101000110010101100111011100
10011010010111010001111001001100010011001000101110
01010100011100100110000101100011011010110010000001111001011011110111010101110
01000100000011100100110010101110011011011110111001001100100001000000110100101
11010000100000011010010111001100100000011010010110110101110000011011110111001
10111001101101001011000100110110001100101001000000111010001101111001000000110
11010110000101101001011011100111010001100001011010010110111000100000011110010
11011110111010101110010001000000110100101101110011101000110010101100111011100
10011010010111010001111001001100010011001000101110
01010100011100100110000101100011011010110010000001111001011011110111010101110
01000100000011100100110010110101011011011110111001001100100001000000110100101
11010000100000011010010111001100100000011010010110110101110000011011110111001
10111001101101001011000100110110001100101001000000111010001101111001000000110
11010110000101101001011011100111010001100001011010010110111000100000011110010

11011110111010101110010001000000110100101101110011101000110010101100111011100
10011010010111010001111001001100010011001000101110
01010100011100100110000101100011011010110010000001111001011011110111010101110
01000100000011100100110010101100111011011110111001001100100001000000110100101
11010000100000011010010111001100100000011010010110110101110000011011110111001
10111001101101001011000100110110001100101001000000111010001101111001000000110
11010110000101101001011011100111010001100001011010010110111000100000011110010
11011110111010101110010001000000110100101101110011101000110010101100111011100
10011010010111010001111001001100010011001000101110
01010100011100100110000101100011011010110010000001111001011011110111010101110
01000100000011100100110010101100001011011110111001001100100001000000110100101
11010000100000011010010111001100100000011010010110110101110000011011110111001
10111001101101001011000100110110001100101001000000111010001101111001000000110
11010110000101101001011011100111010001100001011010010110111000100000011110010
11011110111010101110010001000000110100101101110011101000110010101100111011100
10011010010111010001111001001100010011001000101110
01010100011100100110000101100011011010110010000001111001011011110111010101110
01000100000011100100110010101100010011011110111001001100100001000000110100101
11010000100000011010010111001100100000011010010110110101110000011011110111001
10111001101101001011000100110110001100101001000000111010001101111001000000110
11010110000101101001011011100111010001100001011010010110111000100000011110010
11011110111010101110010001000000110100101101110011101000110010101100111011100
10011010010111010001111001001100010011001000101110
01010100011100100110000101100011011010110010000001111001011011110111010101110
01000100000011100100110010101100011111011110111001001100100001000000110100101
11010000100000011010010111001100100000011010010110110101110000011011110111001
10111001101101001011000100110110001100101001000000111010001101111001000000110
11010110000101101001011011100111010001100001011010010110111000100000011110010
11011110111010101110010001000000110100101101110011101000110010101100111011100
10011010010111010001111001001100010011001000101110
01010100011100100110000101100011011010110010000001111001011011110111010101110
01000100000011100100110010101100011001011110111001001100100001000000110100101
11010000100000011010010111001100100000011010010110110101110000011011110111001
10111001101101001011000100110110001100101001000000111010001101111001000000110
11010110000101101001011011100111010001100001011010010110111000100000011110010
11011110111010101110010001000000110100101101110011101000110010101100111011100
10011010010111010001111001001100010011001000101110
01010100011100100110000101100011011010110010000001111001011011110111010101110
01000100000011100100110010101100011010011110111001001100100001000000110100101
11010000100000011010010111001100100000011010010110110101110000011011110111001
10111001101101001011000100110110001100101001000000111010001101111001000000110
11010110000101101001011011100111010001100001011010010110111000100000011110010
11011110111010101110010001000000110100101101110011101000110010101100111011100
10011010010111010001111001001100010011001000101110
01010100011100100110000101100011011010110010000001111001011011110111010101110
01000100000011100100110010101100011011111111011100100110010000100000001101001 01
11010000100000011010010111001100100000011010010110110101110000011011110111001
10111001101101001011000100110110001100101001000000111010001101111001000000110
11010110000101101001011011100111010001100001011010010110111000100000011110010
11011110111010101110010001000000110100101101110011101000110010101100111011100
10011010010111010001111001001100010011001000101110
01010100011100100110000101100011011010110010000001111001011011110111010101110
01000100000011100100110010101100011011001110111001001100100001000000110100101
11010000100000011010010111001100100000011010010110110101110000011011110111001
10111001101101001011000100110110001100101001000000111010001101111001000000110
11010110000101101001011011100111010001100001011010010110111000100000011110010

11011110111010101110010001000000110100101101110011101000110010101100111011100
10011010010111010001111001001100010011001000101110
01010100011100100110000101100011011010110010000001111001011011110111010101110
01000100000011100100110010101100011011010110111001001100100001000000110100101
11010000100000011010010111001100100000011010010110110101110000011011110111001
10111001101101001011000100110110001100101001000000111010001101111001000000110
11010110000101101001011011100111010001100001011010010110111000100000011110010
11011110111010101110010001000000110100101101110011101000110010101100111011100
10011010010111010001111001001100010011001000101110
01010100011100100110000101100011011010110010000001111001011011110111010101110
01000100000011100100110010101100011011011010111001001100100001000000110100101
11010000100000011010010111001100100000011010010110110101110000011011110111001
10111001101101001011000100110110001100101001000000111010001101111001000000110
11010110000101101001011011100111010001100001011010010110111000100000011110010
11011110111010101110010001000000110100101101110011101000110010101100111011100
10011010010111010001111001001100010011001000101110
01010100011100100110000101100011011010110010000001111001011011110111010101110
01000100000011100100110010101100011011011100111001001100100001000000110100101
11010000100000011010010111001100100000011010010110110101110000011011110111001
10111001101101001011000100110110001100101001000000111010001101111001000000110
11010110000101101001011011100111010001100001011010010110111000100000011110010
11011110111010101110010001000000110100101101110011101000110010101100111011100
10011010010111010001111001001100010011001000101110
01010100011100100110000101100011011010110010000001111001011011110111010101110
01000100000011100100110010101100011011011111111001001100100001000000110100101
11010000100000011010010111001100100000011010010110110101110000011011110111001
10111001101101001011000100110110001100101001000000111010001101111001000000110
11010110000101101001011011100111010001100001011010010110111000100000011110010
11011110111010101110010001000000110100101101110011101000110010101100111011100
10011010010111010001111001001100010011001000101110
01010100011100100110000101100011011010110010000001111001011011110111010101110
01000100000011100100110010101100011011011110011001001100100001000000110100101
11010000100000011010010111001100100000011010010110110101110000011011110111001
10111001101101001011000100110110001100101001000000111010001101111001000000110
11010110000101101001011011100111010001100001011010010110111000100000011110010
11011110111010101110010001000000110100101101110011101000110010101100111011100
10011010010111010001111001001100010011001000101110
01010100011100100110000101100011011010110010000001111001011011110111010101110
01000100000011100100110010101100011011011110101001001100100001000000110100101
11010000100000011010010111001100100000011010010110110101110000011011110111001
10111001101101001011000100110110001100101001000000111010001101111001000000110
11010110000101101001011011100111010001100001011010010110111000100000011110010
11011110111010101110010001000000110100101101110011101000110010101100111011100
10011010010111010001111001001100010011001000101110
01010100011100100110000101100011011010110010000001111001011011110111010101110
01000100000011100100110010101100011011011110110001001100100001000000110100101
11010000100000011010010111001100100000011010010110110101110000011011110111001
10111001101101001011000100110110001100101001000000111010001101111001000000110
11010110000101101001011011100111010001100001011010010110111000100000011110010
11011110111010101110010001000000110100101101110011101000110010101100111011100
10011010010111010001111001001100010011001000101110
01010100011100100110000101100011011010110010000001111001011011110111010101110
01000100000011100100110010101100011011011110111101001100100001000000110100101
11010000100000011010010111001100100000011010010110110101110000011011110111001
10111001101101001011000100110110001100101001000000111010001101111001000000110
11010110000101101001011011100111010001100001011010010110111000100000011110010

11011110111010101110010001000000110100101101110011101000110010101100111011100
10011010010111010001111001001100010011001000101110
01010100011100100110000101100011011010110010000001111001011011110111010101110
01000100000011100100110010101100011011011110111011001100100001000000110100101
11010000100000011010010111001100100000011010010110110101110000011011110111001
10111001101101001011000100110110001100101001000000111010001101111001000000110
11010110000101101001011011100111010001100001011010010110111000100000011110010
11011110111010101110010001000000110100101101110011101000110010101100111011100
10011010010111010001111001001100010011001000101110
01010100011100100110000101100011011010110010000001111001011011110111010101110
01000100000011100100110010101100011011011110111000001100100001000000110100101
11010000100000011010010111001100100000011010010110110101110000011011110111001
10111001101101001011000100110110001100101001000000111010001101111001000000110
11010110000101101001011011100111010001100001011010010110111000100000011110010
11011110111010101110010001000000110100101101110011101000110010101100111011100
10011010010111010001111001001100010011001000101110
01010100011100100110000101100011011010110010000001111001011011110111010101110
01000100000011100100110010101100011011011110111001101100100001000000110100101
11010000100000011010010111001100100000011010010110110101110000011011110111001
10111001101101001011000100110110001100101001000000111010001101111001000000110
11010110000101101001011011100111010001100001011010010110111000100000011110010
11011110111010101110010001000000110100101101110011101000110010101100111011100
10011010010111010001111001001100010011001000101110

File 3: “Cats always innocent because dogs hate them without ABC reasons.”

01000011011000010111010001110011001000000110000101101100011101110110000101111
00101110011001000000110100101101110011011100110111101100011011001010110111001
11010000100000011000100110010101100011011000010111010101110011011001010010000
00110010001101111011001110111001100100000011010000110000101110100011001010010
00000111010001101000011001010110110100100000011101110110100101110100011010000
11011110111010101110100001000000100000101000010010000110010000001110010011001
01011000010111001101101111011011100111001100101110
00000011011000010111010001110011001000000110000101101100011101110110000101111
00101110011001000000110100101101110011011100110111101100011011001010110111001
11010000100000011000100110010101100011011000010111010101110011011001010010000
00110010001101111011001110111001100100000011010000110000101110100011001010010
00000111010001101000011001010110110100100000011101110110100101110100011010000
11011110111010101110100001000000100000101000010010000110010000001110010011001
01011000010111001101101111011011100111001100101110
01100011011000010111010001110011001000000110000101101100011101110110000101111
00101110011001000000110100101101110011011100110111101100011011001010110111001
11010000100000011000100110010101100011011000010111010101110011011001010010000
00110010001101111011001110111001100100000011010000110000101110100011001010010
00000111010001101000011001010110110100100000011101110110100101110100011010000
11011110111010101110100001000000100000101000010010000110010000001110010011001
01011000010111001101101111011011100111001100101110
01010011011000010111010001110011001000000110000101101100011101110110000101111
00101110011001000000110100101101110011011100110111101100011011001010110111001
11010000100000011000100110010101100011011000010111010101110011011001010010000
00110010001101111011001110111001100100000011010000110000101110100011001010010
00000111010001101000011001010110110100100000011101110110100101110100011010000
11011110111010101110100001000000100000101000010010000110010000001110010011001
01011000010111001101101111011011100111001100101110
01001011011000010111010001110011001000000110000101101100011101110110000101111
00101110011001000000110100101101110011011100110111101100011011001010110111001

11010000100000011000100110010101100011011000010111010101110011011001010010000
00110010001101111011001110111001100100000011010000110000101110100011001010010
00000111010001101000011001010110110100100000011101110110100101110100011010000
1101111011101010111010000100000010000001010000100100001100100000001110010011001
01011000010111001101101111011011100111001100101110
01000111011000010111010001110011001000000110000101101100011101110110000101111
00101110011001000000110100101101110011011100110111101100011011001010110111001
11010000100000011000100110010101100011011000010111010101110011011001010010000
00110010001101111011001110111001100100000011010000110000101110100011001010010
00000111010001101000011001010110110100100000011101110110100101110100011010000
1101111011101010111010000100000010000001010000100100001100100000001110010011001
01011000010111001101101111011011100111001100101110
01000001011000010111010001110011001000000110000101101100011101110110000101111
00101110011001000000110100101101110011011100110111101100011011001010110111001
11010000100000011000100110010101100011011000010111010101110011011001010010000
00110010001101111011001110111001100100000011010000110000101110100011001010010
00000111010001101000011001010110110100100000011101110110100101110100011010000
1101111011101010111010000100000010000001010000100100001100100000001110010011001
01011000010111001101101111011011100111001100101110
01000010011000010111010001110011001000000110000101101100011101110110000101111
00101110011001000000110100101101110011011100110111101100011011001010110111001
11010000100000011000100110010101100011011000010111010101110011011001010010000
00110010001101111011001110111001100100000011010000110000101110100011001010010
00000111010001101000011001010110110100100000011101110110100101110100011010000
1101111011101010111010000100000010000001010000100100001100100000001110010011001
01011000010111001101101111011011100111001100101110
01000011111000010111010001110011001000000110000101101100011101110110000101111
00101110011001000000110100101101110011011100110111101100011011001010110111001
11010000100000011000100110010101100011011000010111010101110011011001010010000
00110010001101111011001110111001100100000011010000110000101110100011001010010
00000111010001101000011001010110110100100000011101110110100101110100011010000
1101111011101010111010000100000010000001010000100100001100100000001110010011001
01011000010111001101101111011011100111001100101110
01000011001000010111010001110011001000000110000101101100011101110110000101111
00101110011001000000110100101101110011011100110111101100011011001010110111001
11010000100000011000100110010101100011011000010111010101110011011001010010000
00110010001101111011001110111001100100000011010000110000101110100011001010010
00000111010001101000011001010110110100100000011101110110100101110100011010000
1101111011101010111010000100000010000001010000100100001100100000001110010011001
01011000010111001101101111011011100111001100101110
01000011010000010111010001110011001000000110000101101100011101110110000101111
00101110011001000000110100101101110011011100110111101100011011001010110111001
11010000100000011000100110010101100011011000010111010101110011011001010010000
00110010001101111011001110111001100100000011010000110000101110100011001010010
00000111010001101000011001010110110100100000011101110110100101110100011010000
1101111011101010111010000100000010000001010000100100001100100000001110010011001
01011000010111001101101111011011100111001100101110
01000011011100010111010001110011001000000110000101101100011101110110000101111
00101110011001000000110100101101110011011100110111101100011011001010110111001
11010000100000011000100110010101100011011000010111010101110011011001010010000
00110010001101111011001110111001100100000011010000110000101110100011001010010
00000111010001101000011001010110110100100000011101110110100101110100011010000
1101111011101010111010000100000010000001010000100100001100100000001110010011001
01011000010111001101101111011011100111001100101110
01000011011010010111010001110011001000000110000101101100011101110110000101111
00101110011001000000110100101101110011011100110111101100011011001010110111001
11010000100000011000100110010101100011011000010111010101110011011001010010000

00110010001101111011001110111001100100000011010000110000101110100011001010010
00000111010001101000011001010110110100100000011101110110100101110100011010000
11011110111010101110100001000000100000101000010010000110010000001110010011001
01011000010111001101101111011011100111001100101110
01000011011001010111010001110011001000000110000101101100011101110110000101111
00101110011001000000110100101101110011011100110111101100011011001010110111001
11010000100000011000100110010101100011011000010111010101110011011001010010000
00110010001101111011001110111001100100000011010000110000101110100011001010010
00000111010001101000011001010110110100100000011101110110100101110100011010000
11011110111010101110100001000000100000101000010010000110010000001110010011001
01011000010111001101101111011011100111001100101110
01000011011000110111010001110011001000000110000101101100011101110110000101111
00101110011001000000110100101101110011011100110111101100011011001010110111001
11010000100000011000100110010101100011011000010111010101110011011001010010000
00110010001101111011001110111001100100000011010000110000101110100011001010010
00000111010001101000011001010110110100100000011101110110100101110100011010000
11011110111010101110100001000000100000101000010010000110010000001110010011001
01011000010111001101101111011011100111001100101110
01000011011000000111010001110011001000000110000101101100011101110110000101111
00101110011001000000110100101101110011011100110111101100011011001010110111001
11010000100000011000100110010101100011011000010111010101110011011001010010000
00110010001101111011001110111001100100000011010000110000101110100011001010010
00000111010001101000011001010110110100100000011101110110100101110100011010000
11011110111010101110100001000000100000101000010010000110010000001110010011001
01011000010111001101101111011011100111001100101110
01000011011000011111010001110011001000000110000101101100011101110110000101111
00101110011001000000110100101101110011011100110111101100011011001010110111001
11010000100000011000100110010101100011011000010111010101110011011001010010000
00110010001101111011001110111001100100000011010000110000101110100011001010010
00000111010001101000011001010110110100100000011101110110100101110100011010000
11011110111010101110100001000000100000101000010010000110010000001110010011001
01011000010111001101101111011011100111001100101110
01000011011000010011010001110011001000000110000101101100011101110110000101111
00101110011001000000110100101101110011011100110111101100011011001010110111001
11010000100000011000100110010101100011011000010111010101110011011001010010000
00110010001101111011001110111001100100000011010000110000101110100011001010010
00000111010001101000011001010110110100100000011101110110100101110100011010000
11011110111010101110100001000000100000101000010010000110010000001110010011001
01011000010111001101101111011011100111001100101110
01000011011000010101010001110011001000000110000101101100011101110110000101111
00101110011001000000110100101101110011011100110111101100011011001010110111001
11010000100000011000100110010101100011011000010111010101110011011001010010000
00110010001101111011001110111001100100000011010000110000101110100011001010010
00000111010001101000011001010110110100100000011101110110100101110100011010000
11011110111010101110100001000000100000101000010010000110010000001110010011001
01011000010111001101101111011011100111001100101110
01000011011000010110010001110011001000000110000101101100011101110110000101111
00101110011001000000110100101101110011011100110111101100011011001010110111001
11010000100000011000100110010101100011011000010111010101110011011001010010000
00110010001101111011001110111001100100000011010000110000101110100011001010010
00000111010001101000011001010110110100100000011101110110100101110100011010000
11011110111010101110100001000000100000101000010010000110010000001110010011001
01011000010111001101101111011011100111001100101110
01000011011000010111110001110011001000000110000101101100011101110110000101111
00101110011001000000110100101101110011011100110111101100011011001010110111001
11010000100000011000100110010101100011011000010111010101110011011001010010000
00110010001101111011001110111001100100000011010000110000101110100011001010010

00000111010001101000011001010110110100100000011101110110100101110100011010000
11011110111010101110100001000000100000101000010010000110010000001110010011001
01011000010111001101101111011011100111001100101110
01000011011000010111000001110011001000000110000101101100011101110110000101111
00101110011001000000110100101101110011011100110111101100011011001010110111001
11010000100000011000100110010101100011011000010111010101110011011001010010000
00110010001101111011001110111001100100000011010000110000101110100011001010010
00000111010001101000011001010110110100100000011101110110100101110100011010000
11011110111010101110100001000000100000101000010010000110010000001110010011001
01011000010111001101101111011011100111001100101110
01000011011000010111011001110011001000000110000101101100011101110110000101111
00101110011001000000110100101101110011011100110111101100011011001010110111001
11010000100000011000100110010101100011011000010111010101110011011001010010000
00110010001101111011001110111001100100000011010000110000101110100011001010010
00000111010001101000011001010110110100100000011101110110100101110100011010000
11011110111010101110100001000000100000101000010010000110010000001110010011001
01011000010111001101101111011011100111001100101110
01000011011000010111010101110011001000000110000101101100011101110110000101111
00101110011001000000110100101101110011011100110111101100011011001010110111001
11010000100000011000100110010101100011011000010111010101110011011001010010000
00110010001101111011001110111001100100000011010000110000101110100011001010010
00000111010001101000011001010110110100100000011101110110100101110100011010000
11011110111010101110100001000000100000101000010010000110010000001110010011001
01011000010111001101101111011011100111001100101110
01000011011000010111010011110011001000000110000101101100011101110110000101111
00101110011001000000110100101101110011011100110111101100011011001010110111001
11010000100000011000100110010101100011011000010111010101110011011001010010000
00110010001101111011001110111001100100000011010000110000101110100011001010010
00000111010001101000011001010110110100100000011101110110100101110100011010000
11011110111010101110100001000000100000101000010010000110010000001110010011001
01011000010111001101101111011011100111001100101110
01000011011000010111010000110011001000000110000101101100011101110110000101111
00101110011001000000110100101101110011011100110111101100011011001010110111001
11010000100000011000100110010101100011011000010111010101110011011001010010000
00110010001101111011001110111001100100000011010000110000101110100011001010010
00000111010001101000011001010110110100100000011101110110100101110100011010000
11011110111010101110100001000000100000101000010010000110010000001110010011001
01011000010111001101101111011011100111001100101110
01000011011000010111010001010011001000000110000101101100011101110110000101111
00101110011001000000110100101101110011011100110111101100011011001010110111001
11010000100000011000100110010101100011011000010111010101110011011001010010000
00110010001101111011001110111001100100000011010000110000101110100011001010010
00000111010001101000011001010110110100100000011101110110100101110100011010000
11011110111010101110100001000000100000101000010010000110010000001110010011001
01011000010111001101101111011011100111001100101110
01000011011000010111010001100011001000000110000101101100011101110110000101111
00101110011001000000110100101101110011011100110111101100011011001010110111001
11010000100000011000100110010101100011011000010111010101110011011001010010000
00110010001101111011001110111001100100000011010000110000101110100011001010010
00000111010001101000011001010110110100100000011101110110100101110100011010000
11011110111010101110100001000000100000101000010010000110010000001110010011001
01011000010111001101101111011011100111001100101110
01000011011000010111010001111011001000000110000101101100011101110110000101111
00101110011001000000110100101101110011011100110111101100011011001010110111001
11010000100000011000100110010101100011011000010111010101110011011001010010000
00110010001101111011001110111001100100000011010000110000101110100011001010010
00000111010001101000011001010110110100100000011101110110100101110100011010000

11011110111010101110100001000000010000001010000100100001100100000001110010011001
01011000010111001101101111011011100111001100101110
01000011011000010111010001110111001000000000110000101101100011101110110000101111
00101110011001000000000110100101101110011011100110111101100011011001010110111001
11010000100000000011000100110010101100011011000010111010101110011011001010010000
00110010001101111011001110111001100100000000011010000110000101110100011001010010
00000111010001101000011001010110110100100000000011101110110100101110100011010000
11011110111010101110100001000000010000001010000100100001100100000001110010011001
01011000010111001101101111011011100111001100101110
01000011011000010111010001110001001000000000110000101101100011101110110000101111
00101110011001000000000110100101101110011011100110111101100011011001010110111001
11010000100000000011000100110010101100011011000010111010101110011011001010010000
00110010001101111011001110111001100100000000011010000110000101110100011001010010
00000111010001101000011001010110110100100000000011101110110100101110100011010000
11011110111010101110100001000000010000001010000100100001100100000001110010011001
01011000010111001101101111011011100111001100101110
01000011011000010111010001110010001000000000110000101101100011101110110000101111
00101110011001000000000110100101101110011011100110111101100011011001010110111001
11010000100000000011000100110010101100011011000010111010101110011011001010010000
00110010001101111011001110111001100100000000011010000110000101110100011001010010
00000111010001101000011001010110110100100000000011101110110100101110100011010000
11011110111010101110100001000000010000001010000100100001100100000001110010011001
01011000010111001101101111011011100111001100101110
01000011011000010111010001110011101000000000110000101101100011101110110000101111
00101110011001000000000110100101101110011011100110111101100011011001010110111001
11010000100000000011000100110010101100011011000010111010101110011011001010010000
00110010001101111011001110111001100100000000011010000110000101110100011001010010
00000111010001101000011001010110110100100000000011101110110100101110100011010000
11011110111010101110100001000000010000001010000100100001100100000001110010011001
01011000010111001101101111011011100111001100101110
01000011011000010111010001110011011000000000110000101101100011101110110000101111
00101110011001000000000110100101101110011011100110111101100011011001010110111001
11010000100000000011000100110010101100011011000010111010101110011011001010010000
00110010001101111011001110111001100100000000011010000110000101110100011001010010
00000111010001101000011001010110110100100000000011101110110100101110100011010000
11011110111010101110100001000000010000001010000100100001100100000001110010011001
01011000010111001101101111011011100111001100101110
01000011011000010111010001110011000000000000110000101101100011101110110000101111
00101110011001000000000110100101101110011011100110111101100011011001010110111001
11010000100000000011000100110010101100011011000010111010101110011011001010010000
00110010001101111011001110111001100100000000011010000110000101110100011001010010
00000111010001101000011001010110110100100000000011101110110100101110100011010000
11011110111010101110100001000000010000001010000100100001100100000001110010011001
01011000010111001101101111011011100111001100101110
01000011011000010111010001110011001100000000110000101101100011101110110000101111
00101110011001000000000110100101101110011011100110111101100011011001010110111001
11010000100000000011000100110010101100011011000010111010101110011011001010010000
00110010001101111011001110111001100100000000011010000110000101110100011001010010
00000111010001101000011001010110110100100000000011101110110100101110100011010000
11011110111010101110100001000000010000001010000100100001100100000001110010011001
01011000010111001101101111011011100111001100101110
01000011011000010111010001110011001010000000110000101101100011101110110000101111
00101110011001000000000110100101101110011011100110111101100011011001010110111001
11010000100000000011000100110010101100011011000010111010101110011011001010010000
00110010001101111011001110111001100100000000011010000110000101110100011001010010
00000111010001101000011001010110110100100000000011101110110100101110100011010000

11011110111010101110100001000000100000101000010010000110010000001110010011001
01011000010111001101101111011011100111001100101110
01000011011000010111010001110011001001000110000101101100011101110110000101111
00101110011001000000110100101101110011011100110111101100011011001010110111001
11010000100000011000100110010101100011011000010111010101110011011001010010000
00110010001101111011001110111001100100000011010000110000101110100011001010010
00000111010001101000011001010110110100100000011101110110100101110100011010000
11011110111010101110100001000000100000101000010010000110010000001110010011001
01011000010111001101101111011011100111001100101110
01000011011000010111010001110011001000100110000101101100011101110110000101111
00101110011001000000110100101101110011011100110111101100011011001010110111001
11010000100000011000100110010101100011011000010111010101110011011001010010000
00110010001101111011001110111001100100000011010000110000101110100011001010010
00000111010001101000011001010110110100100000011101110110100101110100011010000
11011110111010101110100001000000100000101000010010000110010000001110010011001
01011000010111001101101111011011100111001100101110
01000011011000010111010001110011001000010110000101101100011101110110000101111
00101110011001000000110100101101110011011100110111101100011011001010110111001
11010000100000011000100110010101100011011000010111010101110011011001010010000
00110010001101111011001110111001100100000011010000110000101110100011001010010
00000111010001101000011001010110110100100000011101110110100101110100011010000
11011110111010101110100001000000100000101000010010000110010000001110010011001
01011000010111001101101111011011100111001100101110
01000011011000010111010001110011001000001110000101101100011101110110000101111
00101110011001000000110100101101110011011100110111101100011011001010110111001
11010000100000011000100110010101100011011000010111010101110011011001010010000
00110010001101111011001110111001100100000011010000110000101110100011001010010
00000111010001101000011001010110110100100000011101110110100101110100011010000
11011110111010101110100001000000100000101000010010000110010000001110010011001
01011000010111001101101111011011100111001100101110
01000011011000010111010001110011001000000100000101101100011101110110000101111
00101110011001000000110100101101110011011100110111101100011011001010110111001
11010000100000011000100110010101100011011000010111010101110011011001010010000
00110010001101111011001110111001100100000011010000110000101110100011001010010
00000111010001101000011001010110110100100000011101110110100101110100011010000
11011110111010101110100001000000100000101000010010000110010000001110010011001
01011000010111001101101111011011100111001100101110
01000011011000010111010001110011001000000100000101101100011101110110000101111
00101110011001000000110100101101110011011100110111101100011011001010110111001
11010000100000011000100110010101100011011000010111010101110011011001010010000
00110010001101111011001110111001100100000011010000110000101110100011001010010
00000111010001101000011001010110110100100000011101110110100101110100011010000
11011110111010101110100001000000100000101000010010000110010000001110010011001
01011000010111001101101111011011100111001100101110
01000011011000010111010001110011001000000111000101101100011101110110000101111
00101110011001000000110100101101110011011100110111101100011011001010110111001
11010000100000011000100110010101100011011000010111010101110011011001010010000
00110010001101111011001110111001100100000011010000110000101110100011001010010
00000111010001101000011001010110110100100000011101110110100101110100011010000
11011110111010101110100001000000100000101000010010000110010000001110010011001
01011000010111001101101111011011100111001100101110
01000011011000010111010001110011001000000110100101101100011101110110000101111
00101110011001000000110100101101110011011100110111101100011011001010110111001
11010000100000011000100110010101100011011000010111010101110011011001010010000
00110010001101111011001110111001100100000011010000110000101110100011001010010
00000111010001101000011001010110110100100000011101110110100101110100011010000

11011110111010101110100001000000100000101000010010000110010000001110010011001
01011000010111001101101111011011100111001100101110
01000011011000010111010001110011001000000110010101101100011101110110000101111
00101110011001000000110100101101110011011100110111101100011011001010110111001
11010000100000011000100110010101100011011000010111010101110011011001010010000
00110010001101111011001110111001100100000011010000110000101110100011001010010
00000111010001101000011001010110110100100000011101110110100101110100011010000
11011110111010101110100001000000100000101000010010000110010000001110010011001
01011000010111001101101111011011100111001100101110
01000011011000010111010001110011001000000110001101101100011101110110000101111
00101110011001000000110100101101110011011100110111101100011011001010110111001
11010000100000011000100110010101100011011000010111010101110011011001010010000
00110010001101111011001110111001100100000011010000110000101110100011001010010
00000111010001101000011001010110110100100000011101110110100101110100011010000
11011110111010101110100001000000100000101000010010000110010000001110010011001
01011000010111001101101111011011100111001100101110
01000011011000010111010001110011001000000110000001101100011101110110000101111
00101110011001000000110100101101110011011100110111101100011011001010110111001
11010000100000011000100110010101100011011000010111010101110011011001010010000
00110010001101111011001110111001100100000011010000110000101110100011001010010
00000111010001101000011001010110110100100000011101110110100101110100011010000
11011110111010101110100001000000100000101000010010000110010000001110010011001
01011000010111001101101111011011100111001100101110
01000011011000010111010001110011001000000110000111101100011101110110000101111
00101110011001000000110100101101110011011100110111101100011011001010110111001
11010000100000011000100110010101100011011000010111010101110011011001010010000
00110010001101111011001110111001100100000011010000110000101110100011001010010
00000111010001101000011001010110110100100000011101110110100101110100011010000
11011110111010101110100001000000100000101000010010000110010000001110010011001
01011000010111001101101111011011100111001100101110
01000011011000010111010001110011001000000110000100101100011101110110000101111
00101110011001000000110100101101110011011100110111101100011011001010110111001
11010000100000011000100110010101100011011000010111010101110011011001010010000
00110010001101111011001110111001100100000011010000110000101110100011001010010
00000111010001101000011001010110110100100000011101110110100101110100011010000
11011110111010101110100001000000100000101000010010000110010000001110010011001
01011000010111001101101111011011100111001100101110
01000011011000010111010001110011001000000110000101001100011101110110000101111
00101110011001000000110100101101110011011100110111101100011011001010110111001
11010000100000011000100110010101100011011000010111010101110011011001010010000
00110010001101111011001110111001100100000011010000110000101110100011001010010
00000111010001101000011001010110110100100000011101110110100101110100011010000
11011110111010101110100001000000100000101000010010000110010000001110010011001
01011000010111001101101111011011100111001100101110
01000011011000010111010001110011001000000110000101111100011101110110000101111
00101110011001000000110100101101110011011100110111101100011011001010110111001
11010000100000011000100110010101100011011000010111010101110011011001010010000
00110010001101111011001110111001100100000011010000110000101110100011001010010
00000111010001101000011001010110110100100000011101110110100101110100011010000
11011110111010101110100001000000100000101000010010000110010000001110010011001
01011000010111001101101111011011100111001100101110
01000011011000010111010001110011001000000110000101100100011101110110000101111
00101110011001000000110100101101110011011100110111101100011011001010110111001
11010000100000011000100110010101100011011000010111010101110011011001010010000
00110010001101111011001110111001100100000011010000110000101110100011001010010
00000111010001101000011001010110110100100000011101110110100101110100011010000

11011110111010101110100001000000100000101000010010000110010000001110010011001
01011000010111001101101111011011100111001100101110
01000011011000010111010001110011001000000110000101101000011101110110000101111
00101110011001000000110100101101110011011100110111101100011011001010110111001
11010000100000011000100110010101100011011000010111010101110011011001010010000
00110010001101111011001110111001100100000011010000110000101110100011001010010
00000111010001101000011001010110110100100000011101110110100101110100011010000
11011110111010101110100001000000100000101000010010000110010000001110010011001
01011000010111001101101111011011100111001100101110
01000011011000010111010001110011001000000110000101101110011101110110000101111
00101110011001000000110100101101110011011100110111101100011011001010110111001
11010000100000011000100110010101100011011000010111010101110011011001010010000
00110010001101111011001110111001100100000011010000110000101110100011001010010
00000111010001101000011001010110110100100000011101110110100101110100011010000
11011110111010101110100001000000100000101000010010000110010000001110010011001
01011000010111001101101111011011100111001100101110
01000011011000010111010001110011001000000110000101101101011101110110000101111
00101110011001000000110100101101110011011100110111101100011011001010110111001
11010000100000011000100110010101100011011000010111010101110011011001010010000
00110010001101111011001110111001100100000011010000110000101110100011001010010
00000111010001101000011001010110110100100000011101110110100101110100011010000
11011110111010101110100001000000100000101000010010000110010000001110010011001
01011000010111001101101111011011100111001100101110
01000011011000010111010001110011001000000110000101101100111101110110000101111
00101110011001000000110100101101110011011100110111101100011011001010110111001
11010000100000011000100110010101100011011000010111010101110011011001010010000
00110010001101111011001110111001100100000011010000110000101110100011001010010
00000111010001101000011001010110110100100000011101110110100101110100011010000
11011110111010101110100001000000100000101000010010000110010000001110010011001
01011000010111001101101111011011100111001100101110
01000011011000010111010001110011001000000110000101101100001101110110000101111
00101110011001000000110100101101110011011100110111101100011011001010110111001
11010000100000011000100110010101100011011000010111010101110011011001010010000
00110010001101111011001110111001100100000011010000110000101110100011001010010
00000111010001101000011001010110110100100000011101110110100101110100011010000
11011110111010101110100001000000100000101000010010000110010000001110010011001
01011000010111001101101111011011100111001100101110
01000011011000010111010001110011001000000110000101101100010101110110000101111
00101110011001000000110100101101110011011100110111101100011011001010110111001
11010000100000011000100110010101100011011000010111010101110011011001010010000
00110010001101111011001110111001100100000011010000110000101110100011001010010
00000111010001101000011001010110110100100000011101110110100101110100011010000
11011110111010101110100001000000100000101000010010000110010000001110010011001
01011000010111001101101111011011100111001100101110
01000011011000010111010001110011001000000110000101101100011001110110000101111
00101110011001000000110100101101110011011100110111101100011011001010110111001
11010000100000011000100110010101100011011000010111010101110011011001010010000
00110010001101111011001110111001100100000011010000110000101110100011001010010
00000111010001101000011001010110110100100000011101110110100101110100011010000
11011110111010101110100001000000100000101000010010000110010000001110010011001
01011000010111001101101111011011100111001100101110
01000011011000010111010001110011001000000110000101101100011111110110000101111
00101110011001000000110100101101110011011100110111101100011011001010110111001
11010000100000011000100110010101100011011000010111010101110011011001010010000
00110010001101111011001110111001100100000011010000110000101110100011001010010
00000111010001101000011001010110110100100000011101110110100101110100011010000

1101111011101010111010000100000001000000101000010010000110010000001110010011001
01011000010111001101101111011011100111001100101110
01000011011000010111010001110011001000000110000101101100011100110110000101111
001011100110010000000110100101101110011011100110111101100011011001010110111001
110100001000000011000100110010101100011011000010111010101110011011001010010000
001100100011011110110011101110011001000000011010000110000101110100011001010010
000001110100011010000110010101101101001000000011101110110100101110100011010000
1101111011101010111010000100000001000000101000010010000110010000001110010011001
01011000010111001101101111011011100111001100101110
01000011011000010111010001110011001000000110000101101100011101010110000101111
001011100110010000000110100101101110011011100110111101100011011001010110111001
110100001000000011000100110010101100011011000010111010101110011011001010010000
001100100011011110110011101110011001000000011010000110000101110100011001010010
000001110100011010000110010101101101001000000011101110110100101110100011010000
1101111011101010111010000100000001000000101000010010000110010000001110010011001
01011000010111001101101111011011100111001100101110
01000011011000010111010001110011001000000110000101101100011101100110000101111
001011100110010000000110100101101110011011100110111101100011011001010110111001
110100001000000011000100110010101100011011000010111010101110011011001010010000
001100100011011110110011101110011001000000011010000110000101110100011001010010
000001110100011010000110010101101101001000000011101110110100101110100011010000
1101111011101010111010000100000001000000101000010010000110010000001110010011001
01011000010111001101101111011011100111001100101110
01000011011000010111010001110011001000000110000101101100011101111110000101111
001011100110010000000110100101101110011011100110111101100011011001010110111001
110100001000000011000100110010101100011011000010111010101110011011001010010000
001100100011011110110011101110011001000000011010000110000101110100011001010010
000001110100011010000110010101101101001000000011101110110100101110100011010000
1101111011101010111010000100000001000000101000010010000110010000001110010011001
01011000010111001101101111011011100111001100101110
01000011011000010111010001110011001000000110000101101100011101110010000101111
001011100110010000000110100101101110011011100110111101100011011001010110111001
110100001000000011000100110010101100011011000010111010101110011011001010010000
001100100011011110110011101110011001000000011010000110000101110100011001010010
000001110100011010000110010101101101001000000011101110110100101110100011010000
1101111011101010111010000100000001000000101000010010000110010000001110010011001
01011000010111001101101111011011100111001100101110
01000011011000010111010001110011001000000110000101101100011101110100000101111
001011100110010000000110100101101110011011100110111101100011011001010110111001
110100001000000011000100110010101100011011000010111010101110011011001010010000
001100100011011110110011101110011001000000011010000110000101110100011001010010
000001110100011010000110010101101101001000000011101110110100101110100011010000
1101111011101010111010000100000001000000101000010010000110010000001110010011001
01011000010111001101101111011011100111001100101110
01000011011000010111010001110011001000000110000101101100011101110111000101111
001011100110010000000110100101101110011011100110111101100011011001010110111001
110100001000000011000100110010101100011011000010111010101110011011001010010000
001100100011011110110011101110011001000000011010000110000101110100011001010010
000001110100011010000110010101101101001000000011101110110100101110100011010000
1101111011101010111010000100000001000000101000010010000110010000001110010011001
01011000010111001101101111011011100111001100101110
01000011011000010111010001110011001000000110000101101100011101110110100101111
001011100110010000000110100101101110011011100110111101100011011001010110111001
110100001000000011000100110010101100011011000010111010101110011011001010010000
001100100011011110110011101110011001000000011010000110000101110100011001010010
000001110100011010000110010101101101001000000011101110110100101110100011010000

110111101110101011101000010000000100000101000010010000110010000001110010011001
01011000010111001101101111011011100111001100101110
010000110110000101110100011100110010000000110000101101100011101110110010101111
001011100110010000000110100101101110011011100110111101100011011001010110111001
110100001000000110001001100101011000110110000101110101011100110110010100100000
001100100011011110110011101110011001000000110100001100001011101000110010100 10
000001110100011010000110010101101101001000000111011101101001011101000110100 00
110111101110101011101000010000000100000101000010010000110010000001110010011001
01011000010111001101101111011011100111001100101110
010000110110000101110100011100110010000000110000101101100011101110110001101111
001011100110010000000110100101101110011011100110111101100011011001010110111001
110100001000000110001001100101011000110110000101110101011100110110010100100000
001100100011011110110011101110011001000000110100001100001011101000110010100 10
000001110100011010000110010101101101001000000111011101101001011101000110100 00
110111101110101011101000010000000100000101000010010000110010000001110010011001
01011000010111001101101111011011100111001100101110
010000110110000101110100011100110010000000110000101101100011101110110000001111
001011100110010000000110100101101110011011100110111101100011011001010110111001
110100001000000110001001100101011000110110000101110101011100110110010100100000
001100100011011110110011101110011001000000110100001100001011101000110010100 10
000001110100011010000110010101101101001000000111011101101001011101000110100 00
110111101110101011101000010000000100000101000010010000110010000001110010011001
01011000010111001101101111011011100111001100101110
010000110110000101110100011100110010000000110000101101100011101110110000111111
001011100110010000000110100101101110011011100110111101100011011001010110111001
110100001000000110001001100101011000110110000101110101011100110110010100100000
001100100011011110110011101110011001000000110100001100001011101000110010100 10
000001110100011010000110010101101101001000000111011101101001011101000110100 00
110111101110101011101000010000000100000101000010010000110010000001110010011001
01011000010111001101101111011011100111001100101110
010000110110000101110100011100110010000000110000101101100011101110110000100111
001011100110010000000110100101101110011011100110111101100011011001010110111001
110100001000000110001001100101011000110110000101110101011100110110010100100000
001100100011011110110011101110011001000000110100001100001011101000110010100 10
000001110100011010000110010101101101001000000111011101101001011101000110100 00
110111101110101011101000010000000100000101000010010000110010000001110010011001
01011000010111001101101111011011100111001100101110
010000110110000101110100011100110010000000110000101101100011101110110000101011
001011100110010000000110100101101110011011100110111101100011011001010110111001
110100001000000110001001100101011000110110000101110101011100110110010100100000
001100100011011110110011101110011001000000110100001100001011101000110010100 10
000001110100011010000110010101101101001000000111011101101001011101000110100 00
110111101110101011101000010000000100000101000010010000110010000001110010011001
01011000010111001101101111011011100111001100101110
010000110110000101110100011100110010000000110000101101100011101110110000101101
001011100110010000000110100101101110011011100110111101100011011001010110111001
110100001000000110001001100101011000110110000101110101011100110110010100100000
001100100011011110110011101110011001000000110100001100001011101000110010100 10
000001110100011010000110010101101101001000000111011101101001011101000110100 00
110111101110101011101000010000000100000101000010010000110010000001110010011001
01011000010111001101101111011011100111001100101110
010000110110000101110100011100110010000000110000101101100011101110110000101110
001011100110010000000110100101101110011011100110111101100011011001010110111001
110100001000000110001001100101011000110110000101110101011100110110010100100000
001100100011011110110011101110011001000000110100001100001011101000110010100 10
000001110100011010000110010101101101001000000111011101101001011101000110100 00

11011110111010101110100001000000100000101000010010000110010000001110010011001
01011000010111001101101111011011100111001100101110
01000011011000010111010001110011001000000110000101101100011101110110000101111
10101110011001000000110100101101110011011100110111101100011011001010110111001
11010000100000011000100110010101100011011000010111010101110011011001010010000
00110010001101111011001110111001100100000011010000110000101110100011001010010
00000111010001101000011001010110110100100000011101110110100101110100011010000
11011110111010101110100001000000100000101000010010000110010000001110010011001
01011000010111001101101111011011100111001100101110
01000011011000010111010001110011001000000110000101101100011101110110000101111
01101110011001000000110100101101110011011100110111101100011011001010110111001
11010000100000011000100110010101100011011000010111010101110011011001010010000
00110010001101111011001110111001100100000011010000110000101110100011001010010
00000111010001101000011001010110110100100000011101110110100101110100011010000
11011110111010101110100001000000100000101000010010000110010000001110010011001
01011000010111001101101111011011100111001100101110
01000011011000010111010001110011001000000110000101101100011101110110000101111
00001110011001000000110100101101110011011100110111101100011011001010110111001
11010000100000011000100110010101100011011000010111010101110011011001010010000
00110010001101111011001110111001100100000011010000110000101110100011001010010
00000111010001101000011001010110110100100000011101110110100101110100011010000
11011110111010101110100001000000100000101000010010000110010000001110010011001
01011000010111001101101111011011100111001100101110
01000011011000010111010001110011001000000110000101101100011101110110000101111
00111110011001000000110100101101110011011100110111101100011011001010110111001
11010000100000011000100110010101100011011000010111010101110011011001010010000
00110010001101111011001110111001100100000011010000110000101110100011001010010
00000111010001101000011001010110110100100000011101110110100101110100011010000
11011110111010101110100001000000100000101000010010000110010000001110010011001
01011000010111001101101111011011100111001100101110
01000011011000010111010001110011001000000110000101101100011101110110000101111
00100110011001000000110100101101110011011100110111101100011011001010110111001
11010000100000011000100110010101100011011000010111010101110011011001010010000
00110010001101111011001110111001100100000011010000110000101110100011001010010
00000111010001101000011001010110110100100000011101110110100101110100011010000
11011110111010101110100001000000100000101000010010000110010000001110010011001
01011000010111001101101111011011100111001100101110
01000011011000010111010001110011001000000110000101101100011101110110000101111
00101010011001000000110100101101110011011100110111101100011011001010110111001
11010000100000011000100110010101100011011000010111010101110011011001010010000
00110010001101111011001110111001100100000011010000110000101110100011001010010
00000111010001101000011001010110110100100000011101110110100101110100011010000
11011110111010101110100001000000100000101000010010000110010000001110010011001
01011000010111001101101111011011100111001100101110
01000011011000010111010001110011001000000110000101101100011101110110000101111
00101100011001000000110100101101110011011100110111101100011011001010110111001
11010000100000011000100110010101100011011000010111010101110011011001010010000
00110010001101111011001110111001100100000011010000110000101110100011001010010
00000111010001101000011001010110110100100000011101110110100101110100011010000
11011110111010101110100001000000100000101000010010000110010000001110010011001
01011000010111001101101111011011100111001100101110
01000011011000010111010001110011001000000110000101101100011101110110000101111
00101111011001000000110100101101110011011100110111101100011011001010110111001
11010000100000011000100110010101100011011000010111010101110011011001010010000
00110010001101111011001110111001100100000011010000110000101110100011001010010
00000111010001101000011001010110110100100000011101110110100101110100011010000

11011110111010101110100001000000100000101000010010000110010000001110010011001
01011000010111001101101111011011100111001100101110
01000011011000010111010001110011001000000110000101101100011101110110000101111
00101110111001000000011010010110111001101110011011110110001101100101011 0111001
11010000100000011000100110010101100011011000010111010101110011011001010010000
00110010001101111011001110111001100100000011010000110000101110100011001010010
00000111010001101000011001010110110100100000011101110110100101110100011010000
11011110111010101110100001000000100000101000010010000110010000001110010011001
01011000010111001101101111011011100111001100101110
01000011011000010111010001110011001000000110000101101100011101110110000101111
00101110001001000000011010010110111001101110011011110110001101100101011 0111001
11010000100000011000100110010101100011011000010111010101110011011001010010000
00110010001101111011001110111001100100000011010000110000101110100011001010010
00000111010001101000011001010110110100100000011101110110100101110100011010000
11011110111010101110100001000000100000101000010010000110010000001110010011001
01011000010111001101101111011011100111001100101110
01000011011000010111010001110011001000000110000101101100011101110110000101111
00101110010001000000011010010110111001101110011011110110001101100101011 0111001
11010000100000011000100110010101100011011000010111010101110011011001010010000
00110010001101111011001110111001100100000011010000110000101110100011001010010
00000111010001101000011001010110110100100000011101110110100101110100011010000
11011110111010101110100001000000100000101000010010000110010000001110010011001
01011000010111001101101111011011100111001100101110
01000011011000010111010001110011001000000110000101101100011101110110000101111
00101110011101000000011010010110111001101110011011110110001101100101011 0111001
11010000100000011000100110010101100011011000010111010101110011011001010010000
00110010001101111011001110111001100100000011010000110000101110100011001010010
00000111010001101000011001010110110100100000011101110110100101110100011010000
11011110111010101110100001000000100000101000010010000110010000001110010011001
01011000010111001101101111011011100111001100101110
01000011011000010111010001110011001000000110000101101100011101110110000101111
00101110011011000000011010010110111001101110011011110110001101100101011 0111001
11010000100000011000100110010101100011011000010111010101110011011001010010000
00110010001101111011001110111001100100000011010000110000101110100011001010010
00000111010001101000011001010110110100100000011101110110100101110100011010000
11011110111010101110100001000000100000101000010010000110010000001110010011001
01011000010111001101101111011011100111001100101110
01000011011000010111010001110011001000000110000101101100011101110110000101111
00101110011000000000011010010110111001101110011011110110001101100101011 0111001
11010000100000011000100110010101100011011000010111010101110011011001010010000
00110010001101111011001110111001100100000011010000110000101110100011001010010
00000111010001101000011001010110110100100000011101110110100101110100011010000
11011110111010101110100001000000100000101000010010000110010000001110010011001
01011000010111001101101111011011100111001100101110
01000011011000010111010001110011001000000110000101101100011101110110000101111
00101110011001100000011010010110111001101110011011110110001101100101011 0111001
11010000100000011000100110010101100011011000010111010101110011011001010010000
00110010001101111011001110111001100100000011010000110000101110100011001010010
00000111010001101000011001010110110100100000011101110110100101110100011010000
11011110111010101110100001000000100000101000010010000110010000001110010011001
01011000010111001101101111011011100111001100101110
01000011011000010111010001110011001000000110000101101100011101110110000101111
00101110011001010000011010010110111001101110011011110110001101100101011 0111001
11010000100000011000100110010101100011011000010111010101110011011001010010000
00110010001101111011001110111001100100000011010000110000101110100011001010010
00000111010001101000011001010110110100100000011101110110100101110100011010000

11011110111010101110100001000000010000001010000100100001100100000001110010011001
01011000010111001101101111011011100111001100101110
01000011011000010111010001110011001000000110000101101100011101110110000101111
00101110011001001000110100101101110011011100110111101100011011001010110111001
11010000100000011000100110010101100011011000010111010101110011011001010010000
001100100011011110110011101110011001000000011010000110000101110100011001010010
00000111010001101000011001010110110100100000011101110110100101110100011010000
11011110111010101110100001000000010000001010000100100001100100000001110010011001
01011000010111001101101111011011100111001100101110
01000011011000010111010001110011001000000110000101101100011101110110000101111
00101110011001000100110100101101110011011100110111101100011011001010110111001
11010000100000011000100110010101100011011000010111010101110011011001010010000
001100100011011110110011101110011001000000011010000110000101110100011001010010
00000111010001101000011001010110110100100000011101110110100101110100011010000
11011110111010101110100001000000010000001010000100100001100100000001110010011001
01011000010111001101101111011011100111001100101110
01000011011000010111010001110011001000000110000101101100011101110110000101111
00101110011001000010110100101101110011011100110111101100011011001010110111001
11010000100000011000100110010101100011011000010111010101110011011001010010000
001100100011011110110011101110011001000000011010000110000101110100011001010010
00000111010001101000011001010110110100100000011101110110100101110100011010000
11011110111010101110100001000000010000001010000100100001100100000001110010011001
01011000010111001101101111011011100111001100101110
01000011011000010111010001110011001000000110000101101100011101110110000101111
001011100110010000001110100101101110011011100110111101100011011001010110111001
11010000100000011000100110010101100011011000010111010101110011011001010010000
001100100011011110110011101110011001000000011010000110000101110100011001010010
00000111010001101000011001010110110100100000011101110110100101110100011010000
11011110111010101110100001000000010000001010000100100001100100000001110010011001
01011000010111001101101111011011100111001100101110
01000011011000010111010001110011001000000110000101101100011101110110000101111
001011100110010000000010100101101110011011100110111101100011011001010110111001
11010000100000011000100110010101100011011000010111010101110011011001010010000
001100100011011110110011101110011001000000011010000110000101110100011001010010
00000111010001101000011001010110110100100000011101110110100101110100011010000
11011110111010101110100001000000010000001010000100100001100100000001110010011001
01011000010111001101101111011011100111001100101110
01000011011000010111010001110011001000000110000101101100011101110110000101111
001011100110010000000100100101101110011011100110111101100011011001010110111001
11010000100000011000100110010101100011011000010111010101110011011001010010000
001100100011011110110011101110011001000000011010000110000101110100011001010010
00000111010001101000011001010110110100100000011101110110100101110100011010000
11011110111010101110100001000000010000001010000100100001100100000001110010011001
01011000010111001101101111011011100111001100101110
01000011011000010111010001110011001000000110000101101100011101110110000101111
001011100110010000000111100101101110011011100110111101100011011001010110111001
11010000100000011000100110010101100011011000010111010101110011011001010010000
001100100011011110110011101110011001000000011010000110000101110100011001010010
00000111010001101000011001010110110100100000011101110110100101110100011010000
11011110111010101110100001000000010000001010000100100001100100000001110010011001
01011000010111001101101111011011100111001100101110
01000011011000010111010001110011001000000110000101101100011101110110000101111
001011100110010000000110000101101110011011100110111101100011011001010110111001
11010000100000011000100110010101100011011000010111010101110011011001010010000
001100100011011110110011101110011001000000011010000110000101110100011001010010
00000111010001101000011001010110110100100000011101110110100101110100011010000

11011110111010101110100001000000100000101000010010000110010000001110010011001
01011000010111001101101111011011100111001100101110
01000011011000010111010001110011001000000110000101101100011101110110000101111
00101110011001000000110110101101110011011100110111101100011011001010110111001
11010000100000011000100110010101100011011000010111010101110011011001010010000
00110010001101111011001110111001100100000011010000110000101110100011001010010
00000111010001101000011001010110110100100000011101110110100101110100011010000
11011110111010101110100001000000100000101000010010000110010000001110010011001
01011000010111001101101111011011100111001100101110
01000011011000010111010001110011001000000110000101101100011101110110000101111
00101110011001000000110101101101110011011100110111101100011011001010110111001
11010000100000011000100110010101100011011000010111010101110011011001010010000
00110010001101111011001110111001100100000011010000110000101110100011001010010
00000111010001101000011001010110110100100000011101110110100101110100011010000
11011110111010101110100001000000100000101000010010000110010000001110010011001
01011000010111001101101111011011100111001100101110
01000011011000010111010001110011001000000110000101101100011101110110000101111
00101110011001000000110100001101110011011100110111101100011011001010110111001
11010000100000011000100110010101100011011000010111010101110011011001010010000
00110010001101111011001110111001100100000011010000110000101110100011001010010
00000111010001101000011001010110110100100000011101110110100101110100011010000
11011110111010101110100001000000100000101000010010000110010000001110010011001
01011000010111001101101111011011100111001100101110
01000011011000010111010001110011001000000110000101101100011101110110000101111
00101110011001000000110100111101110011011100110111101100011011001010110111001
11010000100000011000100110010101100011011000010111010101110011011001010010000
00110010001101111011001110111001100100000011010000110000101110100011001010010
00000111010001101000011001010110110100100000011101110110100101110100011010000
11011110111010101110100001000000100000101000010010000110010000001110010011001
01011000010111001101101111011011100111001100101110
01000011011000010111010001110011001000000110000101101100011101110110000101111
00101110011001000000110100100101110011011100110111101100011011001010110111001
11010000100000011000100110010101100011011000010111010101110011011001010010000
00110010001101111011001110111001100100000011010000110000101110100011001010010
00000111010001101000011001010110110100100000011101110110100101110100011010000
11011110111010101110100001000000100000101000010010000110010000001110010011001
01011000010111001101101111011011100111001100101110
01000011011000010111010001110011001000000110000101101100011101110110000101111
00101110011001000000110100101001110011011100110111101100011011001010110111001
11010000100000011000100110010101100011011000010111010101110011011001010010000
00110010001101111011001110111001100100000011010000110000101110100011001010010
00000111010001101000011001010110110100100000011101110110100101110100011010000
11011110111010101110100001000000100000101000010010000110010000001110010011001
01011000010111001101101111011011100111001100101110
01000011011000010111010001110011001000000110000101101100011101110110000101111
00101110011001000000110100101111110011011100110111101100011011001010110111001
11010000100000011000100110010101100011011000010111010101110011011001010010000
00110010001101111011001110111001100100000011010000110000101110100011001010010
00000111010001101000011001010110110100100000011101110110100101110100011010000
11011110111010101110100001000000100000101000010010000110010000001110010011001
01011000010111001101101111011011100111001100101110
01000011011000010111010001110011001000000110000101101100011101110110000101111
00101110011001000000110100101100110011011100110111101100011011001010110111001
11010000100000011000100110010101100011011000010111010101110011011001010010000
00110010001101111011001110111001100100000011010000110000101110100011001010010
00000111010001101000011001010110110100100000011101110110100101110100011010000

11011110111010101110100001000000100000101000010010000110010000001110010011001
01011000010111001101101111011011100111001100101110
01000011011000010111010001110011001000000110000101101100011101110110000101111
00101110011001000000110100101101010011011100110111101100011011001010110111001
11010000100000011000100110010101100011011000010111010101110011011001010010000
00110010001101111011001110111001100100000011010000110000101110100011001010010
00000111010001101000011001010110110100100000011101110110100101110100011010000
11011110111010101110100001000000100000101000010010000110010000001110010011001
01011000010111001101101111011011100111001100101110
01000011011000010111010001110011001000000110000101101100011101110110000101111
00101110011001000000110100101101100011011100110111101100011011001010110111001
11010000100000011000100110010101100011011000010111010101110011011001010010000
00110010001101111011001110111001100100000011010000110000101110100011001010010
00000111010001101000011001010110110100100000011101110110100101110100011010000
11011110111010101110100001000000100000101000010010000110010000001110010011001
01011000010111001101101111011011100111001100101110
01000011011000010111010001110011001000000110000101101100011101110110000101111
00101110011001000000110100101101111011011100110111101100011011001010110111001
11010000100000011000100110010101100011011000010111010101110011011001010010000
00110010001101111011001110111001100100000011010000110000101110100011001010010
00000111010001101000011001010110110100100000011101110110100101110100011010000
11011110111010101110100001000000100000101000010010000110010000001110010011001
01011000010111001101101111011011100111001100101110
01000011011000010111010001110011001000000110000101101100011101110110000101111
00101110011001000000110100101101110111011100110111101100011011001010110111001
11010000100000011000100110010101100011011000010111010101110011011001010010000
00110010001101111011001110111001100100000011010000110000101110100011001010010
00000111010001101000011001010110110100100000011101110110100101110100011010000
11011110111010101110100001000000100000101000010010000110010000001110010011001
01011000010111001101101111011011100111001100101110
01000011011000010111010001110011001000000110000101101100011101110110000101111
00101110011001000000110100101101110001011100110111101100011011001010110111001
11010000100000011000100110010101100011011000010111010101110011011001010010000
00110010001101111011001110111001100100000011010000110000101110100011001010010
00000111010001101000011001010110110100100000011101110110100101110100011010000
11011110111010101110100001000000100000101000010010000110010000001110010011001
01011000010111001101101111011011100111001100101110
01000011011000010111010001110011001000000110000101101100011101110110000101111
00101110011001000000110100101101110010011100110111101100011011001010110111001
11010000100000011000100110010101100011011000010111010101110011011001010010000
00110010001101111011001110111001100100000011010000110000101110100011001010010
00000111010001101000011001010110110100100000011101110110100101110100011010000
11011110111010101110100001000000100000101000010010000110010000001110010011001
01011000010111001101101111011011100111001100101110
01000011011000010111010001110011001000000110000101101100011101110110000101111
00101110011001000000110100101101110011111100110111101100011011001010110111001
11010000100000011000100110010101100011011000010111010101110011011001010010000
00110010001101111011001110111001100100000011010000110000101110100011001010010
00000111010001101000011001010110110100100000011101110110100101110100011010000
11011110111010101110100001000000100000101000010010000110010000001110010011001
01011000010111001101101111011011100111001100101110
01000011011000010111010001110011001000000110000101101100011101110110000101111
00101110011001000000110100101101110011001100110111101100011011001010110111001
11010000100000011000100110010101100011011000010111010101110011011001010010000
00110010001101111011001110111001100100000011010000110000101110100011001010010
00000111010001101000011001010110110100100000011101110110100101110100011010000

110111101110101011101000010000001000000101000010010000110010000001110010011001
01011000010111001101101111011011100111001100101110
010000110110000101110100011100110010000001100001011011000111011101100001011111
001011100110010000001101001011011100110101001101111011000110110010101101110 01
11010000100000011000100110010101100011011000010111010101110011011001010010000
0011001000110111101100111011100110010000001101000011000010111010001100101001 0
00000111010001101000011001010110110100100000011101110110100101110100011010000
110111101110101011101000010000001000000101000010010000110010000001110010011001
01011000010111001101101111011011100111001100101110
010000110110000101110100011100110010000001100001011011000111011101100001011111
0010111001100100000011010010110111001101100011011110110001101100101011011100 1
11010000100000011000100110010101100011011000010111010101110011011001010010000
0011001000110111101100111011100110010000001101000011000010111010001100101001 0
00000111010001101000011001010110110100100000011101110110100101110100011010000
110111101110101011101000010000001000000101000010010000110010000001110010011001
01011000010111001101101111011011100111001100101110
010000110110000101110100011100110010000001100001011011000111011101100001011111
0010111001100100000011010010110111001101111011011110110001101100101011011100 1
11010000100000011000100110010101100011011000010111010101110011011001010010000
0011001000110111101100111011100110010000001101000011000010111010001100101001 0
00000111010001101000011001010110110100100000011101110110100101110100011010000
110111101110101011101000010000001000000101000010010000110010000001110010011001
01011000010111001101101111011011100111001100101110
010000110110000101110100011100110010000001100001011011000111011101100001011111
0010111001100100000011010010110111001101110111011110110001101100101011011100 1
11010000100000011000100110010101100011011000010111010101110011011001010010000
0011001000110111101100111011100110010000001101000011000010111010001100101001 0
00000111010001101000011001010110110100100000011101110110100101110100011010000
110111101110101011101000010000001000000101000010010000110010000001110010011001
01011000010111001101101111011011100111001100101110
010000110110000101110100011100110010000001100001011011000111011101100001011111
0010111001100100000011010010110111001101110001011110110001101100101011011100 1
11010000100000011000100110010101100011011000010111010101110011011001010010000
0011001000110111101100111011100110010000001101000011000010111010001100101001 0
00000111010001101000011001010110110100100000011101110110100101110100011010000
110111101110101011101000010000001000000101000010010000110010000001110010011001
01011000010111001101101111011011100111001100101110
010000110110000101110100011100110010000001100001011011000111011101100001011111
0010111001100100000011010010110111001101110010011110110001101100101011011100 1
11010000100000011000100110010101100011011000010111010101110011011001010010000
0011001000110111101100111011100110010000001101000011000010111010001100101001 0
00000111010001101000011001010110110100100000011101110110100101110100011010000
110111101110101011101000010000001000000101000010010000110010000001110010011001
01011000010111001101101111011011100111001100101110
010000110110000101110100011100110010000001100001011011000111011101100001011111
0010111001100100000011010010110111001101110011111110110001101100101011011100 1
11010000100000011000100110010101100011011000010111010101110011011001010010000
0011001000110111101100111011100110010000001101000011000010111010001100101001 0
00000111010001101000011001010110110100100000011101110110100101110100011010000
110111101110101011101000010000001000000101000010010000110010000001110010011001
01011000010111001101101111011011100111001100101110
010000110110000101110100011100110010000001100001011011000111011101100001011111
0010111001100100000011010010110111001101110011001110110001101100101011011100 1
11010000100000011000100110010101100011011000010111010101110011011001010010000
0011001000110111101100111011100110010000001101000011000010111010001100101001 0
00000111010001101000011001010110110100100000011101110110100101110100011010000

11011110111010101110100001000000100000101000010010000110010000001110010011001
01011000010111001101101111011011100111001100101110
01000011011000010111010001110011001000000110000101101100011101110110000101111
00101110011001000000110100101101110011011100110101101100011011001010110111001
11010000100000011000100110010101100011011000010111010101110011011001010010000
00110010001101111011001110111001100100000011010000110000101110100011001010010
00000111010001101000011001010110110100100000011101110110100101110100011010000
11011110111010101110100001000000100000101000010010000110010000001110010011001
01011000010111001101101111011011100111001100101110
01000011011000010111010001110011001000000110000101101100011101110110000101111
00101110011001000000110100101101110011011100110110101100011011001010110111001
11010000100000011000100110010101100011011000010111010101110011011001010010000
00110010001101111011001110111001100100000011010000110000101110100011001010010
00000111010001101000011001010110110100100000011101110110100101110100011010000
11011110111010101110100001000000100000101000010010000110010000001110010011001
01011000010111001101101111011011100111001100101110
01000011011000010111010001110011001000000110000101101100011101110110000101111
00101110011001000000110100101101110011011100110111001100011011001010110111001
11010000100000011000100110010101100011011000010111010101110011011001010010000
00110010001101111011001110111001100100000011010000110000101110100011001010010
00000111010001101000011001010110110100100000011101110110100101110100011010000
11011110111010101110100001000000100000101000010010000110010000001110010011001
01011000010111001101101111011011100111001100101110

File 4: "ABCDEFGHIJKLMNOPQRSTUVWXYZ0123456789abcdefghijklmnopqrstuvwxyz!!"

01000001010000100100001101000100010001010100011001000111010010000100100101001
01001001011010011000100110101001110010011110101000001010001010100100101001101
01010001010101010101100101011101011000010110010101101000110000001100010011001
00011001100110100001101010011011000110111001110000011100101100001011000100110
00110110010001100101011001100110011101101000011010010110101001101011011011000
11011010110111001101111011100000111000101110010011100110111010001110101011101
10011101110111100001111001011110100010000100100001
00000001010000100100001101000100010001010100011001000111010010000100100101001
01001001011010011000100110101001110010011110101000001010001010100100101001101
01010001010101010101100101011101011000010110010101101000110000001100010011001
00011001100110100001101010011011000110111001110000011100101100001011000100110
00110110010001100101011001100110011101101000011010010110101001101011011011000
11011010110111001101111011100000111000101110010011100110111010001110101011101
10011101110111100001111001011110100010000100100001
01100001010000100100001101000100010001010100011001000111010010000100100101001
01001001011010011000100110101001110010011110101000001010001010100100101001101
01010001010101010101100101011101011000010110010101101000110000001100010011001
00011001100110100001101010011011000110111001110000011100101100001011000100110
00110110010001100101011001100110011101101000011010010110101001101011011011000
11011010110111001101111011100000111000101110010011100110111010001110101011101
10011101110111100001111001011110100010000100100001
01010001010000100100001101000100010001010100011001000111010010000100100101001
01001001011010011000100110101001110010011110101000001010001010100100101001101
01010001010101010101100101011101011000010110010101101000110000001100010011001
00011001100110100001101010011011000110111001110000011100101100001011000100110
00110110010001100101011001100110011101101000011010010110101001101011011011000
11011010110111001101111011100000111000101110010011100110111010001110101011101
10011101110111100001111001011110100010000100100001

01001001010000100100001101000100010001010100011001000111010010000100100101001
01001001011010011000100110101001110010011110101000001010001010100100101001101
01010001010101010101100101011101011000010110010101101000110000001100010011001
00011001100110100001101010011011000110111001110000011100101100001011000100110
00110110010001100101011001100110011101101000011010010110101001101011011011000
11011010110111001101111011100000111000101110010011100110111010001110101011101
10011101110111100001111001011110100010000100100001
01000101010000100100001101000100010001010100011001000111010010000100100101001
01001001011010011000100110101001110010011110101000001010001010100100101001101
01010001010101010101100101011101011000010110010101101000110000001100010011001
00011001100110100001101010011011000110111001110000011100101100001011000100110
00110110010001100101011001100110011101101000011010010110101001101011011011000
11011010110111001101111011100000111000101110010011100110111010001110101011101
10011101110111100001111001011110100010000100100001
01000011010000100100001101000100010001010100011001000111010010000100100101001
01001001011010011000100110101001110010011110101000001010001010100100101001101
01010001010101010101100101011101011000010110010101101000110000001100010011001
00011001100110100001101010011011000110111001110000011100101100001011000100110
00110110010001100101011001100110011101101000011010010110101001101011011011000
11011010110111001101111011100000111000101110010011100110111010001110101011101
10011101110111100001111001011110100010000100100001
01000000010000100100001101000100010001010100011001000111010010000100100101001
01001001011010011000100110101001110010011110101000001010001010100100101001101
01010001010101010101100101011101011000010110010101101000110000001100010011001
00011001100110100001101010011011000110111001110000011100101100001011000100110
00110110010001100101011001100110011101101000011010010110101001101011011011000
11011010110111001101111011100000111000101110010011100110111010001110101011101
10011101110111100001111001011110100010000100100001
01000001110000100100001101000100010001010100011001000111010010000100100101001
01001001011010011000100110101001110010011110101000001010001010100100101001101
01010001010101010101100101011101011000010110010101101000110000001100010011001
00011001100110100001101010011011000110111001110000011100101100001011000100110
00110110010001100101011001100110011101101000011010010110101001101011011011000
11011010110111001101111011100000111000101110010011100110111010001110101011101
10011101110111100001111001011110100010000100100001
01000001000000100100001101000100010001010100011001000111010010000100100101001
01001001011010011000100110101001110010011110101000001010001010100100101001101
01010001010101010101100101011101011000010110010101101000110000001100010011001
00011001100110100001101010011011000110111001110000011100101100001011000100110
00110110010001100101011001100110011101101000011010010110101001101011011011000
11011010110111001101111011100000111000101110010011100110111010001110101011101
10011101110111100001111001011110100010000100100001
01000001011000100100001101000100010001010100011001000111010010000100100101001
01001001011010011000100110101001110010011110101000001010001010100100101001101
01010001010101010101100101011101011000010110010101101000110000001100010011001
00011001100110100001101010011011000110111001110000011100101100001011000100110
00110110010001100101011001100110011101101000011010010110101001101011011011000
11011010110111001101111011100000111000101110010011100110111010001110101011101
10011101110111100001111001011110100010000100100001
01000001010100100100001101000100010001010100011001000111010010000100100101001
01001001011010011000100110101001110010011110101000001010001010100100101001101
01010001010101010101100101011101011000010110010101101000110000001100010011001
00011001100110100001101010011011000110111001110000011100101100001011000100110
00110110010001100101011001100110011101101000011010010110101001101011011011000
11011010110111001101111011100000111000101110010011100110111010001110101011101
10011101110111100001111001011110100010000100100001

01000001010010100100001101000100010001010100011001000111010010000100100101001
01001001011010011000100110101001110010011110101000001010001010100100101001101
01010001010101010101100101011101011000010110010101101000110000001100010011001
00011001100110100001101010011011000110111001110000011100101100001011000100110
00110110010001100101011001100110011101101000011010010110101001101011011011000
11011010110111001101111011100000111000101110010011100110111010001110101011101
10011101110111100001111001011110100010000100100001
01000001010001100100001101000100010001010100011001000111010010000100100101001
01001001011010011000100110101001110010011110101000001010001010100100101001101
01010001010101010101100101011101011000010110010101101000110000001100010011001
00011001100110100001101010011011000110111001110000011100101100001011000100110
00110110010001100101011001100110011101101000011010010110101001101011011011000
11011010110111001101111011100000111000101110010011100110111010001110101011101
10011101110111100001111001011110100010000100100001
01000001010000000100001101000100010001010100011001000111010010000100100101001
01001001011010011000100110101001110010011110101000001010001010100100101001101
01010001010101010101100101011101011000010110010101101000110000001100010011001
00011001100110100001101010011011000110111001110000011100101100001011000100110
00110110010001100101011001100110011101101000011010010110101001101011011011000
11011010110111001101111011100000111000101110010011100110111010001110101011101
10011101110111100001111001011110100010000100100001
01000001010000110100001101000100010001010100011001000111010010000100100101001
01001001011010011000100110101001110010011110101000001010001010100100101001101
01010001010101010101100101011101011000010110010101101000110000001100010011001
00011001100110100001101010011011000110111001110000011100101100001011000100110
00110110010001100101011001100110011101101000011010010110101001101011011011000
11011010110111001101111011100000111000101110010011100110111010001110101011101
10011101110111100001111001011110100010000100100001
01000001010000101100001101000100010001010100011001000111010010000100100101001
01001001011010011000100110101001110010011110101000001010001010100100101001101
01010001010101010101100101011101011000010110010101101000110000001100010011001
00011001100110100001101010011011000110111001110000011100101100001011000100110
00110110010001100101011001100110011101101000011010010110101001101011011011000
11011010110111001101111011100000111000101110010011100110111010001110101011101
10011101110111100001111001011110100010000100100001
01000001010000100000001101000100010001010100011001000111010010000100100101001
01001001011010011000100110101001110010011110101000001010001010100100101001101
01010001010101010101100101011101011000010110010101101000110000001100010011001
00011001100110100001101010011011000110111001110000011100101100001011000100110
00110110010001100101011001100110011101101000011010010110101001101011011011000
11011010110111001101111011100000111000101110010011100110111010001110101011101
10011101110111100001111001011110100010000100100001
01000001010000100110001101000100010001010100011001000111010010000100100101001
01001001011010011000100110101001110010011110101000001010001010100100101001101
01010001010101010101100101011101011000010110010101101000110000001100010011001
00011001100110100001101010011011000110111001110000011100101100001011000100110
00110110010001100101011001100110011101101000011010010110101001101011011011000
11011010110111001101111011100000111000101110010011100110111010001110101011101
10011101110111100001111001011110100010000100100001
01000001010000100101001101000100010001010100011001000111010010000100100101001
01001001011010011000100110101001110010011110101000001010001010100100101001101
01010001010101010101100101011101011000010110010101101000110000001100010011001
00011001100110100001101010011011000110111001110000011100101100001011000100110
00110110010001100101011001100110011101101000011010010110101001101011011011000
11011010110111001101111011100000111000101110010011100110111010001110101011101
10011101110111100001111001011110100010000100100001

0100000101000001001001011010001000100010101000110010001110100100001001001010 01
01001001011010011000100110101001110010011110101000001010001010100100101001101
01010001010101010101100101011101011000010110010101101000110000001100010011001
00011001100110100001101010011011000110111001110000011100101100001011000100110
00110110010001100101011001100110011101101000011010010110101001101011011011000
11011010110111001101111011100000111000101110010011100110111010001110101011101
10011101110111100001111001011110100010000100100001
0100000101000001001000111010001000100010101000110010001110100100001001001010 01
01001001011010011000100110101001110010011110101000001010001010100100101001101
01010001010101010101100101011101011000010110010101101000110000001100010011001
00011001100110100001101010011011000110111001110000011100101100001011000100110
00110110010001100101011001100110011101101000011010010110101001101011011011000
11011010110111001101111011100000111000101110010011100110111010001110101011101
10011101110111100001111001011110100010000100100001
0100000101000001001000001010001000100010101000110010001110100100001001001010 01
01001001011010011000100110101001110010011110101000001010001010100100101001101
01010001010101010101100101011101011000010110010101101000110000001100010011001
00011001100110100001101010011011000110111001110000011100101100001011000100110
00110110010001100101011001100110011101101000011010010110101001101011011011000
11011010110111001101111011100000111000101110010011100110111010001110101011101
10011101110111100001111001011110100010000100100001
0100000101000001001000010010001000100010101000110010001110100100001001001010 01
01001001011010011000100110101001110010011110101000001010001010100100101001101
01010001010101010101100101011101011000010110010101101000110000001100010011001
00011001100110100001101010011011000110111001110000011100101100001011000100110
00110110010001100101011001100110011101101000011010010110101001101011011011000
11011010110111001101111011100000111000101110010011100110111010001110101011101
10011101110111100001111001011110100010000100100001
0100000101000001001000011110001000100010101000110010001110100100001001001010 01
01001001011010011000100110101001110010011110101000001010001010100100101001101
01010001010101010101100101011101011000010110010101101000110000001100010011001
00011001100110100001101010011011000110111001110000011100101100001011000100110
00110110010001100101011001100110011101101000011010010110101001101011011011000
11011010110111001101111011100000111000101110010011100110111010001110101011101
10011101110111100001111001011110100010000100100001
0100000101000001001000011000001000100010101000110010001110100100001001001010 01
01001001011010011000100110101001110010011110101000001010001010100100101001101
01010001010101010101100101011101011000010110010101101000110000001100010011001
00011001100110100001101010011011000110111001110000011100101100001011000100110
00110110010001100101011001100110011101101000011010010110101001101011011011000
11011010110111001101111011100000111000101110010011100110111010001110101011101
10011101110111100001111001011110100010000100100001
0100000101000001001000011011001000100010101000110010001110100100001001001010 01
01001001011010011000100110101001110010011110101000001010001010100100101001101
01010001010101010101100101011101011000010110010101101000110000001100010011001
00011001100110100001101010011011000110111001110000011100101100001011000100110
00110110010001100101011001100110011101101000011010010110101001101011011011000
11011010110111001101111011100000111000101110010011100110111010001110101011101
10011101110111100001111001011110100010000100100001
0100000101000001001000011010101000100010101000110010001110100100001001001010 01
01001001011010011000100110101001110010011110101000001010001010100100101001101
01010001010101010101100101011101011000010110010101101000110000001100010011001
00011001100110100001101010011011000110111001110000011100101100001011000100110
00110110010001100101011001100110011101101000011010010110101001101011011011000
11011010110111001101111011100000111000101110010011100110111010001110101011101
10011101110111100001111001011110100010000100100001

0100000101000001001000001101001100010001010100011001000111010010000100100101001
01001001011010011000100110101001110010011110101000001010001010100100101001101
0101000101010101010101100101011101011000010110010101101000110000001100010011001
000110011001101000011010100110110001101110011100000011100101100001011000100110
00110110010001100101011001100110011101101000011010010110101001101011011011000
11011010110111001101111011100000111000101110010011100110111010001110101011101
10011101110111100001111001011110100010000100100001
01000001010000010010000011010000000010001010100011001000111010010000100100101001
01001001011010011000100110101001110010011110101000001010001010100100101001101
0101000101010101010101100101011101011000010110010101101000110000001100010011001
000110011001101000011010100110110001101110011100000011100101100001011000100110
00110110010001100101011001100110011101101000011010010110101001101011011011000
11011010110111001101111011100000111000101110010011100110111010001110101011101
10011101110111100001111001011110100010000100100001
0100000101000001001000001101000110010001010100011001000111010010000100100101001
01001001011010011000100110101001110010011110101000001010001010100100101001101
0101000101010101010101100101011101011000010110010101101000110000001100010011001
000110011001101000011010100110110001101110011100000011100101100001011000100110
00110110010001100101011001100110011101101000011010010110101001101011011011000
11011010110111001101111011100000111000101110010011100110111010001110101011101
10011101110111100001111001011110100010000100100001
0100000101000001001000001101000101010001010100011001000111010010000100100101001
01001001011010011000100110101001110010011110101000001010001010100100101001101
0101000101010101010101100101011101011000010110010101101000110000001100010011001
000110011001101000011010100110110001101110011100000011100101100001011000100110
00110110010001100101011001100110011101101000011010010110101001101011011011000
11011010110111001101111011100000111000101110010011100110111010001110101011101
10011101110111100001111001011110100010000100100001
0100000101000001001000001101000100110001010100011001000111010010000100100101001
01001001011010011000100110101001110010011110101000001010001010100100101001101
0101000101010101010101100101011101011000010110010101101000110000001100010011001
000110011001101000011010100110110001101110011100000011100101100001011000100110
00110110010001100101011001100110011101101000011010010110101001101011011011000
11011010110111001101111011100000111000101110010011100110111010001110101011101
10011101110111100001111001011110100010000100100001
01000001010000010010000011010001000000001010100011001000111010010000100100101001
01001001011010011000100110101001110010011110101000001010001010100100101001101
0101000101010101010101100101011101011000010110010101101000110000001100010011001
000110011001101000011010100110110001101110011100000011100101100001011000100110
00110110010001100101011001100110011101101000011010010110101001101011011011000
11011010110111001101111011100000111000101110010011100110111010001110101011101
10011101110111100001111001011110100010000100100001
0100000101000001001000001101000100010101010100011001000111010010000100100101001
01001001011010011000100110101001110010011110101000001010001010100100101001101
0101000101010101010101100101011101011000010110010101101000110000001100010011001
000110011001101000011010100110110001101110011100000011100101100001011000100110
00110110010001100101011001100110011101101000011010010110101001101011011011000
11011010110111001101111011100000111000101110010011100110111010001110101011101
10011101110111100001111001011110100010000100100001

0100000101000001001000001101000100010011010100011001000111010010000100100101001
0100100101101001100010011010100111001001111010100000101000101010010010100110 1
0101000101010101010110010101110101100001011001010110100011000000110001001100 1
0001100110011010000110101001101100011011100111000001110010110000101100010011 0
0011011001000110010101100110011001110110100001101001011010100110101101101100 0
1101101011011100110111101110000011100010111001001110011011101000111010101110 1
1001110111011110000111100101111010001000010010000 1
0100000101000001001000001101000100010000010100011001000111010010000100100101001
01001001011010011000100110101001110010011110101000001010001010100100101001101
01010001010101010101100101011101011000010110010101101000110000001100010011001
00011001100110100001101010011011000110111001110000011100101100001011000100110
00110110010001100101011001100110011101101000011010010110101001101011011011000
11011010110111001101111011100000111000101110010011100110111010001110101011101
10011101110111100001111001011110100010000100100001
0100000101000001001000001101000100010001110100011001000111010010000100100101001
01001001011010011000100110101001110010011110101000001010001010100100101001101
01010001010101010101100101011101011000010110010101101000110000001100010011001
00011001100110100001101010011011000110111001110000011100101100001011000100110
00110110010001100101011001100110011101101000011010010110101001101011011011000
11011010110111001101111011100000111000101110010011100110111010001110101011101
10011101110111100001111001011110100010000100100001
0100000101000001001000001101000100010001000100011001000111010010000100100101001
01001001011010011000100110101001110010011110101000001010001010100100101001101
01010001010101010101100101011101011000010110010101101000110000001100010011001
00011001100110100001101010011011000110111001110000011100101100001011000100110
00110110010001100101011001100110011101101000011010010110101001101011011011000
11011010110111001101111011100000111000101110010011100110111010001110101011101
10011101110111100001111001011110100010000100100001
0100000101000001001000001101000100010001011100011001000111010010000100100101001
01001001011010011000100110101001110010011110101000001010001010100100101001101
01010001010101010101100101011101011000010110010101101000110000001100010011001
00011001100110100001101010011011000110111001110000011100101100001011000100110
00110110010001100101011001100110011101101000011010010110101001101011011011000
11011010110111001101111011100000111000101110010011100110111010001110101011101
10011101110111100001111001011110100010000100100001
0100000101000001001000001101000100010001010000011001000111010010000100100101001
01001001011010011000100110101001110010011110101000001010001010100100101001101
01010001010101010101100101011101011000010110010101101000110000001100010011001
00011001100110100001101010011011000110111001110000011100101100001011000100110
00110110010001100101011001100110011101101000011010010110101001101011011011000
11011010110111001101111011100000111000101110010011100110111010001110101011101
10011101110111100001111001011110100010000100100001
0100000101000001001000001101000100010001010110011001000111010010000100100101001
01001001011010011000100110101001110010011110101000001010001010100100101001101
01010001010101010101100101011101011000010110010101101000110000001100010011001
00011001100110100001101010011011000110111001110000011100101100001011000100110
00110110010001100101011001100110011101101000011010010110101001101011011011000
11011010110111001101111011100000111000101110010011100110111010001110101011101
10011101110111100001111001011110100010000100100001
0100000101000001001000001101000100010001010101011001000111010010000100100101001
01001001011010011000100110101001110010011110101000001010001010100100101001101
01010001010101010101100101011101011000010110010101101000110000001100010011001
00011001100110100001101010011011000110111001110000011100101100001011000100110
00110110010001100101011001100110011101101000011010010110101001101011011011000
11011010110111001101111011100000111000101110010011100110111010001110101011101
10011101110111100001111001011110100010000100100001

010000010100000100100001101000100010001010100111001000111010010000100100101001
010010010110100110001001101010011100100111101010000001010001010100100101001101
0101000101010101010101100101011101011000010110010101101000110000001100010011001
000110011001101000011010100110110001101110011100000011100101100001011000100110
00110110010001100101011001100110011101101000011010010110101001101011011011000
11011010110111001101111011100000111000101110010011100110111010001110101011101
1001110111011110000111100101111101000100000100100001
010000010100000100100001101000100010001010100001001000111010010000100100101001
010010010110100110001001101010011100100111101010000001010001010100100101001101
0101000101010101010101100101011101011000010110010101101000110000001100010011001
000110011001101000011010100110110001101110011100000011100101100001011000100110
00110110010001100101011001100110011101101000011010010110101001101011011011000
11011010110111001101111011100000111000101110010011100110111010001110101011101
1001110111011110000111100101111101000100000100100001
010000010100000100100001101000100010001010100010001000111010010000100100101001
010010010110100110001001101010011100100111101010000001010001010100100101001101
0101000101010101010101100101011101011000010110010101101000110000001100010011001
000110011001101000011010100110110001101110011100000011100101100001011000100110
00110110010001100101011001100110011101101000011010010110101001101011011011000
11011010110111001101111011100000111000101110010011100110111010001110101011101
1001110111011110000111100101111101000100000100100001
010000010100000100100001101000100010001010100011101000111010010000100100101001
010010010110100110001001101010011100100111101010000001010001010100100101001101
0101000101010101010101100101011101011000010110010101101000110000001100010011001
000110011001101000011010100110110001101110011100000011100101100001011000100110
00110110010001100101011001100110011101101000011010010110101001101011011011000
11011010110111001101111011100000111000101110010011100110111010001110101011101
1001110111011110000111100101111101000100000100100001
010000010100000100100001101000100010001010100011011000111010010000100100101001
010010010110100110001001101010011100100111101010000001010001010100100101001101
0101000101010101010101100101011101011000010110010101101000110000001100010011001
000110011001101000011010100110110001101110011100000011100101100001011000100110
00110110010001100101011001100110011101101000011010010110101001101011011011000
11011010110111001101111011100000111000101110010011100110111010001110101011101
1001110111011110000111100101111101000100000100100001
010000010100000100100001101000100010001010100011000000111010010000100100101001
010010010110100110001001101010011100100111101010000001010001010100100101001101
0101000101010101010101100101011101011000010110010101101000110000001100010011001
000110011001101000011010100110110001101110011100000011100101100001011000100110
00110110010001100101011001100110011101101000011010010110101001101011011011000
11011010110111001101111011100000111000101110010011100110111010001110101011101
1001110111011110000111100101111101000100000100100001
010000010100000100100001101000100010001010100011001100111010010000100100101001
010010010110100110001001101010011100100111101010000001010001010100100101001101
0101000101010101010101100101011101011000010110010101101000110000001100010011001
000110011001101000011010100110110001101110011100000011100101100001011000100110
00110110010001100101011001100110011101101000011010010110101001101011011011000
11011010110111001101111011100000111000101110010011100110111010001110101011101
1001110111011110000111100101111101000100000100100001
010000010100000100100001101000100010001010100011001010111010010000100100101001
010010010110100110001001101010011100100111101010000001010001010100100101001101
0101000101010101010101100101011101011000010110010101101000110000001100010011001
000110011001101000011010100110110001101110011100000011100101100001011000100110
00110110010001100101011001100110011101101000011010010110101001101011011011000
11011010110111001101111011100000111000101110010011100110111010001110101011101
1001110111011110000111100101111101000100000100100001

01000001010000010010000011010001000100010101000110010011110100100001001001010 01
01001001011010011000100110101001110010011110101000000101000101010010010100 1101
01010001010101010101100101011101011000010110010101101000110000001100010011001
000110011001101000011010100110110001101110011100000011100101100001011000100110
00110110010001100101011001100110011101101000011010010110101001101011011011000
11011010110111001101111011100000111000101110010011100110111010001110101011101
10011101110111100001111001011110100010000100100001
0100000101000001001000001101000100010001010100011001000011010010000100100101001
01001001011010011000100110101001110010011110101000000101000101010010010100 1101
01010001010101010101100101011101011000010110010101101000110000001100010011001
000110011001101000011010100110110001101110011100000011100101100001011000100110
00110110010001100101011001100110011101101000011010010110101001101011011011000
11011010110111001101111011100000111000101110010011100110111010001110101011101
10011101110111100001111001011110100010000100100001
0100000101000001001000001101000100010001010100011001000101010010000100100101001
01001001011010011000100110101001110010011110101000000101000101010010010100 1101
01010001010101010101100101011101011000010110010101101000110000001100010011001
000110011001101000011010100110110001101110011100000011100101100001011000100110
00110110010001100101011001100110011101101000011010010110101001101011011011000
11011010110111001101111011100000111000101110010011100110111010001110101011101
10011101110111100001111001011110100010000100100001
0100000101000001001000001101000100010001010100011001000110010010000100100101001
01001001011010011000100110101001110010011110101000000101000101010010010100 1101
01010001010101010101100101011101011000010110010101101000110000001100010011001
000110011001101000011010100110110001101110011100000011100101100001011000100110
00110110010001100101011001100110011101101000011010010110101001101011011011000
11011010110111001101111011100000111000101110010011100110111010001110101011101
10011101110111100001111001011110100010000100100001
0100000101000001001000001101000100010001010100011001000111110010000100100101001
01001001011010011000100110101001110010011110101000000101000101010010010100 1101
01010001010101010101100101011101011000010110010101101000110000001100010011001
000110011001101000011010100110110001101110011100000011100101100001011000100110
00110110010001100101011001100110011101101000011010010110101001101011011011000
11011010110111001101111011100000111000101110010011100110111010001110101011101
10011101110111100001111001011110100010000100100001
0100000101000001001000001101000100010001010100011001000111000010000100100101001
01001001011010011000100110101001110010011110101000000101000101010010010100 1101
01010001010101010101100101011101011000010110010101101000110000001100010011001
000110011001101000011010100110110001101110011100000011100101100001011000100110
00110110010001100101011001100110011101101000011010010110101001101011011011000
11011010110111001101111011100000111000101110010011100110111010001110101011101
10011101110111100001111001011110100010000100100001
0100000101000001001000001101000100010001010100011001000111011010000100100101001
01001001011010011000100110101001110010011110101000000101000101010010010100 1101
01010001010101010101100101011101011000010110010101101000110000001100010011001
000110011001101000011010100110110001101110011100000011100101100001011000100110
00110110010001100101011001100110011101101000011010010110101001101011011011000
11011010110111001101111011100000111000101110010011100110111010001110101011101
10011101110111100001111001011110100010000100100001
0100000101000001001000001101000100010001010100011001000111010110000100100101001
01001001011010011000100110101001110010011110101000000101000101010010010100 1101
01010001010101010101100101011101011000010110010101101000110000001100010011001
000110011001101000011010100110110001101110011100000011100101100001011000100110
00110110010001100101011001100110011101101000011010010110101001101011011011000
11011010110111001101111011100000111000101110010011100110111010001110101011101
10011101110111100001111001011110100010000100100001

010000010100000100100001101000100010001010100011001000111010000000100100101001
01001001011010011000100110101001110010011110101000001010001010100100101001101
01010001010101010101100101011101011000010110010101101000110000001100010011001
00011001100110100001101010011011000110111001110000011100101100001011000100110
00110110010001100101011001100110011101101000011010010110101001101011011011000
11011010110111001101111011100000111000101110010011100110111010001110101011101
10011101110111100001111001011110100010000100100001
010000010100000100100001101000100010001010100011001000111010011000100100101001
01001001011010011000100110101001110010011110101000001010001010100100101001101
01010001010101010101100101011101011000010110010101101000110000001100010011001
00011001100110100001101010011011000110111001110000011100101100001011000100110
00110110010001100101011001100110011101101000011010010110101001101011011011000
11011010110111001101111011100000111000101110010011100110111010001110101011101
10011101110111100001111001011110100010000100100001
010000010100000100100001101000100010001010100011001000111010010100100100101001
01001001011010011000100110101001110010011110101000001010001010100100101001101
01010001010101010101100101011101011000010110010101101000110000001100010011001
00011001100110100001101010011011000110111001110000011100101100001011000100110
00110110010001100101011001100110011101101000011010010110101001101011011011000
11011010110111001101111011100000111000101110010011100110111010001110101011101
10011101110111100001111001011110100010000100100001
010000010100000100100001101000100010001010100011001000111010010010100100101001
01001001011010011000100110101001110010011110101000001010001010100100101001101
01010001010101010101100101011101011000010110010101101000110000001100010011001
00011001100110100001101010011011000110111001110000011100101100001011000100110
00110110010001100101011001100110011101101000011010010110101001101011011011000
11011010110111001101111011100000111000101110010011100110111010001110101011101
10011101110111100001111001011110100010000100100001
010000010100000100100001101000100010001010100011001000111010010001100100101001
01001001011010011000100110101001110010011110101000001010001010100100101001101
01010001010101010101100101011101011000010110010101101000110000001100010011001
00011001100110100001101010011011000110111001110000011100101100001011000100110
00110110010001100101011001100110011101101000011010010110101001101011011011000
11011010110111001101111011100000111000101110010011100110111010001110101011101
10011101110111100001111001011110100010000100100001
010000010100000100100001101000100010001010100011001000111010010000000100101001
01001001011010011000100110101001110010011110101000001010001010100100101001101
01010001010101010101100101011101011000010110010101101000110000001100010011001
00011001100110100001101010011011000110111001110000011100101100001011000100110
00110110010001100101011001100110011101101000011010010110101001101011011011000
11011010110111001101111011100000111000101110010011100110111010001110101011101
10011101110111100001111001011110100010000100100001
010000010100000100100001101000100010001010100011001000111010010000110100101001
01001001011010011000100110101001110010011110101000001010001010100100101001101
01010001010101010101100101011101011000010110010101101000110000001100010011001
00011001100110100001101010011011000110111001110000011100101100001011000100110
00110110010001100101011001100110011101101000011010010110101001101011011011000
11011010110111001101111011100000111000101110010011100110111010001110101011101
10011101110111100001111001011110100010000100100001
010000010100000100100001101000100010001010100011001000111010010000101100101001
01001001011010011000100110101001110010011110101000001010001010100100101001101
01010001010101010101100101011101011000010110010101101000110000001100010011001
00011001100110100001101010011011000110111001110000011100101100001011000100110
00110110010001100101011001100110011101101000011010010110101001101011011011000
11011010110111001101111011100000111000101110010011100110111010001110101011101
10011101110111100001111001011110100010000100100001

010000010100000100100001101000100010001010100011001000111010010000100000101001
01001001011010011000100110101001110010011110101000001010001010100100101001101
01010001010101010101100101011101011000010110010101101000110000001100010011001
00011001100110100001101010011011000110111001110000011100101100001011000100110
00110110010001100101011001100110011101101000011010010110101001101011011011000
11011010110111001101111011100000111000101110010011100110111010001110101011101
10011101110111100001111001011110100010000100100001
010000010100000100100001101000100010001010100011001000111010010000100110101001
01001001011010011000100110101001110010011110101000001010001010100100101001101
01010001010101010101100101011101011000010110010101101000110000001100010011001
00011001100110100001101010011011000110111001110000011100101100001011000100110
00110110010001100101011001100110011101101000011010010110101001101011011011000
11011010110111001101111011100000111000101110010011100110111010001110101011101
10011101110111100001111001011110100010000100100001
010000010100000100100001101000100010001010100011001000111010010000100101101001
01001001011010011000100110101001110010011110101000001010001010100100101001101
01010001010101010101100101011101011000010110010101101000110000001100010011001
00011001100110100001101010011011000110111001110000011100101100001011000100110
00110110010001100101011001100110011101101000011010010110101001101011011011000
11011010110111001101111011100000111000101110010011100110111010001110101011101
10011101110111100001111001011110100010000100100001
010000010100000100100001101000100010001010100011001000111010010000100100001001
01001001011010011000100110101001110010011110101000001010001010100100101001101
01010001010101010101100101011101011000010110010101101000110000001100010011001
00011001100110100001101010011011000110111001110000011100101100001011000100110
00110110010001100101011001100110011101101000011010010110101001101011011011000
11011010110111001101111011100000111000101110010011100110111010001110101011101
10011101110111100001111001011110100010000100100001
010000010100000100100001101000100010001010100011001000111010010000100100111001
01001001011010011000100110101001110010011110101000001010001010100100101001101
01010001010101010101100101011101011000010110010101101000110000001100010011001
00011001100110100001101010011011000110111001110000011100101100001011000100110
00110110010001100101011001100110011101101000011010010110101001101011011011000
11011010110111001101111011100000111000101110010011100110111010001110101011101
10011101110111100001111001011110100010000100100001
010000010100000100100001101000100010001010100011001000111010010000100100100001
01001001011010011000100110101001110010011110101000001010001010100100101001101
01010001010101010101100101011101011000010110010101101000110000001100010011001
00011001100110100001101010011011000110111001110000011100101100001011000100110
00110110010001100101011001100110011101101000011010010110101001101011011011000
11011010110111001101111011100000111000101110010011100110111010001110101011101
10011101110111100001111001011110100010000100100001
010000010100000100100001101000100010001010100011001000111010010000100100101101
01001001011010011000100110101001110010011110101000001010001010100100101001101
01010001010101010101100101011101011000010110010101101000110000001100010011001
00011001100110100001101010011011000110111001110000011100101100001011000100110
00110110010001100101011001100110011101101000011010010110101001101011011011000
11011010110111001101111011100000111000101110010011100110111010001110101011101
10011101110111100001111001011110100010000100100001
010000010100000100100001101000100010001010100011001000111010010000100100101011
01001001011010011000100110101001110010011110101000001010001010100100101001101
01010001010101010101100101011101011000010110010101101000110000001100010011001
00011001100110100001101010011011000110111001110000011100101100001011000100110
00110110010001100101011001100110011101101000011010010110101001101011011011000
11011010110111001101111011100000111000101110010011100110111010001110101011101
10011101110111100001111001011110100010000100100001

01000001010000010010000011010001000100010101000110010001110100100000100100101000
01001001011010011000100110101001110010011110101000001010001010100100101001101
01010001010101010101100101011101011000010110010101101000110000001100010011001
00011001100110100001101010011011000110111001110000011100101100001011000100110
00110110010001100101011001100110011101101000011010010110101001101011011011000
11011010110111001101111011100000111000101110010011100110111010001110101011101
10011101110111100001111001011110100010000100100001
01000001010000010010000011010001000100010101000110010001110100100000100100101001
11001001011010011000100110101001110010011110101000001010001010100100101001101
01010001010101010101100101011101011000010110010101101000110000001100010011001
00011001100110100001101010011011000110111001110000011100101100001011000100110
00110110010001100101011001100110011101101000011010010110101001101011011011000
11011010110111001101111011100000111000101110010011100110111010001110101011101
10011101110111100001111001011110100010000100100001
01000001010000010010000011010001000100010101000110010001110100100000100100101001
00001001011010011000100110101001110010011110101000001010001010100100101001101
01010001010101010101100101011101011000010110010101101000110000001100010011001
00011001100110100001101010011011000110111001110000011100101100001011000100110
00110110010001100101011001100110011101101000011010010110101001101011011011000
11011010110111001101111011100000111000101110010011100110111010001110101011101
10011101110111100001111001011110100010000100100001
01000001010000010010000011010001000100010101000110010001110100100000100100101001
01101001011010011000100110101001110010011110101000001010001010100100101001101
01010001010101010101100101011101011000010110010101101000110000001100010011001
00011001100110100001101010011011000110111001110000011100101100001011000100110
00110110010001100101011001100110011101101000011010010110101001101011011011000
11011010110111001101111011100000111000101110010011100110111010001110101011101
10011101110111100001111001011110100010000100100001
01000001010000010010000011010001000100010101000110010001110100100000100100101001
01011001011010011000100110101001110010011110101000001010001010100100101001101
01010001010101010101100101011101011000010110010101101000110000001100010011001
00011001100110100001101010011011000110111001110000011100101100001011000100110
00110110010001100101011001100110011101101000011010010110101001101011011011000
11011010110111001101111011100000111000101110010011100110111010001110101011101
10011101110111100001111001011110100010000100100001
01000001010000010010000011010001000100010101000110010001110100100000100100101001
01000001011010011000100110101001110010011110101000001010001010100100101001101
01010001010101010101100101011101011000010110010101101000110000001100010011001
00011001100110100001101010011011000110111001110000011100101100001011000100110
00110110010001100101011001100110011101101000011010010110101001101011011011000
11011010110111001101111011100000111000101110010011100110111010001110101011101
10011101110111100001111001011110100010000100100001
01000001010000010010000011010001000100010101000110010001110100100000100100101001
01001101011010011000100110101001110010011110101000001010001010100100101001101
01010001010101010101100101011101011000010110010101101000110000001100010011001
00011001100110100001101010011011000110111001110000011100101100001011000100110
00110110010001100101011001100110011101101000011010010110101001101011011011000
11011010110111001101111011100000111000101110010011100110111010001110101011101
10011101110111100001111001011110100010000100100001
01000001010000010010000011010001000100010101000110010001110100100000100100101001
01001011011010011000100110101001110010011110101000001010001010100100101001101
01010001010101010101100101011101011000010110010101101000110000001100010011001
00011001100110100001101010011011000110111001110000011100101100001011000100110
00110110010001100101011001100110011101101000011010010110101001101011011011000
11011010110111001101111011100000111000101110010011100110111010001110101011101
10011101110111100001111001011110100010000100100001

0100000101000001001000001101000100010001010100011001000111010010000100100101001
01001000011010011000100110101001110010011110101000001010001010100100101001101
01010001010101010101100101011101011000010110010101101000110000001100010011001
00011001100110100001101010011011000110111001110000011100101100001011000100110
00110110010001100101011001100110011101101000011010010110101001101011011011000
11011010110111001101111011100000111000101110010011100110111010001110101011101
10011101110111100001111001011110100010000100100001
0100000101000001001000001101000100010001010100011001000111010010000100100101001
01001001111010011000100110101001110010011110101000001010001010100100101001101
01010001010101010101100101011101011000010110010101101000110000001100010011001
00011001100110100001101010011011000110111001110000011100101100001011000100110
00110110010001100101011001100110011101101000011010010110101001101011011011000
11011010110111001101111011100000111000101110010011100110111010001110101011101
10011101110111100001111001011110100010000100100001
0100000101000001001000001101000100010001010100011001000111010010000100100101001
01001001001010011000100110101001110010011110101000001010001010100100101001101
01010001010101010101100101011101011000010110010101101000110000001100010011001
00011001100110100001101010011011000110111001110000011100101100001011000100110
00110110010001100101011001100110011101101000011010010110101001101011011011000
11011010110111001101111011100000111000101110010011100110111010001110101011101
10011101110111100001111001011110100010000100100001
0100000101000001001000001101000100010001010100011001000111010010000100100101001
01001001010010011000100110101001110010011110101000001010001010100100101001101
01010001010101010101100101011101011000010110010101101000110000001100010011001
00011001100110100001101010011011000110111001110000011100101100001011000100110
00110110010001100101011001100110011101101000011010010110101001101011011011000
11011010110111001101111011100000111000101110010011100110111010001110101011101
10011101110111100001111001011110100010000100100001
0100000101000001001000001101000100010001010100011001000111010010000100100101001
01001001011110011000100110101001110010011110101000001010001010100100101001101
01010001010101010101100101011101011000010110010101101000110000001100010011001
00011001100110100001101010011011000110111001110000011100101100001011000100110
00110110010001100101011001100110011101101000011010010110101001101011011011000
11011010110111001101111011100000111000101110010011100110111010001110101011101
10011101110111100001111001011110100010000100100001
0100000101000001001000001101000100010001010100011001000111010010000100100101001
01001001011000011000100110101001110010011110101000001010001010100100101001101
01010001010101010101100101011101011000010110010101101000110000001100010011001
00011001100110100001101010011011000110111001110000011100101100001011000100110
00110110010001100101011001100110011101101000011010010110101001101011011011000
11011010110111001101111011100000111000101110010011100110111010001110101011101
10011101110111100001111001011110100010000100100001
0100000101000001001000001101000100010001010100011001000111010010000100100101001
01001001011011011000100110101001110010011110101000001010001010100100101001101
01010001010101010101100101011101011000010110010101101000110000001100010011001
00011001100110100001101010011011000110111001110000011100101100001011000100110
00110110010001100101011001100110011101101000011010010110101001101011011011000
11011010110111001101111011100000111000101110010011100110111010001110101011101
10011101110111100001111001011110100010000100100001
0100000101000001001000001101000100010001010100011001000111010010000100100101001
01001001011010111000100110101001110010011110101000001010001010100100101001101
01010001010101010101100101011101011000010110010101101000110000001100010011001
00011001100110100001101010011011000110111001110000011100101100001011000100110
00110110010001100101011001100110011101101000011010010110101001101011011011000
11011010110111001101111011100000111000101110010011100110111010001110101011101
10011101110111100001111001011110100010000100100001

0100000101000001001000001101000100010001010100011001000111010010000100100101001
01001001011010001000100110101001110010011110101000001010001010100100101001101
01010001010101010101100101011101011000010110010101101000110000001100010011001
00011001100110100001101010011011000110111001110000011100101100001011000100110
00110110010001100101011001100110011101101000011010010110101001101011011011000
11011010110111001101111011100000111000101110010011100110111010001110101011101
10011101110111100001111001011110100010000100100001
0100000101000001001000001101000100010001010100011001000111010010000100100101001
01001001011010010000100110101001110010011110101000001010001010100100101001101
01010001010101010101100101011101011000010110010101101000110000001100010011001
00011001100110100001101010011011000110111001110000011100101100001011000100110
00110110010001100101011001100110011101101000011010010110101001101011011011000
11011010110111001101111011100000111000101110010011100110111010001110101011101
10011101110111100001111001011110100010000100100001
0100000101000001001000001101000100010001010100011001000111010010000100100101001
01001001011010011100100110101001110010011110101000001010001010100100101001101
01010001010101010101100101011101011000010110010101101000110000001100010011001
00011001100110100001101010011011000110111001110000011100101100001011000100110
00110110010001100101011001100110011101101000011010010110101001101011011011000
11011010110111001101111011100000111000101110010011100110111010001110101011101
10011101110111100001111001011110100010000100100001
0100000101000001001000001101000100010001010100011001000111010010000100100101001
01001001011010011010100110101001110010011110101000001010001010100100101001101
01010001010101010101100101011101011000010110010101101000110000001100010011001
00011001100110100001101010011011000110111001110000011100101100001011000100110
00110110010001100101011001100110011101101000011010010110101001101011011011000
11011010110111001101111011100000111000101110010011100110111010001110101011101
10011101110111100001111001011110100010000100100001
0100000101000001001000001101000100010001010100011001000111010010000100100101001
01001001011010011001100110101001110010011110101000001010001010100100101001101
01010001010101010101100101011101011000010110010101101000110000001100010011001
00011001100110100001101010011011000110111001110000011100101100001011000100110
00110110010001100101011001100110011101101000011010010110101001101011011011000
11011010110111001101111011100000111000101110010011100110111010001110101011101
10011101110111100001111001011110100010000100100001
0100000101000001001000001101000100010001010100011001000111010010000100100101001
01001001011010011000000110101001110010011110101000001010001010100100101001101
01010001010101010101100101011101011000010110010101101000110000001100010011001
00011001100110100001101010011011000110111001110000011100101100001011000100110
00110110010001100101011001100110011101101000011010010110101001101011011011000
11011010110111001101111011100000111000101110010011100110111010001110101011101
10011101110111100001111001011110100010000100100001
0100000101000001001000001101000100010001010100011001000111010010000100100101001
01001001011010011000110110101001110010011110101000001010001010100100101001101
01010001010101010101100101011101011000010110010101101000110000001100010011001
00011001100110100001101010011011000110111001110000011100101100001011000100110
00110110010001100101011001100110011101101000011010010110101001101011011011000
11011010110111001101111011100000111000101110010011100110111010001110101011101
10011101110111100001111001011110100010000100100001
0100000101000001001000001101000100010001010100011001000111010010000100100101001
01001001011010011000101110101001110010011110101000001010001010100100101001101
01010001010101010101100101011101011000010110010101101000110000001100010011001
00011001100110100001101010011011000110111001110000011100101100001011000100110
00110110010001100101011001100110011101101000011010010110101001101011011011000
11011010110111001101111011100000111000101110010011100110111010001110101011101
10011101110111100001111001011110100010000100100001

0100000101000001001000001101000100010001010100011001000111010010000100100101001
01001001011010011000100010101001110010011110101000001010001010100100101001101
01010001010101010101100101011101011000010110010101101000110000001100010011001
000110011001101000011010100110110001101110011100000011100101100001011000100110
00110110010001100101011001100110011101101000011010010110101001101011011011000
11011010110111001101111011100000111000101110010011100110111010001110101011101
10011101110111100001111001011110100010000100100001
0100000101000001001000001101000100010001010100011001000111010010000100100101001
01001001011010011000100100101001110010011110101000001010001010100100101001101
01010001010101010101100101011101011000010110010101101000110000001100010011001
000110011001101000011010100110110001101110011100000011100101100001011000100110
00110110010001100101011001100110011101101000011010010110101001101011011011000
11011010110111001101111011100000111000101110010011100110111010001110101011101
10011101110111100001111001011110100010000100100001
0100000101000001001000001101000100010001010100011001000111010010000100100101001
01001001011010011000100111101001110010011110101000001010001010100100101001101
01010001010101010101100101011101011000010110010101101000110000001100010011001
000110011001101000011010100110110001101110011100000011100101100001011000100110
00110110010001100101011001100110011101101000011010010110101001101011011011000
11011010110111001101111011100000111000101110010011100110111010001110101011101
10011101110111100001111001011110100010000100100001
0100000101000001001000001101000100010001010100011001000111010010000100100101001
01001001011010011000100110001001110010011110101000001010001010100100101001101
01010001010101010101100101011101011000010110010101101000110000001100010011001
000110011001101000011010100110110001101110011100000011100101100001011000100110
00110110010001100101011001100110011101101000011010010110101001101011011011000
11011010110111001101111011100000111000101110010011100110111010001110101011101
10011101110111100001111001011110100010000100100001
0100000101000001001000001101000100010001010100011001000111010010000100100101001
01001001011010011000100110111001110010011110101000001010001010100100101001101
01010001010101010101100101011101011000010110010101101000110000001100010011001
000110011001101000011010100110110001101110011100000011100101100001011000100110
00110110010001100101011001100110011101101000011010010110101001101011011011000
11011010110111001101111011100000111000101110010011100110111010001110101011101
10011101110111100001111001011110100010000100100001
0100000101000001001000001101000100010001010100011001000111010010000100100101001
01001001011010011000100110100001110010011110101000001010001010100100101001101
01010001010101010101100101011101011000010110010101101000110000001100010011001
000110011001101000011010100110110001101110011100000011100101100001011000100110
00110110010001100101011001100110011101101000011010010110101001101011011011000
11011010110111001101111011100000111000101110010011100110111010001110101011101
10011101110111100001111001011110100010000100100001
0100000101000001001000001101000100010001010100011001000111010010000100100101001
01001001011010011000100110101101110010011110101000001010001010100100101001101
01010001010101010101100101011101011000010110010101101000110000001100010011001
000110011001101000011010100110110001101110011100000011100101100001011000100110
00110110010001100101011001100110011101101000011010010110101001101011011011000
11011010110111001101111011100000111000101110010011100110111010001110101011101
10011101110111100001111001011110100010000100100001
0100000101000001001000001101000100010001010100011001000111010010000100100101001
01001001011010011000100110101011110010011110101000001010001010100100101001101
01010001010101010101100101011101011000010110010101101000110000001100010011001
000110011001101000011010100110110001101110011100000011100101100001011000100110
00110110010001100101011001100110011101101000011010010110101001101011011011000
11011010110111001101111011100000111000101110010011100110111010001110101011101
10011101110111100001111001011110100010000100100001

01000001010000010010000011010001000100010101000110010001110100100000100100101001
01001001011010011000100110101000110010011110101000001010001010100100101001101
0101000101010101010101100101011101011000010110010101101000110000001100010011001
00011001100110100001101010011011000110111001110000011100101100001011000100110
00110110010001100101011001100110011101101000011010010110101001101011011011000
11011010110111001101111011100000111000101110010011100110111010001110101011101
10011101110111100001111001011110100010000100100001
01000001010000010010000011010001000100010101000110010001110100100000100100101001
01001001011010011000100110101001010010011110101000001010001010100100101001101
0101000101010101010101100101011101011000010110010101101000110000001100010011001
00011001100110100001101010011011000110111001110000011100101100001011000100110
00110110010001100101011001100110011101101000011010010110101001101011011011000
11011010110111001101111011100000111000101110010011100110111010001110101011101
10011101110111100001111001011110100010000100100001
01000001010000010010000011010001000100010101000110010001110100100000100100101001
01001001011010011000100110101001100010011110101000001010001010100100101001101
0101000101010101010101100101011101011000010110010101101000110000001100010011001
00011001100110100001101010011011000110111001110000011100101100001011000100110
00110110010001100101011001100110011101101000011010010110101001101011011011000
11011010110111001101111011100000111000101110010011100110111010001110101011101
10011101110111100001111001011110100010000100100001
01000001010000010010000011010001000100010101000110010001110100100000100100101001
01001001011010011000100110101001111010011110101000001010001010100100101001101
0101000101010101010101100101011101011000010110010101101000110000001100010011001
00011001100110100001101010011011000110111001110000011100101100001011000100110
00110110010001100101011001100110011101101000011010010110101001101011011011000
11011010110111001101111011100000111000101110010011100110111010001110101011101
10011101110111100001111001011110100010000100100001
01000001010000010010000011010001000100010101000110010001110100100000100100101001
01001001011010011000100110101001110110011110101000001010001010100100101001101
0101000101010101010101100101011101011000010110010101101000110000001100010011001
00011001100110100001101010011011000110111001110000011100101100001011000100110
00110110010001100101011001100110011101101000011010010110101001101011011011000
11011010110111001101111011100000111000101110010011100110111010001110101011101
10011101110111100001111001011110100010000100100001
01000001010000010010000011010001000100010101000110010001110100100000100100101001
01001001011010011000100110101001110000011110101000001010001010100100101001101
0101000101010101010101100101011101011000010110010101101000110000001100010011001
00011001100110100001101010011011000110111001110000011100101100001011000100110
00110110010001100101011001100110011101101000011010010110101001101011011011000
11011010110111001101111011100000111000101110010011100110111010001110101011101
10011101110111100001111001011110100010000100100001
01000001010000010010000011010001000100010101000110010001110100100000100100101001
01001001011010011000100110101001110011011110101000001010001010100100101001101
0101000101010101010101100101011101011000010110010101101000110000001100010011001
00011001100110100001101010011011000110111001110000011100101100001011000100110
00110110010001100101011001100110011101101000011010010110101001101011011011000
11011010110111001101111011100000111000101110010011100110111010001110101011101
10011101110111100001111001011110100010000100100001
01000001010000010010000011010001000100010101000110010001110100100000100100101001
01001001011010011000100110101001110010111110101000001010001010100100101001101
0101000101010101010101100101011101011000010110010101101000110000001100010011001
00011001100110100001101010011011000110111001110000011100101100001011000100110
00110110010001100101011001100110011101101000011010010110101001101011011011000
11011010110111001101111011100000111000101110010011100110111010001110101011101
10011101110111100001111001011110100010000100100001

0100000101000001001000001101000100010001010100011001000111010010000100100101001
01001001011010011000100110101001110010001110101000001010001010100100101001101
01010001010101010101100101011101011000010110010101101000110000001100010011001
00011001100110100001101010011011000110111001110000011100101100001011000100110
00110110010001100101011001100110011101101000011010010110101001101011011011000
11011010110111001101111011100000111000101110010011100110111010001110101011101
10011101110111100001111001011110100010000100100001
0100000101000001001000001101000100010001010100011001000111010010000100100101001
01001001011010011000100110101001110010010110101000001010001010100100101001101
01010001010101010101100101011101011000010110010101101000110000001100010011001
00011001100110100001101010011011000110111001110000011100101100001011000100110
00110110010001100101011001100110011101101000011010010110101001101011011011000
11011010110111001101111011100000111000101110010011100110111010001110101011101
10011101110111100001111001011110100010000100100001
0100000101000001001000001101000100010001010100011001000111010010000100100101001
01001001011010011000100110101001110010011010101000001010001010100100101001101
01010001010101010101100101011101011000010110010101101000110000001100010011001
00011001100110100001101010011011000110111001110000011100101100001011000100110
00110110010001100101011001100110011101101000011010010110101001101011011011000
11011010110111001101111011100000111000101110010011100110111010001110101011101
10011101110111100001111001011110100010000100100001
0100000101000001001000001101000100010001010100011001000111010010000100100101001
01001001011010011000100110101001110010011100101000001010001010100100101001101
01010001010101010101100101011101011000010110010101101000110000001100010011001
00011001100110100001101010011011000110111001110000011100101100001011000100110
00110110010001100101011001100110011101101000011010010110101001101011011011000
11011010110111001101111011100000111000101110010011100110111010001110101011101
10011101110111100001111001011110100010000100100001
0100000101000001001000001101000100010001010100011001000111010010000100100101001
01001001011010011000100110101001110010011111101000001010001010100100101001101
01010001010101010101100101011101011000010110010101101000110000001100010011001
00011001100110100001101010011011000110111001110000011100101100001011000100110
00110110010001100101011001100110011101101000011010010110101001101011011011000
11011010110111001101111011100000111000101110010011100110111010001110101011101
10011101110111100001111001011110100010000100100001
0100000101000001001000001101000100010001010100011001000111010010000100100101001
01001001011010011000100110101001110010011110001000001010001010100100101001101
01010001010101010101100101011101011000010110010101101000110000001100010011001
00011001100110100001101010011011000110111001110000011100101100001011000100110
00110110010001100101011001100110011101101000011010010110101001101011011011000
11011010110111001101111011100000111000101110010011100110111010001110101011101
10011101110111100001111001011110100010000100100001
0100000101000001001000001101000100010001010100011001000111010010000100100101001
01001001011010011000100110101001110010011110111000001010001010100100101001101
01010001010101010101100101011101011000010110010101101000110000001100010011001
00011001100110100001101010011011000110111001110000011100101100001011000100110
00110110010001100101011001100110011101101000011010010110101001101011011011000
11011010110111001101111011100000111000101110010011100110111010001110101011101
10011101110111100001111001011110100010000100100001
0100000101000001001000001101000100010001010100011001000111010010000100100101001
01001001011010011000100110101001110010011110100000001010001010100100101001101
01010001010101010101100101011101011000010110010101101000110000001100010011001
00011001100110100001101010011011000110111001110000011100101100001011000100110
00110110010001100101011001100110011101101000011010010110101001101011011011000
11011010110111001101111011100000111000101110010011100110111010001110101011101
10011101110111100001111001011110100010000100100001

01000001010000010010000011010001000100010101000110010001110100100000100100101001
01001001011010011000100110101001110010011110101100001010001010100100101001101
01010001010101010101100101011101011000010110010101101000110000001100010011001
00011001100110100001101010011011000110111001110000011100101100001011000100110
00110110010001100101011001100110011101101000011010010110101001101011011011000
11011010110111001101111011100000111000101110010011100110111010001110101011101
1001110111011110000111100101111010001000010010000 1
01000001010000010010000011010001000100010101000110010001110100100000100100101001
01001001011010011000100110101001110010011110101010001010001010100100101001101
01010001010101010101100101011101011000010110010101101000110000001100010011001
00011001100110100001101010011011000110111001110000011100101100001011000100110
00110110010001100101011001100110011101101000011010010110101001101011011011000
11011010110111001101111011100000111000101110010011100110111010001110101011101
10011101110111100001111001011110100010000100100001
01000001010000010010000011010001000100010101000110010001110100100000100100101001
01001001011010011000100110101001110010011110101001001010001010100100101001101
01010001010101010101100101011101011000010110010101101000110000001100010011001
00011001100110100001101010011011000110111001110000011100101100001011000100110
00110110010001100101011001100110011101101000011010010110101001101011011011000
11011010110111001101111011100000111000101110010011100110111010001110101011101
10011101110111100001111001011110100010000100100001
01000001010000010010000011010001000100010101000110010001110100100000100100101001
01001001011010011000100110101001110010011110101000101010001010100100101001101
01010001010101010101100101011101011000010110010101101000110000001100010011001
00011001100110100001101010011011000110111001110000011100101100001011000100110
00110110010001100101011001100110011101101000011010010110101001101011011011000
11011010110111001101111011100000111000101110010011100110111010001110101011101
10011101110111100001111001011110100010000100100001
