# Supplementary material for: Hybrid encryption technique: Integrating the neural network with distortion techniques
Source: PLoS One. 2022 Sep 28;17(9):e0274947. doi: 10.1371/journal.pone.0274947 (PMC9518910; doi:10.1371/journal.pone.0274947)
Supplement: S1 Table — (PDF) [file pone.0274947.s002.pdf]

item	value
Number of Layers	2
Number of inputs	8
Number of Hidden Layers	1
Number of neurons in hidden Layer	8
Type of Transfer Function	TANSIG
Number of neurons in the output layer	8
Training Algorithm	Genetic Algorithm
Training Algorithm Specifications	Population size=100
	String Length=20
	Crossover probability=0.3
	Mutation probability=0.001
Performance metric	Mean Square Error
Ratio of training	0.7
Ratio of validation	0.15
Ratio of testing	0.15
