# Supplementary figures and images for: Hybrid encryption technique: Integrating the neural network with distortion techniques
Source: PLoS One. 2022 Sep 28;17(9):e0274947. doi: 10.1371/journal.pone.0274947 (PMC9518910; doi:10.1371/journal.pone.0274947)

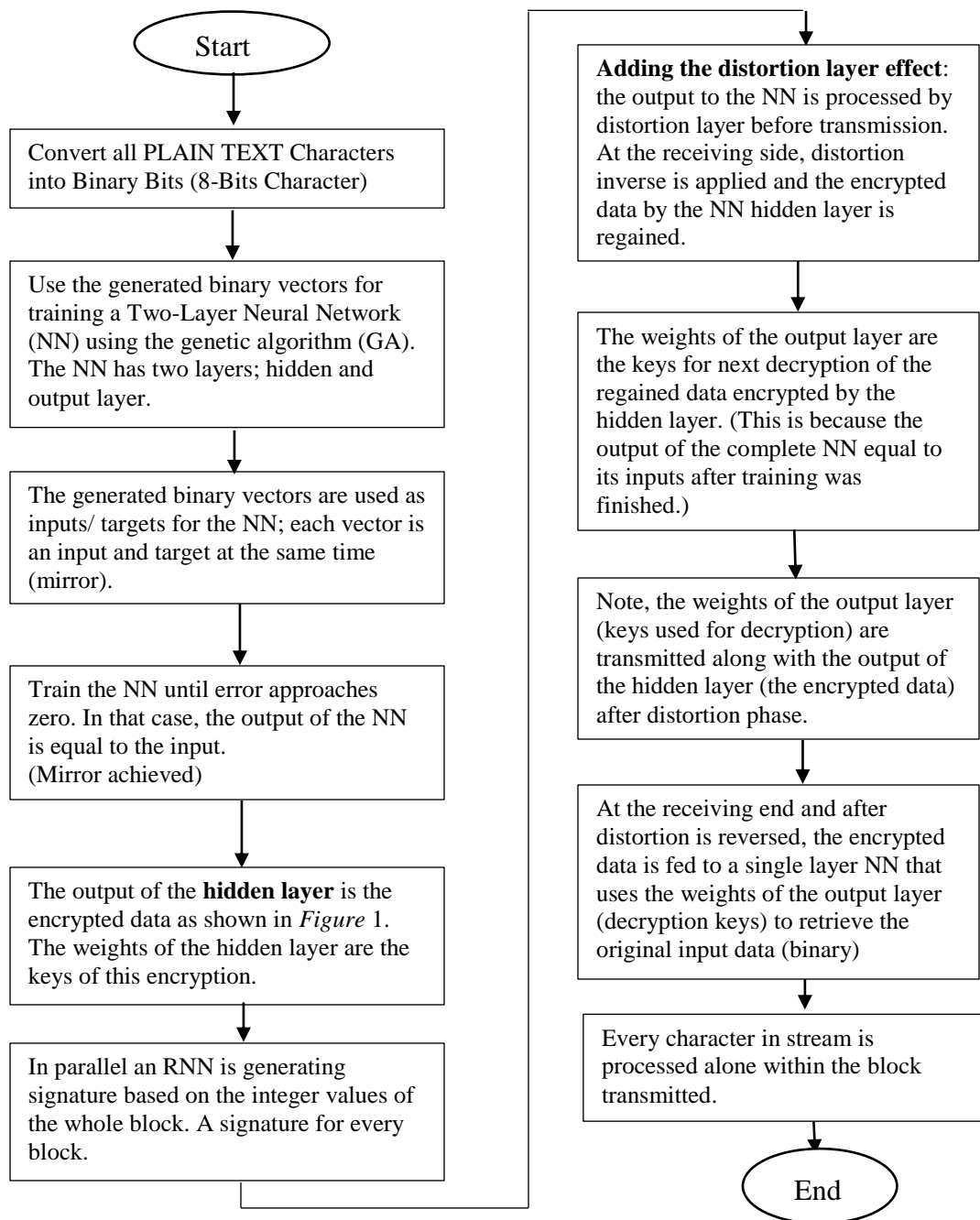

Supplement: S1 Fig — (PDF) [file pone.0274947.s003.pdf]
